# Design and synthesis of a 4-aminoquinoline-based molecular tweezer that recognizes protoporphyrin IX and iron(iii) protoporphyrin IX and its application as a supramolecular photosensitizer†
†Electronic supplementary information (ESI) available. See DOI: 10.1039/c8sc02133c.


**DOI:** 10.1039/c8sc02133c

**Published:** 2018-08-31

**Authors:** Yosuke Hisamatsu, Naoki Umezawa, Hirokazu Yagi, Koichi Kato, Tsunehiko Higuchi

**Affiliations:** a Graduate School of Pharmaceutical Sciences , Nagoya City University , 3-1 Tanabe-dori, Mizuho-ku , Nagoya 467-8603 , Japan . Email: hisamatsu@phar.nagoya-cu.ac.jp ; Email: higuchi@phar.nagoya-cu.ac.jp; b Exploratory Research Center on Life and Living Systems (ExCELLS) and Institute for Molecular Science (IMS) , National Institutes of Natural Sciences , 5-1 Higashiyama, Myodaiji , Okazaki 444-8787 , Japan

## Abstract

We report on the design and synthesis of a new type of 4-aminoquinoline-based molecular tweezer **1** which forms a stable host–guest complex with protoporphyrin IX (PPIX) *via* multiple interactions in a DMSO and HEPES buffer (pH 7.4) mixed solvent system. The binding constant for the 1 : 1 complex (*K*_11_) between **1** and PPIX is determined to be 4 × 10^6^ M^–1^. Furthermore, **1** also forms a more stable complex with iron(iii) protoporphyrin IX (Fe(iii)PPIX), the *K*_11_ value for which is one order of magnitude greater than that for PPIX, indicating that **1** could be used as a recognition unit of a synthetic heme sensor. On the other hand, the formation of the stable PPIX·**1** complex (supramolecular photosensitizer) prompted us to apply it to photodynamic therapy (PDT). Cell staining experiments using the supramolecular photosensitizer and evaluations of its photocytotoxicity indicate that the PDT activity of PPIX is improved as the result of the formation of a complex with **1**.

## Abstract 2

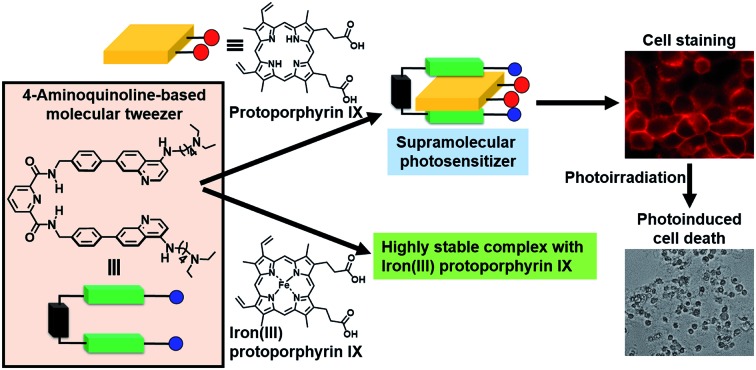
A 4-aminoquinoline-based molecular tweezer was developed as a synthetic receptor for protoporphyrin IX and iron(iii) protoporphyrin IX, and applied as a supramolecular photosensitizer.

## Introduction

Supramolecular chemistry and host–guest chemistry have primarily drawn their inspirations from molecular interactions of biomolecules such as proteins, DNA, RNA, lipids, and related molecules.^1^,^2^ Over the past three decades, a variety of synthetic host–guest systems have been developed. The precise recognition and detection of biologically important molecules in aqueous media by synthetic host molecules have remained a great challenge.^3^–^10^

**Scheme 1:**
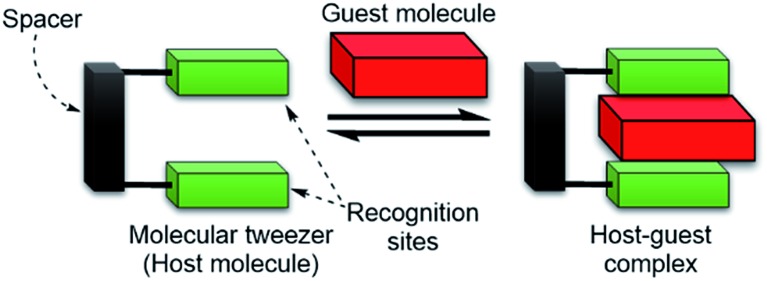
Cartoon representation of a molecular tweezer (host molecule) that recognizes an aromatic guest molecule.

Molecular tweezers are defined as synthetic receptors that contain two flat, generally aromatic, recognition sites separated by rigid or partly rigid spacers (Scheme 1).^11^,^12^ The spacer functions to maintain the distance between the two recognition sites at *ca.* 7 Å, which is a suitable distance for the formation of π-sandwich complexes with aromatic guest molecules. Although a variety of molecular tweezers have been reported to date,^11^–^21^ there are a few examples of molecular tweezers that function as receptors for biomolecules.^6^,^11^,^22^–^25^

**Scheme 2:**
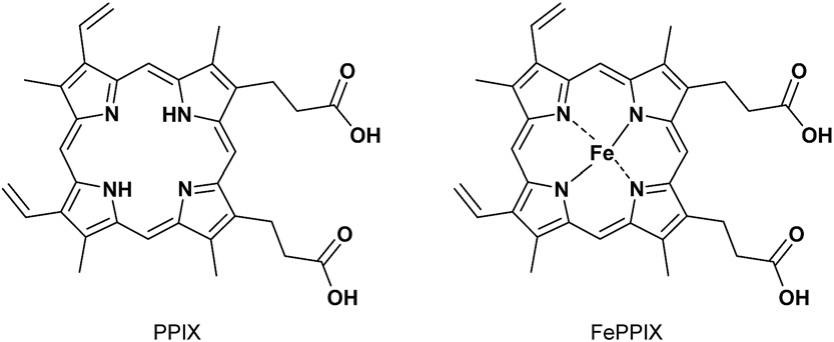
Structures of PPIX and FePPIX.

Among the biologically important molecules, protoporphyrin IX (PPIX) and its iron (Fe) complex containing a broad π-conjugated plane and two propionic acids are attractive guest molecules for molecular tweezers (Scheme 2). PPIX is ubiquitously present in living cells in small amounts as a precursor of heme and is a well-known naturally occurring photosensitizer.^26^ Although PPIX-based strategies including 5-aminolevulinic acid (5-ALA) have been used for photodynamic therapy (PDT) and photodynamic diagnosis (PDD),^27^,^28^ the direct use of PPIX has been limited due to its tendency to undergo aggregation and poor solubility in aqueous solutions.^29^,^30^

While heme (FePPIX) plays important roles in numerous biological systems as a cofactor of hemoproteins,^31^ the intracellular concentrations of protein non-bound labile heme need to be controlled because of its cytotoxicity.^32^,^33^ Recent studies have pointed out the importance of labile heme as a signaling molecule that participates in a broad range of cellular events containing gene expression, ion channel function, differentiation in cells and related processes.^31^,^34^–^37^ However, our knowledge of the cellular mechanisms of labile heme and its relevance for various diseases remains limited. Thus, it appears that fluorescent heme sensors should be useful tools for studies concerning the chemical biology of labile heme.^38^ The examples of fluorescent heme sensors have been limited to genetically encoded heme binding proteins bearing fluorescent proteins or dyes.^38^–^43^

Our research interest is developing useful host molecules with the potential to serve as synthetic receptors for PPIX and Fe(iii)PPIX, and their biological applications as synthetic fluorescent heme sensors, supramolecular photosensitizers, *etc.* As a first step, we report herein on the design and synthesis of a new type of molecular tweezer **1** that forms stable complexes with both PPIX and Fe(iii)PPIX and its applications as a supramolecular photosensitizer. The results of this study are briefly summarized as follows: (i) the design and synthesis of the molecular tweezer **1** and reference compounds **2–4**, (ii) binding studies of **1** and reference compounds **2–4** for PPIX, (iii) binding studies of **1** for Fe(iii)PPIX, ZnPPIX and flavin mononucleotide (FMN), (iv) cell staining of the supramolecular complex formed from PPIX and **1**, and its application as a supramolecular photosensitizer for PDT.

## Results and discussion

### Design and synthesis of molecular tweezer **1** and reference compounds **2–4**

**Scheme 3:**
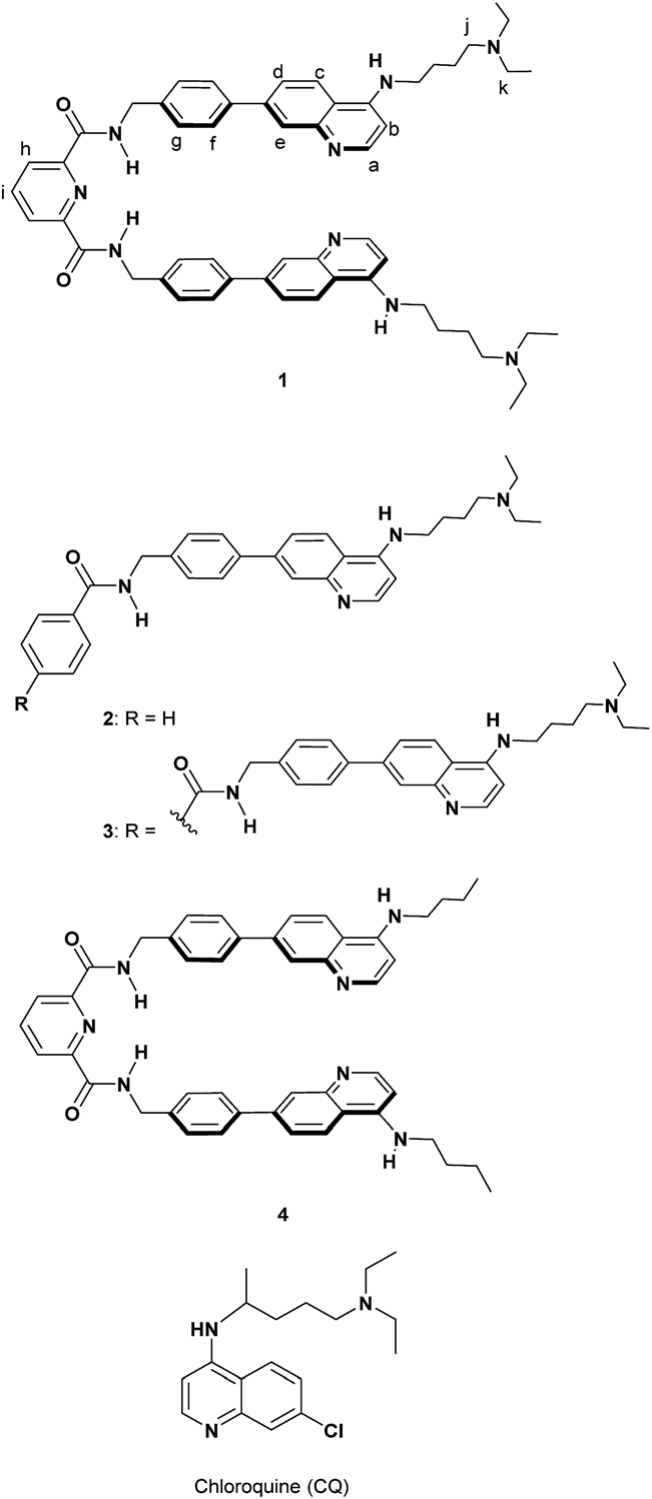
Structures of **1–4** and chloroquine (CQ).

The crystal structure of human heme oxygenase-1 (HO-1) (PDB ID: 1N45)^44^ with heme indicates that HO-1 interacts with the porphyrin moiety of heme by hydrophobic interactions and the formation of a coordination bond between Fe and a histidine residue into a hydrophobic pocket. Furthermore, basic residues of amino acids (lysine and arginine) of HO-1 surround two propionates of heme and can interact with them *via* electrostatic interactions. The elegant recognition system of HO-1 toward heme prompted us to design and synthesize the molecular tweezer **1** having an appropriate hydrophobic space to precisely recognize both PPIX and Fe(iii)PPIX (Scheme 3).

Formation of complexes between Fe(iii)PPIX and antimalarial 4-aminoquinoline derivatives containing chloroquine (CQ) (Scheme 3) has been examined so far.^45^–^48^ We recently reported on the development of some molecules combining 4-amino-7-chloroquinoline with various π-conjugated planar moieties and their potent antimalarial activity.^49^ Thus, the 4-aminoquinoline moiety was employed as two recognition sites that interact with the broad π-conjugated plane of porphyrins *via* π–π stacking and hydrophobic interactions.^50^

Two tertiary amino groups located at the terminal of alkyl side chains on the quinoline parts of **1** would be protonated at physiological pH and should then interact with the carboxylate ions of PPIX *via* electrostatic interactions and hydrogen bonding.^46^,^50^ As the spacer unit of the molecular tweezer (Scheme 1), we used a pyridine 2,6-dicarboxamide unit that participates in intramolecular hydrogen bonding with the pyridine nitrogen favouring a relatively rigid *syn*,*syn* conformation of the NH groups.^18^,^21^,^51^–^53^

**Scheme 4:**
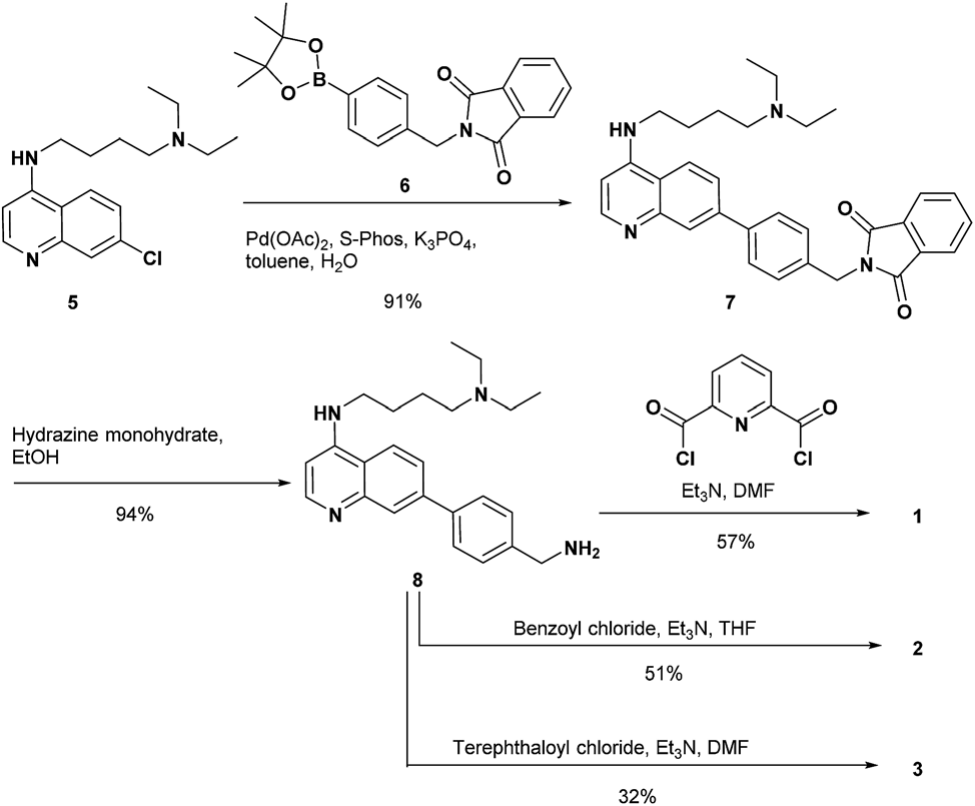
Synthesis of **1–3**.

The synthesis of **1** and the reference compounds **2** and **3** is shown in Scheme 4. Compound **4** was also prepared following a similar procedure (Scheme S1 in the ESI†). The Suzuki–Miyaura cross coupling reaction of the 4-amino-7-chloroquinoline derivative **5** (ref. 54) with the boronate ester **6** gave **7**.^55^ After the reaction of **7** with hydrazine monohydrate, the resulting amine derivative **8** was reacted with 2,6-pyridinedicarbonyl dichloride to give **1**. Compounds **2** and **3** were prepared from the reaction of the corresponding acid chloride and **8**, respectively.

### UV-Vis spectral measurements of **1** and **2** in DMSO/100 mM buffer (from pH 5.5 to 10.7) = 1 : 4 (v/v)

The tertiary amines at the terminal positions of **1** and **2** would be protonated at physiological pH because the p*K*_a_ values of their conjugate acids are *ca.* 10.^56^ UV-Vis spectral measurements of **1** and **2** at various pH values in DMSO/H_2_O = 1 : 99 (v/v) solutions were carried out to determine the p*K*_a_ values of conjugated acids of the quinoline nitrogen atoms. However, this was not successful probably due to the formation of aggregates of **1** and **2** in the alkaline pH solutions (data not shown). The p*K*_a_ values of **1** and **2** were roughly estimated in DMSO/100 mM buffer (from pH 5.5 to 10.7) = 1 : 4 (v/v) (Fig. S1 in the ESI†). On the basis of the pH dependent change in the UV-Vis spectra of **1**, the p*K*_a_ values of the two conjugate acids of the quinoline nitrogen atoms on **1** were estimated to be between 7.4 and 8.6 (Fig. S1a in the ESI†). The p*K*_a_ value of the conjugate acid of the quinoline nitrogen atom on **2** was determined to be between 8.2 and 8.6 (Fig. S1b in the ESI†). The range of estimated p*K*_a_ values for **1** and **2** are in agreement with the reported values for related 4-aminoquinoline analogues.^56^–^58^ These results suggest that the major species of **1** at pH 7.4 is the tetra-protonated form of **1**, in which the two tertiary amines and the nitrogen atoms on the two quinoline rings are protonated (Fig. S2 in the ESI†).^56^–^58^ The major species of **2** at pH 7.4 would be predicted to be the bis-protonated form (Fig. S2 in the ESI†).

### Binding studies of **1** and the reference compounds for PPIX

**Fig. 1:**
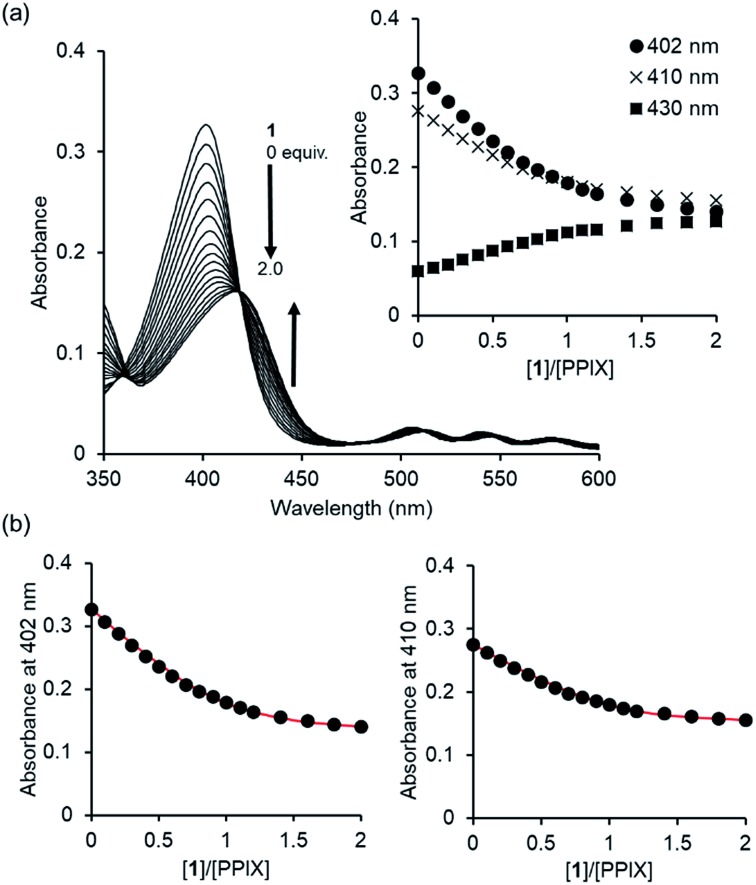
(a) UV-Vis titration of PPIX (2.4 μM) with **1** in DMSO/33 mM HEPES buffer (pH 7.4) = 2 : 3 (v/v) at 25 °C. Inset: changes in the absorbance of PPIX upon the addition of **1**. (b) Nonlinear least-squares fitting of UV-Vis titration curves at 402 nm and 410 nm, respectively. The corresponding fitted curves using a 1 : 1 binding model are shown as solid red lines.

Since PPIX is present in the form of complicated aggregate states under physiological conditions,^29^,^30^ the complexation ability of **1** toward PPIX was evaluated *via* UV-Vis titration experiments of PPIX (2.4 μM) in DMSO/33 mM HEPES buffer (pH 7.4) = 2 : 3 (v/v) at 25 °C (Fig. 1). Under these conditions, PPIX should largely exist as a monomer.^59^ When the amount of **1** was increased (0–2.0 equiv.), absorbance of the Soret band (*ca.* 402 nm) of PPIX decreased and was red-shifted from *ca.* 402 nm to *ca.* 417 nm. Global fitting of the data (absorbance at 402 nm, 410 nm and 430 nm) to a 1 : 1 binding model using the Bindfit program, a non-linear least-squares fitting program, provided good fitting results (Fig. 1b and S3 in the ESI†).^60^,^61^ The binding constant for a 1 : 1 complex (*K*_11_) was determined to be (4.0 ± 1.0) × 10^6^ M^–1^, which is reported as the mean ± standard deviation of three independent experiments (Table 1).^62^ The formation of a 1 : 1 complex between PPIX and **1** was supported by the Job plot (Fig. S5a in the ESI†).^63^ In addition, a molecular ion peak of the PPIX·**1** complex (*m*/*z*: 1446.8, [PPIX·**1** + H]^+^) was observed in an ESI-mass spectrum (Fig. S6 in the ESI†). The fluorescence titration of PPIX (excitation at 402 nm) with **1** was also carried out in the same solvent system. A decrease in emission intensity at *ca.* 628 nm of PPIX (1.0 μM) and a small red shift of emission maxima from 628 nm to 635 nm were observed upon the addition of **1** (0–3.6 equiv.) (Fig. S7 in the ESI†). The *K*_11_ value of the PPIX·**1** complex determined from the fluorescence titration was (3.1 ± 1.0) × 10^6^ M^–1^, which is the same degree as that determined by UV-Vis titration (Table 1).

**Table 1:** Binding constants of **1–4** for PPIX determined by UV-Vis titration in DMSO/33 mM HEPES buffer (pH 7.4) = 2 : 3 (v/v) at 25 °C

Host molecule	*K*_11_ ^*a*^/M^–1^	*K*_12_ ^*a*^/M^–1^
**1**	(4.0 ± 1.0) × 10^6^	—
**2**	(1.9 ± 0.4) × 10^4^	—
**3**	(4.0 ± 0.2) × 10^5^	—
**4**	(1.5 ± 0.4) × 10^6^	(2.3 ± 0.6) × 10^6^^*b*^

^*a*^The data are reported as the mean ± standard deviation of three independent experiments.

^*b*^PPIX·(**4**)_2_ complex.

**Fig. 2:**
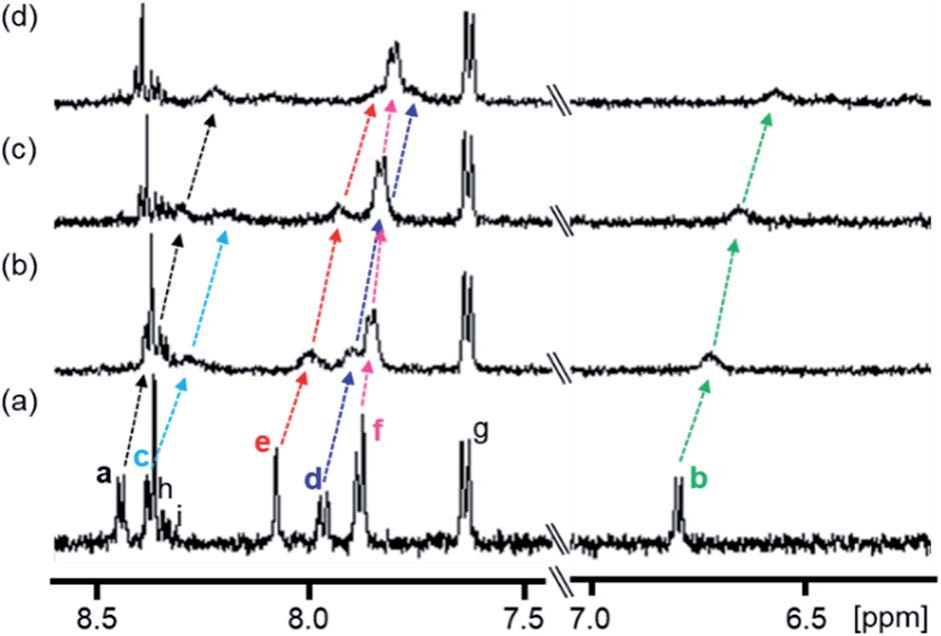
Partial ^1^H NMR spectra (500 MHz) of **1** in the absence and presence of PPIX in DMSO-*d*_6_/33 mM HEPES buffer (pD 7.4) = 2 : 1 (v/v) at 23 °C. (a) **1** (0.10 mM), (b) **1** (0.10 mM) + PPIX (0.020 mM), (c) **1** (0.10 mM) + PPIX (0.050 mM) and (d) **1** (0.10 mM) + PPIX (0.10 mM). 1% tetramethylsilane in CCl_4_ was used as the external reference. Peak assignments of aromatic protons H^a^–H^i^ on **1** are listed in Scheme 3.

The complexation-induced chemical shift changes for **1** in the presence of PPIX were monitored by ^1^H NMR spectroscopy in DMSO-*d*_6_/33 mM HEPES buffer pD 7.4 = 2 : 1 (v/v) (Fig. 2).^64^ The peak assignments of the aromatic protons H^a^–H^i^ of **1** (Scheme 3 and Fig. 2a) were carried out based on the results of ^1^H–^1^H COSY and ROESY measurements (Fig. S8 and S9 in the ESI†).

The ^1^H NMR spectrum of **1** (0.10 mM) is shown in Fig. 2a. Upon the addition of PPIX (1.0 equiv.), upfield shifts of H^a^–H^f^ proton signals (H^a^; Δ*δ* = *ca.* 0.22 ppm, H^b^; Δ*δ* = *ca.* 0.23 ppm, H^d^; Δ*δ* = *ca.* 0.20 ppm, H^e^; Δ*δ* = *ca.* 0.23 ppm, H^f^; Δ*δ* = *ca.* 0.08 ppm) on **1** were observed (Fig. 2) while the H^g^–H^i^ proton signals showed negligible shifts. The upfield shifts of H^a^–H^f^ protons suggest the presence of π–π stacking interactions between the quinoline moieties of **1** and the π-plane of PPIX.^65^ Furthermore, aliphatic protons H^j^ and H^k^ located on neighboring ammonium ions exhibited small up-field shifts (H^j^; Δ*δ* = *ca.* 0.06 ppm and H^k^; Δ*δ* = *ca.* 0.02 ppm) upon the addition of PPIX (1.0 equiv.) (Fig. S10 in the ESI†), probably due to interactions between ammonium ions of **1** and carboxylate ions of PPIX.^66^,^67^

**Fig. 3:**
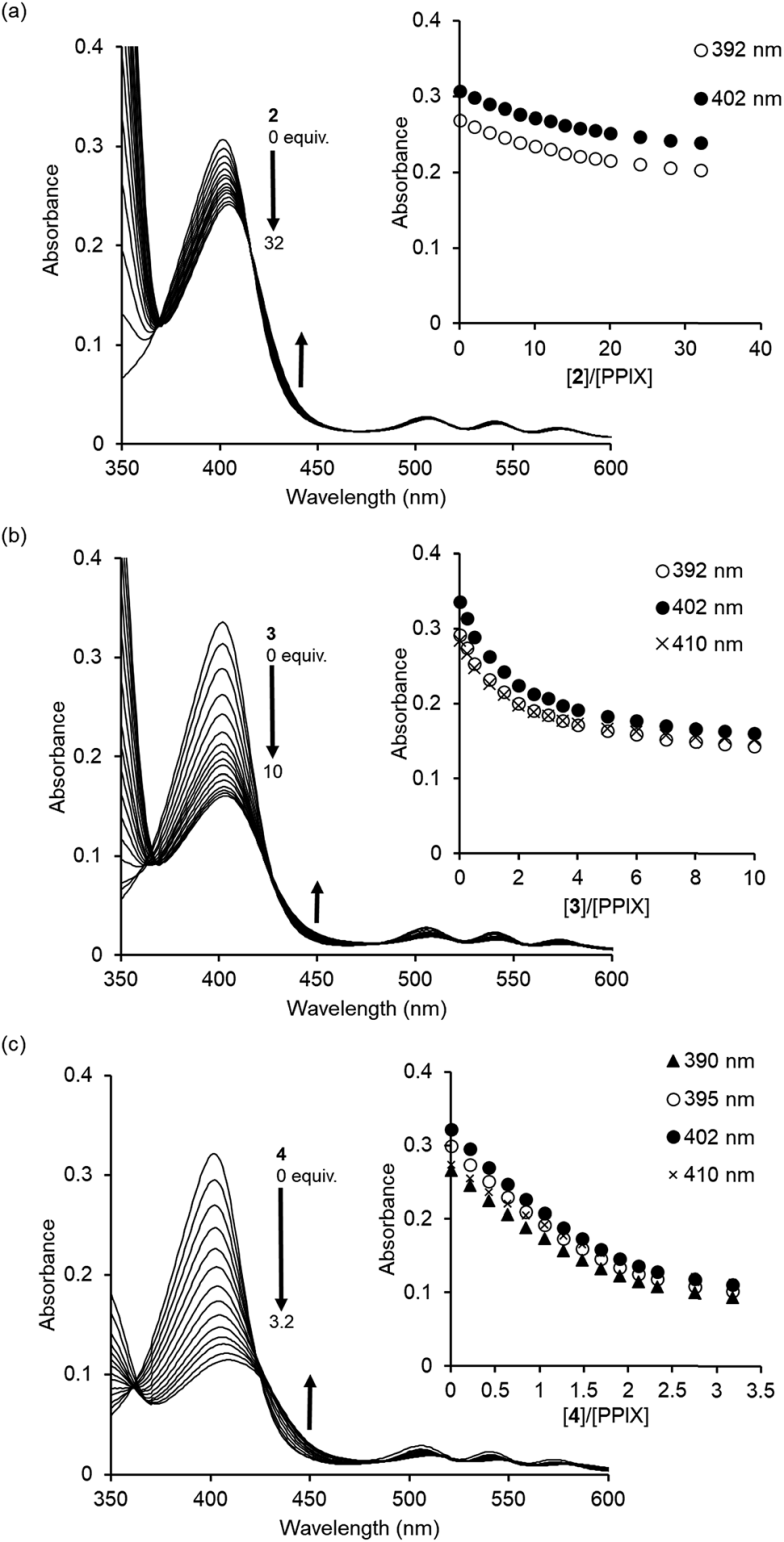
UV-Vis titrations of PPIX (2.4 μM) with (a) **2**, (b) **3**, and (c) **4** in DMSO/33 mM HEPES buffer (pH 7.4) = 2 : 3 (v/v) at 25 °C. Insets: changes in the absorbance of PPIX upon the addition of compounds **2–4**.

The complexation ability of reference compound **2** (containing a single 4-aminoquinoline moiety) with PPIX was evaluated by UV-Vis titration under the same conditions as were used for **1**. As shown in Fig. 3a, the UV-Vis spectra of PPIX show a small spectral change upon the addition of **2** (0–32 equiv.) as compared with that for **1**. The titration curves at 392 nm and 402 nm (inset of Fig. 3a) were not saturated despite the addition of a large excess of **2** (32 equiv.). The *K*_11_ value was determined to be (1.9 ± 0.4) × 10^4^ M^–1^ (Fig. S11 in the ESI†), which is *ca.* 200-fold lower than that of PPIX·**1** (Table 1).^68^ The weak interactions between **2** and PPIX were supported by ^1^H NMR measurements. The complexation-induced chemical shift changes in aromatic protons of **2** (0.10 mM) in the presence of PPIX (1.0 equiv.) were less than 0.04 ppm (Fig. S12 in the ESI†). These results show that the complexation ability of **2** is not sufficient for it to serve as a synthetic receptor for PPIX.

Compound **3** contains a terephthalamide spacer, in which the distance between the two 4-aminoquinoline moieties is longer than that in **1**. The formation of a PPIX·**3** complex was confirmed by UV-Vis titration (Fig. 3b). The results of the Job plot of PPIX with **3** support the conclusion that the stoichiometry of the complex should be 1 : 1 (or 2 : 2) (Fig. S5b in the ESI†). The molecular ion peak for a 1 : 1 complex of **3** and PPIX ([PPIX·**3** + H]^+^ = 1445.7) was observed in the ESI-mass spectrum (Fig. S13 in the ESI†).^69^ The titration curves (absorbance at 392 nm, 402 nm and 410 nm) were fitted with a 1 : 1 binding model (Fig. S14 in the ESI†). The *K*_11_ value for PPIX·**3** was determined to be (4.0 ± 0.2) × 10^5^ M^–1^, which is *ca.* 10-fold lower than that of PPIX·**1** (Table 1).^70^ The above findings suggest that the pyridine 2,6-dicarboxamide moiety is suitable for use as a spacer of the molecular tweezer in comparison with the terephthalamide spacer.

A further binding study using **4** without tertiary amino groups was conducted to evaluate the contribution of the tertiary amino groups on **1** to the complexation with PPIX. As shown in Fig. 3c, a decrease and small red-shift (*ca.* 7 nm) in the absorption maxima of PPIX were observed upon increasing the amount of **4** (0–3.2 equiv.) and the absorbance of the absorption maximum at 409 nm of PPIX was *ca.* 0.11 in the presence of 3.2 equiv. of **4**. The hypochromic effect of the Soret band of PPIX in the presence of **4** (inset of Fig. 3c) is greater than that in the presence of **1** or **3** (insets of Fig. 1a and 3b). These results suggest that the binding mode of PPIX with **4** is different from that for **1** and **3**. Global fitting calculations using the absorbance of PPIX at 390 nm, 395 nm, 402 nm and 410 nm could not be fitted with either a 1 : 1 binding model or a 2 : 1 binding model (PPIX : **4** = 2 : 1). On the other hand, a 1 : 2 binding model (PPIX : **4** = 1 : 2) provided good fitting results for the titration curves (Fig. S15 in the ESI†). The binding constants of *K*_11_ and *K*_12_ were determined to be (1.5 ± 0.4) × 10^6^ M^–1^ and (2.3 ± 0.6) × 10^6^ M^–1^, respectively, indicating that two molecules of **4** can strongly bind to PPIX, although the equilibrium of complexation between PPIX and **4** is more complicated than that for **1** (Table 1).

Based on the results of UV-Vis titration studies of PPIX with **1** and **4** (Fig. 1 and 3c) and chemical shift changes in the aliphatic protons of **1** in the case of ^1^H NMR titration with PPIX (Fig. S10 in the ESI†), it may be concluded that electrostatic interactions and hydrogen bonding between ammonium ions of **1** and carboxylate ions of PPIX contribute to the formation of a simple 1 : 1 complex (PPIX·**1** complex) and slightly improved stability of the PPIX·**1** complex (Table 1), although hydrophobic interactions and π–π stacking interactions are dominant in the complexation between PPIX and **1**.

Taken together with the binding studies of **1–4** for PPIX (Fig. 1–3 and Table 1), the molecular tweezer **1** that forms a stable 1 : 1 complex with PPIX, is the best synthetic receptor for PPIX among the compounds tested.

**Fig. 4:**
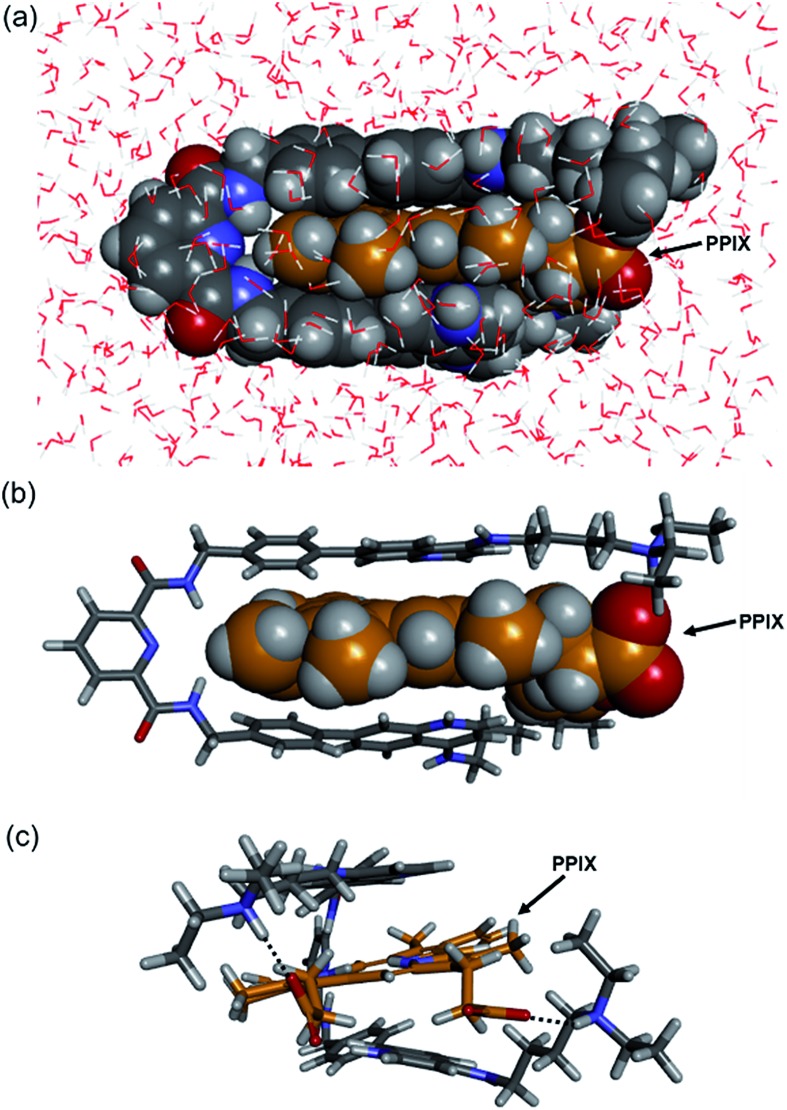
Structures of PPIX·**1** complex obtained by molecular mechanics calculation (Discovery Studio, CHARMM force field with solvation mode). The tetra-protonated form of **1** and bis-deprotonated form of PPIX were used for the calculation. (a) Front-view of PPIX·**1** complex (space-filling model). (b and c) Front-view and right-side view of PPIX·**1** complex (stick model) (H_2_O omitted for the sake of clarity).

The molecular mechanics calculation of the PPIX·**1** complex was performed using Discovery Studio (Biovia) with a CHARMM force field under the solvation mode. The tetra-protonated form of **1** and the bis-deprotonated form of PPIX were used for the calculation. As shown in Fig. 4, a π-sandwich structure of PPIX·**1** complex is suggested from the minimized structure of the PPIX·**1** complex (Fig. 4a and b). The distance between the quinoline ring and π-plane of PPIX is *ca.* 3.6 ± 0.2 Å, suggesting the presence of π–π stacking interactions. As shown in Fig. 4c, the presence of electrostatic interactions and hydrogen bonding between ammonium ions and carboxylate ions is also suggested.

### Binding studies of **1** and **2** for Fe(iii)PPIX, and evaluations of the complexation selectivity of **1** for Fe(iii)PPIX

**Fig. 5:**
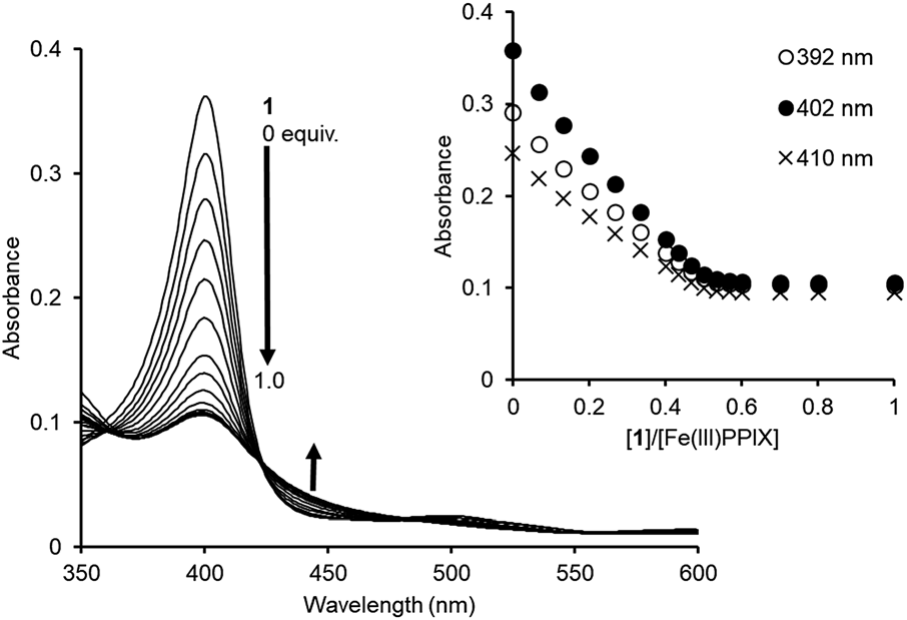
UV-Vis titration of Fe(iii)PPIX (2.4 μM) with **1** in DMSO/33 mM HEPES buffer (pH 7.4) = 2 : 3 (v/v) at 25 °C. Inset: changes in the absorbance of Fe(iii)PPIX upon the addition of **1**.

The binding studies of **1** for PPIX prompted us to perform a UV-Vis titration study of **1** against Fe(iii)PPIX (2.4 μM) in DMSO/33 mM HEPES buffer (pH 7.4) = 2 : 3 (v/v). Although the solvent system differs from the actual physical conditions, this solvent system has been extensively employed for conducting binding studies between Fe(iii)PPIX and quinoline antimalarials, since it has an advantage of maintaining Fe(iii)PPIX in a monomeric state at the concentration range used in UV-Vis spectral measurments.^45^–^47^,^71^–^73^ UV-Vis spectral changes of Fe(iii)PPIX (2.4 μM) upon the addition of **1** (0–1.0 equiv.) were monitored, as shown in Fig. 5. The decrease in the absorbance of the Soret band (*ca.* 402 nm) of Fe(iii)PPIX can be attributed to the formation of a π-stacked complex between Fe(iii)PPIX and **1**.^45^ The hypochromic effect of Fe(iii)PPIX is consistent with the previous results of UV-Vis titration with various 4-aminoquinoline derivatives.^44^–^46^,^71^,^72^ Interestingly, the change in the absorbance of Fe(iii)PPIX became saturated upon the addition of **1** at *ca.* 0.5–0.6 equiv., suggesting a complicated binding mode.^74^ The ESI-mass spectrum of **1** in the presence of Fe(iii)PPIX indicates the formation of both 1 : 1 (*m*/*z*: 1499.7, [Fe(iii)PPIX·**1**]^+^) and 2 : 1 (*m*/*z*: 2114.9, [(Fe(iii)PPIX)_2_·**1**–H]^+^) complexes, respectively (Fig. S17 in the ESI†).

Global fitting calculations using the absorbance of 10 different wavelengths of Fe(iii)PPIX could not be fitted with either the 1 : 1 binding model or the 1 : 2 binding model (Fe(iii)PPIX : **1** = 1 : 2). On the other hand, a 2 : 1 binding model (Fe(iii)PPIX : **1** = 2 : 1) gave better fitting results for the titration curves (Fig. S18 in the ESI†). Based on five independent experiments, the *K*_11_ and *K*_21_ values were determined to be >2 × 10^7^ M^–1^ and >2 × 10^6^ M^–1^, respectively, as lower limits (Table 2).^75^ To the best of our knowledge, the molecular tweezer **1** described herein exhibits the highest complexation ability for Fe(iii)PPIX under similar titration conditions.

**Table 2:** Binding constants determined by UV-Vis titrations in DMSO/33 mM HEPES buffer (pH 7.4) = 2 : 3 (v/v) at 25 °C

Guest molecule	Host molecule	*K*_11_/M^–1^	*K*_21_/M^–1^
Fe(iii)PPIX	**1**	>2 × 10^7^	>2 × 10^6^^*a*^
Fe(iii)PPIX	**2**	(1.4 ± 0.2) × 10^6^^*b*^	—
Fe(iii)PPIX	CQ	3.3 × 10^5^^*c*^	—
ZnPPIX	**1**	(5.1 ± 0.6) × 10^5^^*b*^	—
FMN	**1**	(3.7 ± 0.2) × 10^4^^*b*^	—

^*a*^(Fe(iii)PPIX)_2_·**1** complex.

^*b*^Values are the mean ± standard deviation of three independent experiments.

^*c*^ Ref. 45.

The complexation of **1** (0.10 mM) with Fe(iii)PPIX was also evaluated by ^1^H NMR titration in DMSO-*d*_6_/33 mM HEPES buffer (pD 7.4) = 2 : 1 (v/v) (Fig. S19 in the ESI†).^64^ Upon the addition of Fe(iii)PPIX (0–1.0 equiv.), the proton signals on **1** were broadened due to interaction with the paramagnetic Fe(iii)PPIX. The broadening of the aromatic proton signals for **1** in the formation of a complex with Fe(iii)PPIX is in agreement with previously reported results.^47^

The *K*_11_ value for the binding of CQ to Fe(iii)PPIX under the same conditions was reported to be 3.3 × 10^5^ M^–1^.^45^ Furthermore, the *K*_11_ value for the binding of **2** to Fe(iii)PPIX was determined to be (1.4 ± 0.2) × 10^6^ M^–1^ which is at least 10-fold less than that of **1** (Table 2) (Fig. S20 in the ESI†). A 1 : 1 binding mode of the complex between Fe(iii)PPIX and **2** is suggested by the Job plot (Fig. S21 in the ESI†). These results indicate the usefulness of the tweezer-type molecule like **1** as a synthetic receptor for Fe(iii)PPIX.^76^

The higher complexation ability of **1** and **2** towards Fe(iii)PPIX (Table 2) than that towards PPIX (Table 1) may be partly explained by enhanced π-interactions^77^ between the Fe(iii)-coordinated porphyrin π-plane and quinoline moieties. Considering the reported crystal structure of a complex between Ga(iii)PPIX and CQ,^50^ an axial ligand such as OH^–^ or H_2_O, which coordinates with the Fe(iii) ion of Fe(iii)PPIX, could contribute to the stability of the complex through hydrogen bonding between the axial ligand and a H-donor of the protonated nitrogen on the quinoline ring of **1** without loss of π–π stacking interactions.

**Fig. 6:**
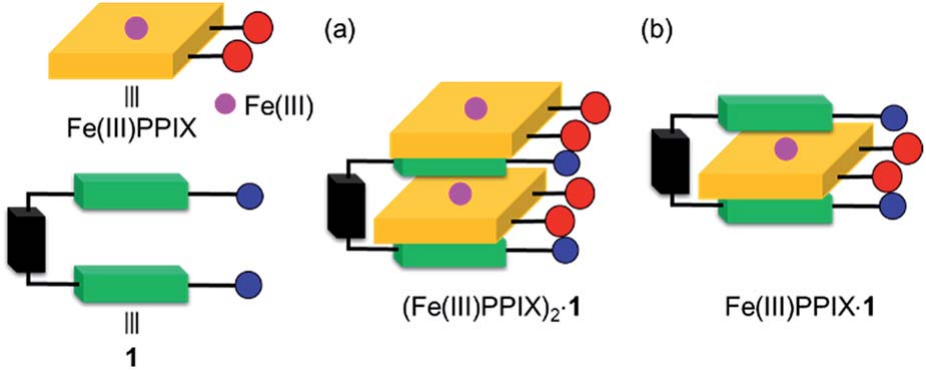
Schematic representation of the proposed structures of (a) (Fe(iii)PPIX)_2_·**1** (2 : 1 complex) and (b) Fe(iii)PPIX·**1** (1 : 1 complex).

The estimated *K*_21_ value of the (Fe(iii)PPIX)_2_·**1** complex is >2 × 10^6^ M^–1^, which is lower than the *K*_11_ value of the Fe(iii)PPIX·**1** complex (Table 2). It may be possible that a second molecule of Fe(iii)PPIX interacts with one of the free sides of the quinoline moieties of the Fe(iii)PPIX·**1** complex (Fig. 6), especially in the presence of an excess amount of Fe(iii)PPIX to **1**.^78^ Similar 2 : 1 complex modes between molecular tweezers and corresponding guest molecules have been reported for other host–guest systems.^79^–^81^

**Scheme 5:**
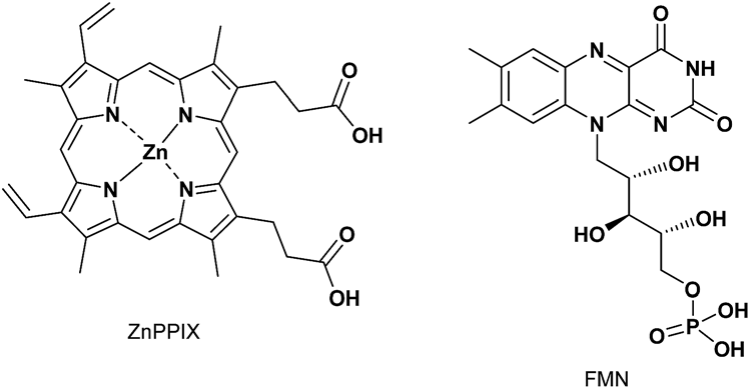
Structures of ZnPPIX and FMN.

To discuss the selective complexation ability of **1** towards Fe(iii)PPIX, the complexation of **1** with ZnPPIX (Scheme 5) was assessed by UV-Vis titration (Fig. S23 in the ESI†). The *K*_11_ value of **1** with ZnPPIX was determined to be (5.1 ± 0.6) × 10^5^ M^–1^ (Fig. S23 in the ESI†), which indicates lower affinity than for the PPIX·**1** complex (Table 2). The formation of the 1 : 1 complex between ZnPPIX and **1** was indicated by the Job plot (Fig. S24 in the ESI†). The complexation of ZnPPIX with **1** was also confirmed by ^1^H NMR titration and ESI mass measurement (Fig. S25 and S26 in the ESI†).^82^

As another biomolecule containing a broad π-conjugated plane with an acidic side chain, a binding study of FMN (Scheme 5) with **1** was evaluated. UV-Vis titration of FMN (30 μM) with **1** (Fig. S27 in the ESI†) revealed that the *K*_11_ value of the FMN·**1** complex was (3.7 ± 0.2) × 10^4^ M^–1^, which is significantly lower than the values for complexes between PPIX analogues and **1** (Table 2). These results support the conclusion that **1** can function as a synthetic receptor especially for Fe(iii)PPIX from the point of view of complexation ability and selectivity.

### Cell staining of the supramolecular complex formed from PPIX and **1**, and its application as a supramolecular photosensitizer for PDT

Photodynamic therapy (PDT) has received considerable attention as a safe, minimally invasive and tissue selective treatment for cancer and other diseases.^27^,^28^,^83^–^85^ Currently, several porphyrin derivatives have been approved in clinical use as photosensitizers. Naturally occurring 5-ALA is metabolized *via* the heme biosynthesis pathway to afford photoactive PPIX and is used as the prodrug of PPIX for PDT and PDD. It should be noted that the rapid clearance rate of endogenous PPIX reduces the side effects of PDT, such as skin photosensitivity.^83^–^85^As mentioned above, the fact that PPIX forms aggregates in aqueous solutions reduces its efficacy as a photosensitizer.^30^ Thus, modifications of PPIX and the utilization of nanoparticle carrier systems containing PPIX have been examined in attempts to suppress aggregation and improve the efficacy for PDT.^30^,^86^–^90^

We anticipated that the physical properties and PDT activity of PPIX could be controlled by formation of a complex with **1**. To confirm the assumption, the photocytotoxicity of PPIX in the presence of **1** was examined.

**Fig. 7:**
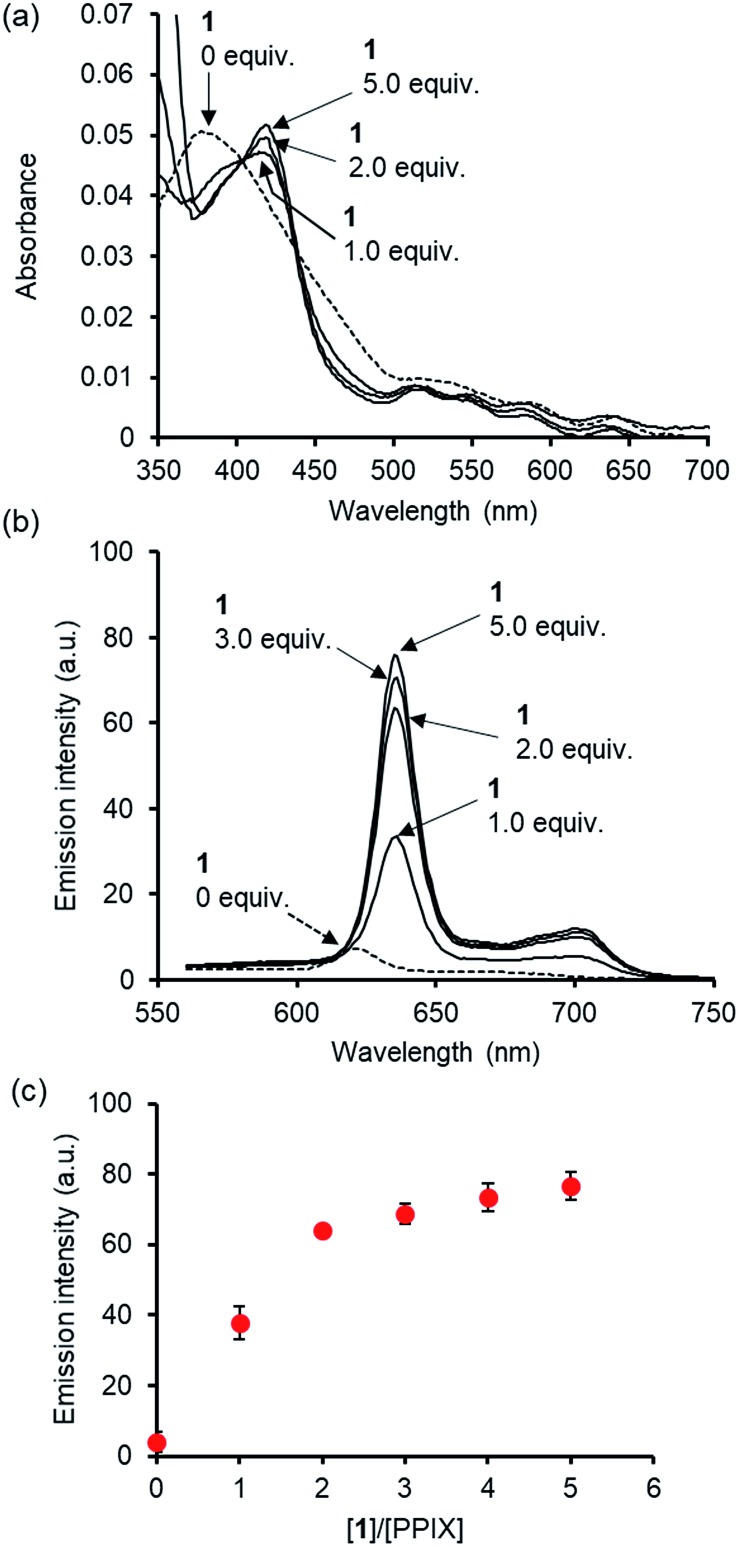
(a) Typical UV-Vis spectra and (b) typical fluorescence emission spectra (excitation at 545 nm) of PPIX (1.0 μM) in the absence and presence of **1** in DMSO/DMEM = 1 : 99 (v/v) at 25 °C. (c) Changes in mean emission intensity at 635 nm of PPIX in the presence of **1**. The data represent the mean ± standard deviation of three independent experiments.

Before conducting experiments using cancer cells, the UV-Vis and fluorescence emission spectra of PPIX (1.0 μM) in the absence and presence of **1** in DMSO/DMEM (Dulbecco's modified Eagle's medium) = 1 : 99 (v/v) were collected (Fig. 7). DMEM containing HEPES and no phenol red was used as the medium. The UV-Vis spectrum of PPIX alone (1.0 μM) was broadened and its absorption maximum appeared at *ca.* 377 nm due to aggregate formation (Fig. 7a).^29^,^30^ Dissociation of PPIX aggregates was observed in the presence of 1% Triton X-100 and the absorption maximum of PPIX was shifted from *ca.* 377 nm to 406 nm (Fig. S28 in the ESI†).

On the other hand, a red-shift in the absorption maxima of PPIX from *ca.* 377 nm to *ca.* 418 nm was observed when **1** (1.0, 2.0 and 5.0 μM) was present in the sample solution (Fig. 7a). Although the absorbance of PPIX at 1.0 μM is quite low, it is obvious that the Soret band (*ca.* 418 nm) of PPIX in the presence of **1** (2.0 or 5.0 μM) is almost identical to that (*ca.* 417 nm) in the presence of **1** (2.0 μM) in DMSO/33 mM HEPES buffer = 2 : 3 (v/v) (Fig. 1).^91^

The fluorescence emission spectrum of PPIX exhibited a very weak emission at 621 nm due to aggregate formation in DMSO/DMEM = 1 : 99 (v/v).^29^ The emission quenching of PPIX (*λ*_em_ = 621 nm) was substantially restored in the presence of 1% Triton X-100 and its emission maximum was observed at 632 nm. The increase in the emission intensity of PPIX in the presence of 1% Triton X-100 was *ca.* 130-fold greater than that of aggregated PPIX (Fig. S28 and S31 in the ESI†).^92^ Changes in the UV-Vis and fluorescence emission spectra of PPIX in DMSO/DMEM = 1 : 99 (v/v) suggest that PPIX exists mostly as a monomer in the presence of 1% Triton X-100.

As shown in Fig. 7b and c, a red-shift in the emission maxima of PPIX in DMSO/DMEM = 1 : 99 (v/v) from 621 nm to 635 nm and an enhancement in emission intensity were observed in the presence of **1** (1.0–5.0 equiv.). The changes in emission intensity at 635 nm of PPIX became saturated when *ca.* 3–4 equivalents of **1** were added (Fig. 7c). Both UV-Vis and fluorescence titration experiments in DMEM solutions clearly indicate that **1** is able to form a complex with PPIX and suppresses the self-aggregation of PPIX, leading to the apparent increase in the emission of PPIX.

Cell staining experiments of HCT-116 cells (human colon carcinoma cell line) in DMSO/DMEM = 1 : 99 (v/v) in the presence of PPIX with **1** were carried out. After incubating HCT-116 cells with PPIX (1.0 μM) alone or PPIX (1.0 μM) in the presence of **1** (2.0 or 5.0 equiv.) on a glass bottom dish for 1 h at 37 °C in a 5% CO_2_ atmosphere, followed by washing with cold PBS, the HCT-116 cells were observed by fluorescence microscopy (BZ-X710, Keyence) using a TRITC filter (Ex. 545 ± 13 nm, Em. 605 ± 35 nm).

**Fig. 8:**
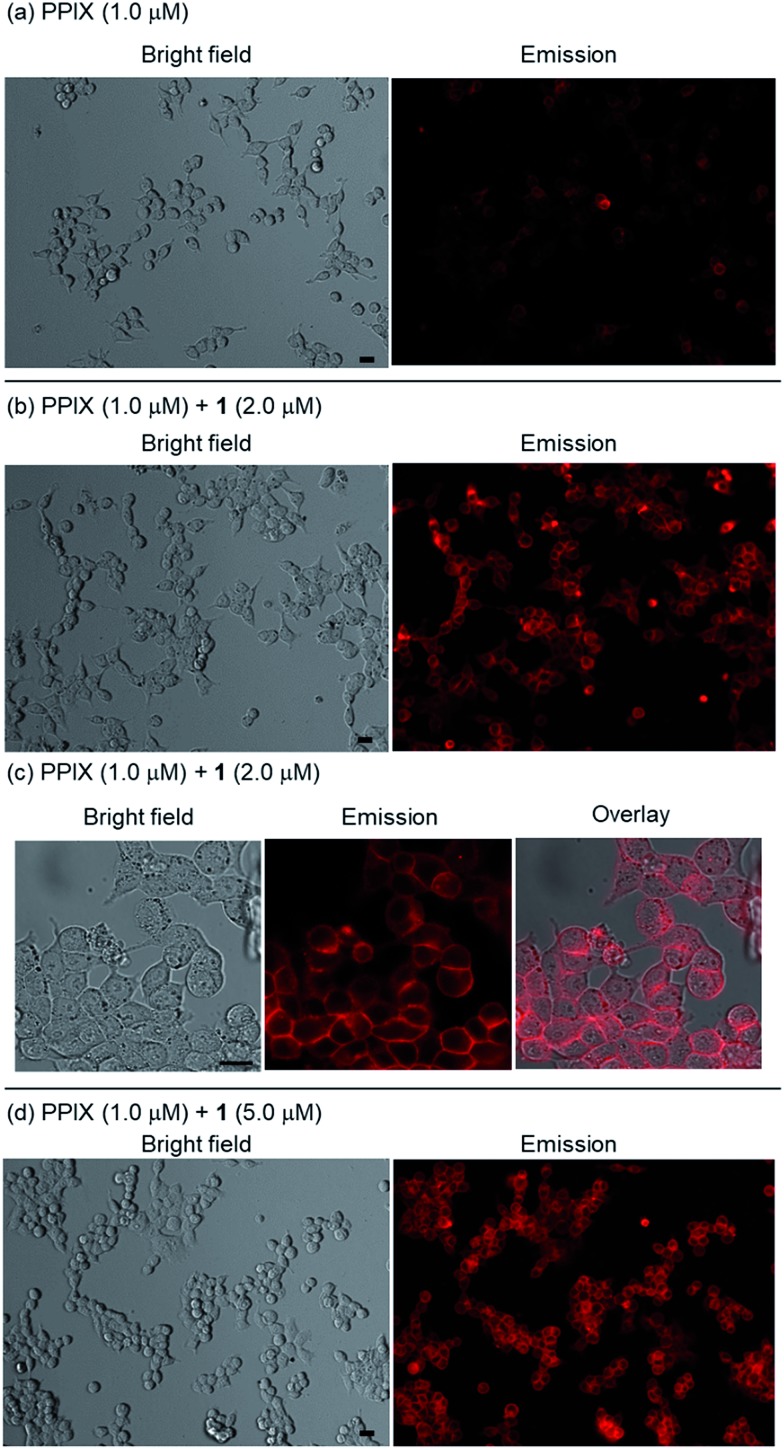
Typical fluorescence microscopy images (BZ-X710 with a TRITC filter (Ex. 545 ± 13 nm, Em. 605 ± 35 nm)) of HCT-116 cells stained with (a) PPIX (1.0 μM) alone (magnification: ×20) (b) PPIX (1.0 μM) + **1** (2.0 μM) (magnification: ×20), (c) bright field, emission and overlay images of HCT-116 cells stained with PPIX (1.0 μM) + **1** (2.0 μM) (magnification: ×40), and (d) PPIX (1.0 μM) + **1** (5.0 μM) (magnification: ×20). Scale bar (black) = 20 μm.

As shown in images (magnification: ×20) in Fig. 8a, a weak red-colored emission from PPIX in the absence of **1** was mainly observed on the cell membrane of HCT-116 cells. In contrast, an intense red-colored emission from PPIX was detected in the presence of **1** (2.0 μM) under the same observation conditions (Fig. 8b). The bright field, emission, and their overlay images (magnification: ×40) of HCT-116 cells stained with PPIX (1.0 μM) + **1** (2.0 μM) indicate that PPIX was mainly localized on the cell membrane (Fig. 8c). As shown in Fig. 8d, cells treated with PPIX in the presence of 5.0 μM of **1** also showed an intense red-colored emission whose intensity was somewhat higher than that of PPIX with 2.0 μM of **1** (Fig. 8b and d).

PPIX is present as a negatively charged porphyrin under physiological conditions due to the ionization of the two propionic acid units. Cancer cell membranes have a net negative charge due to the elevated expression of anionic molecules such as phosphatidylserine, sialylated gangliosides, *O*-glycosylated mucins, and heparan sulfate.^93^,^94^ Therefore, interactions of anionic PPIX aggregates with negatively charged cancer cell membranes should be weak due to electrostatic repulsions,^95^–^97^ resulting in the weak emission intensity of PPIX being detected on HCT-116 cells (Fig. 8a).

However, the possibility that the aggregation of PPIX on the cell membrane caused the emission quenching of PPIX cannot be ruled out. We anticipated that if PPIX aggregates form on the cell membrane, significant increase in the emission intensity of PPIX would be observed in the cell lysate prepared by treatment with 1% Triton X-100.^98^

HCT-116 cells stained with PPIX (1.0 μM) were carefully washed and then detached from the glass bottom dish by vigorous pipetting to obtain a cell suspension. The fluorescence emission spectrum of the PPIX-stained cells exhibited an emission maximum at *ca.* 635 nm, which is *ca.* 14 nm longer than that in DMSO/DMEM = 1 : 99 (v/v) (Fig. 7b).^99^ After the same sample solution was treated with Triton X-100 (final concentration: 1%) and then incubated for 30 min at 37 °C, the increase in emission intensity of PPIX in the cell lysate was *ca.* 6-fold (Fig. S31 in the ESI†), which is considerably less than that of PPIX (*ca.* 130-fold) in DMSO/DMEM = 1 : 99 (v/v) (Fig. S28 and S31 in the ESI†). These results suggest that the PPIX aggregates are formed on the cell membrane in small or negligible amounts.^98^

On the other hand, it is anticipated that negatively charged PPIX could be transformed to a positively charged supermolecule as a result of the formation of a complex with **1**, which should mainly possess a +4 charge under physiological conditions (Fig. S2 in the ESI†). In addition, the complexation of PPIX with **1** may increase its hydrophobicity. Namely, the transformation of the physical properties of PPIX *via* the formation of the complex with **1** should improve the affinity for the negatively charged membrane of cancer cells (Fig. 8b–d).

Porphyrin derivatives function as photosensitizers to produce reactive oxygen species (ROS) such as singlet oxygen (^1^O_2_) by photoirradiation.^27^,^28^ The ROS generation ability of PPIX (0.60 μM) with **1** (1.2 μM) upon photoirradiation at 530–590 nm (20 mW cm^–2^ at 550 nm) in O_2_ saturated DMSO/33 mM HEPES buffer (pH 7.4) = 2 : 3 (v/v)^100^ was evaluated by the change in UV-Vis spectra of 1,3-diphenylisobenzofuran (DPBF). The absorbance of DPBF (10 μM)^101^–^103^ at 415 nm was decreased by the reaction with ROS generated by the photoirradiation of PPIX^104^ (Fig. S32 in the ESI†). Since the difference in the absorbance at 530–590 nm between PPIX alone and PPIX with **1** is small as shown in Fig. 1, the rates of degradation of DPBF suggest that the ability of ROS generation of PPIX in the presence of **1** is similar to that for PPIX alone.

**Fig. 9:**
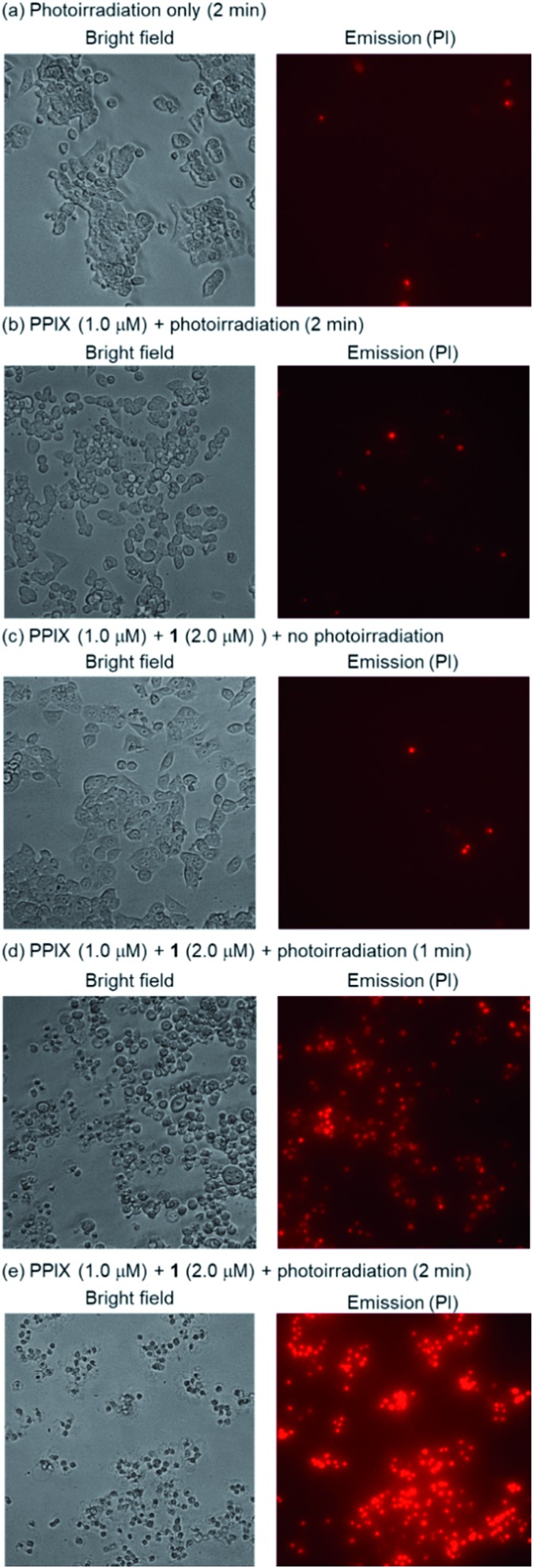
Typical fluorescence microscopy images (Biorevo BZ-9000 with a TRITC filter (Ex. 540 ± 13 nm, Em. 605 ± 28 nm)) of HCT-116 cells (magnification: ×20) after photoirradiation at 530–590 nm. Dead cells were detected by emission of PI. (a) Photoirradiation only for 2 min, (b) PPIX (1.0 μM) + photoirradiation for 2 min, (c) PPIX (1.0 μM) + **1** (2.0 μM), (d) PPIX (1.0 μM) + **1** (2.0 μM) + photoirradiation for 1 min, (e) PPIX (1.0 μM) + **1** (2.0 μM) + photoirradiation for 2 min.

**Fig. 10:**
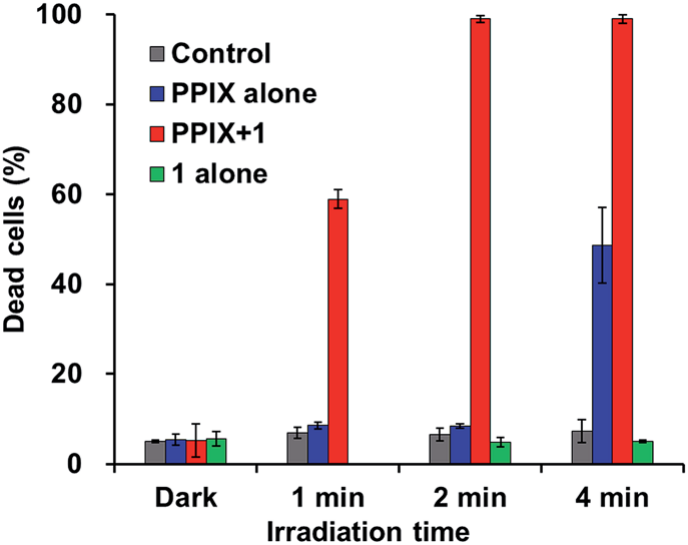
Photoinduced cell death of HCT-116 cells. The dead cells were evaluated by PI staining. The cells were treated with PPIX (1.0 μM) alone (blue bars), PPIX (1.0 μM) with **1** (2.0 μM) (red bars) or **1** (2.0 μM) alone (green bars). Untreated cells were used as control (gray bars). The data represent the mean ± standard deviation of at least triplicate experiments.

HCT-116 cells treated with PPIX (1.0 μM) in both the absence and presence of **1** (2.0 μM) for 1 h at 37 °C were photoirradiated at 530–590 nm (25 mW cm^–2^ at 550 nm) for 1–4 min.^105^ After photoirradiation, the cells were incubated for 24 h at 37 °C in a 5% CO_2_ atmosphere, and the dead cells were then detected by means of a propidium iodide (PI) treatment. PI is able to enter the dead cells and interacts with the DNA to produce a red fluorescence. Typical bright field and PI emission images and the results of PDT activity are summarized in Fig. 9, 10 and S33 in the ESI.†

As shown in Fig. 10, PPIX (1.0 μM) in the absence and the presence of **1** (2.0 μM), and **1** (2.0 μM) alone exhibited negligible cytotoxicity in the dark. It was also confirmed that photoirradiation for 4 min had a negligible effect on cell death of both untreated cells (control) and the cells treated with **1** (2.0 μM) alone (Fig. 10). Moreover, when the HCT-116 cells were treated with PPIX (1.0 μM) alone, negligible cell death of the cells was induced by photoirradiation for 2 min (Fig. 9b and 10).

In contrast, *ca.* 60% of necrotic-like cell death was observed as a result of the photoirradiation of PPIX in the presence of **1** for 1 min and almost all of the cells were dead after photoirradiation for 2 min (Fig. 9d and e, and 10). The high photocytotoxicity of PPIX in the presence of **1** suggests a positive correlation between PDT activity (Fig. 9 and 10) and the emission intensity of PPIX on the cells (Fig. 8). Although *ca.* 50% cell death was induced by PPIX alone when the photoirradiation time was extended to 4 min (Fig. 10 and S33 in the ESI†), the lowered PDT activity of PPIX could be attributed to the weak interactions between the anionic PPIX and negatively charged cell surface rather than the aggregation of PPIX on the cell membrane. These results demonstrate that the PDT activity of PPIX is improved by formation of the complex with **1**, indicating the usefulness of such supramolecular approaches based on non-covalent interactions for PDT.^96^,^97^,^106^

## Conclusions

In conclusion, we have developed a new type of 4-aminoquinoline-type molecular tweezer **1** that forms a stable complex with PPIX through multiple interactions in DMSO/33 mM HEPES buffer pH 7.4 = 2 : 3 (v/v), the *K*_11_ value of which is determined to be 4 × 10^6^ M^–1^. Furthermore, the findings show that **1** forms a more stable complex with Fe(iii)PPIX, the *K*_11_ value of which is one order of magnitude greater than that for PPIX. On the other hand, the binding constants of **1** for ZnPPIX and FMN are lower than the corresponding values for Fe(iii)PPIX and PPIX. The results of binding studies indicate that **1** could be used as a recognition unit of a synthetic heme sensor. As the next step of our study, the development of a fluorescence responsive heme sensor based on **1** is currently underway.

The formation of a stable PPIX·**1** complex (supramolecular photosensitizer) prompted us to use it for PDT. Cell staining experiments using the supramolecular photosensitizer and evaluation of its photocytotoxicity demonstrate that the PDT activity of PPIX is improved by the formation of a complex with **1**. These results indicate the advantage of using a supramolecular approach to regulating the physical properties of PPIX and improving its PDT activity *via* the use of non-covalent interactions. Further studies including the control of cellular localization and the selectivity of PPIX and other photosensitizers for cancer cells and the improvement of their PDT activities by utilizing our supramolecular system are currently underway.

## Conflicts of interest

There are no conflicts to declare.

## Supplementary Material

Supplementary information
