# Supplementary material for: Design and synthesis of a 4-aminoquinoline-based molecular tweezer that recognizes protoporphyrin IX and iron(iii) protoporphyrin IX and its application as a supramolecular photosensitizer
Source: Chem Sci. 2018 Aug 31;9(38):7455–67. doi: 10.1039/c8sc02133c (PMC6180317; doi:10.1039/c8sc02133c)
Supplement: Supplementary file 1 [file SC-009-C8SC02133C-s001.pdf]

Electronic Supplementary Material (ESI) for Chemical Science.
This journal is © The Royal Society of Chemistry 2018

Electronic Supplementary Information (ESI)

Design and Synthesis of a 4-Aminoquinoline-Based Molecular Tweezer That Recognizes Protoporphyrin IX and Iron(III) Protoporphyrin IX and Its Application as a Supramolecular Photosensitizer

Yosuke Hisamatsu[*a], Naoki Umezawa[a], Hirokazu Yagi[a], Koichi Kato[ab] and Tsunehiko Higuchi*[*a]*

[a]*Graduate School of Pharmaceutical Sciences, Nagoya City University, 3-1 Tanabe-dori, Mizuho-ku, Nagoya 467-8603, Japan.*

[b]*Exploratory Research Center on Life and Living Systems (ExCELLS) and Institute for Molecular Science (IMS), National Institutes of Natural Sciences, 5-1 Higashiyama, Myodaiji, Okazaki 444-8787, Japan*

*Corresponding authors: E-mail: hisamatsu@phar.nagoya-cu.ac.jp; higuchi@phar.nagoya-cu.ac.jp

Experimental Procedures

General Information.

All reagents and solvents were of the highest commercial quality and were used without further purification, unless otherwise noted. Dehydrated *N*,*N*-dimethylformamide (DMF), 2-(4-(4,4,5,5-tetramethyl-1,3,2-dioxaborolan-2-yl)benzyl)isoindoline-1,3-dione, palladium(II) acetate ($Pd(OAc)_2$), hydrazine monohydrate, 2,6-pyridinedicarbonyl dichloride, protoporphyrinato zinc (ZnPPIX), propidium iodide and phosphate buffered saline (PBS) were purchased from WAKO Pure Chemical Industries Ltd. S-Phos, terephthaloyl chloride, hemin chloride and riboflavin 5'-monophosphate sodium salt (flavin mononucleotide) were purchased from Tokyo Chemical Industry Co., Ltd. Anhydrous tetrahydrofuran (THF) was purchased from Kanto Chemical Co., inc. Protoporphyrin IX and 1,3-diphenylisobenzofuran (DPBF) were purchased from Sigma-Aldrich. Triton X-100 was purchased from Nacalai tesque. Thin-layer silica gel chromatography (TLC) and silica gel column chromatography were performed on Merck pre-coated plates (silica gel 60 F_{254}, 0.25 mm) and Chromatorex BW-300 (Fuji Silysia), respectively. Thin-layer NH silica gel chromatography (TLC) and NH silica gel column chromatography were performed using Chromatorex NH TLC plate (Fuji Silysia) and Chromatorex NH-DM1020 (Fuji Silysia), respectively. Buffer solutions were prepared using the Good's buffer reagents obtained from commercial sources: MES (2-morpholinoethanesulfonic acid) (Wako), HEPES (2-[4-(2-hydroxyethyl)-1-piperazinyl]ethanesulfonic acid) (Nacalai tesque), CHES (2-(cyclohexylamino)ethanesulfonic acid (Aldrich), CAPS (*N*-Cyclohexyl-3-aminopropanesulfonic acid) (Dojindo). Hazards associated with the synthetic procedures in this study were negligible.

^{1}H NMR spectra (500 MHz) were recorded on a JEOL JNM-ECZ-500R or a Varian VNMRS 500 spectrometer. ^{1}H NMR spectra (600 MHz) were recorded on a Bruker Avance600 spectrometer. Chemical shifts (δ) were determined relative to an internal reference of tetramethylsilane in $CDCl_3$ and DMSO-d_6 and solvent peak in CD_3OD. Abbreviations for multiplicity are as follows: s, singlet; d, doublet; t, triplet; q, quartet; quin, quintet; sext, sextet; m, multiplet; br, broad. ^{13}C NMR spectra (125 MHZ) were recorded on a JEOL JNM-ECZ-500R, a Varian VNMRS 500 or a Bruker Avance600

spectrometer. Chemical shifts (δ) were determined relative to solvent peaks for ^{13}C NMR spectra. The pD values in D_2O were corrected for a deuterium isotope effect using pD = [pH-meter reading] + 0.40. Electrospray ionization mass spectrometry (ESI-MS) was done with a JEOL JMS-T100LP4G. EI mass spectroscopy, high-resolution EI mass spectroscopy, FAB mass spectroscopy (JMS-SX 102A, JEOL) and elemental analyses (JM10, J-Science Lab) were carried out by the central services laboratory, Nagoya City University. Infrared (IR) spectra were recorded on a JASCO FT/IR-680 Fourier-transform infrared spectrophotometer at room temperature. Melting points were measured using an AS ONE ATM-01 and are uncorrected. UV-Vis spectra were recorded on a JASCO V-550 spectrophotometer equipped with a temperature controller (JASCO STR-458) unit at 25 ºC. Fluorescence emission spectra were recorded on a JASCO FP-8500 spectrofluorometer equipped with a temperature controller (JASCO CTU-100) unit at 25 ºC. Pure water was prepared using a Merck Millipore Elix UV 5 system. Spectrophotometric grade of DMSO (WAKO Pure Chemical Industries Ltd.) was used for the measurement of photophysical data.

Compound 7: A mixture of $Pd(OAc)_2$ (45.4 mg, 0.202 mmol), S-Phos (0.166 g, 0.404 mmol), **5** (0.616 g, 2.02 mmol), 2-(4-(4,4,5,5-tetramethyl-1,3,2-dioxaborolan-2-yl)benzyl)isoindoline-1,3-dione (0.950 g, 2.62 mmol), K_3PO_4 (1.11 g, 5.25 mmol) in degassed toluene (150 mL) and degassed H_2O (15 mL) was heated at reflux for 5 h under an Ar atmosphere and the resulting solution then cooled to room temperature. After adding $CHCl_3$ (100 mL), the mixture was washed with 1M NaOH solution. The organic layer was dried over Na_2SO_4, and then evaporated under reduced pressure. The residue was purified by silica gel column chromatography (MeOH/Et_3N = 99.5/0.5) to give **7** as a pale yellow solid (0.926 g, 91%).

M.P. 183-185 ºC (hexanes/CH_2Cl_2). IR (KBr): ν = 3240, 2963, 2936, 2803, 1766, 1713, 1583, 1569, 1391, 1346, 1336, 716 cm^{-1}. ^{1}H NMR (500 MHz, $CDCl_3$): δ = 8.54 (br d, *J* = 4.8 Hz, 1H), 8.14 (br s, 1H), 7.88-7.84 (m, 2H), 7.81-7.76 (m, 1H), 7.73-7.68 (m, 4H), 7.61 (d, *J* = 8.1 Hz, 1H), 7.55 (d, *J* = 8.2 Hz, 2H), 6.40-6.38 (m, 1H), 5.73 (br s, 1H), 4.91 (s, 2H), 3.38-3.34 (m, 2H), 2.63-2.47 (m, 6H), 1.83 (quin, *J* = 6.6 Hz, 2H), 1.73-1.63 (m, 2H), 1.04 (t, *J* = 6.6 Hz, 6H) ppm. ^{13}C NMR (125 MHz, $CDCl_3$): 168.15 (C), 151.66 (CH), 149.94 (C), 148.85 (C), 141.03 (C), 140.01 (C), 135.90 (C), 134.10 (CH), 132.20 (C), 129.29 (CH), 127.70 (CH), 127.56 (CH), 123.64 (CH), 123.48 (CH), 120.49 (CH), 118.08 (C), 98.83 (CH), 52.39

(CH_2), 46.87 (CH_2), 43.44 (CH_2), 41.42 (CH_2), 27.07 (CH_2), 25.34 (CH_2), 11.41 (CH_3) ppm. HRMS (EI) (*m/z*) Calcd for $C_{32}H_{34}N_4O_2$ [M^+]: 506.2682. Found: 506.2684.

Compound 8: A mixture of **7** (0.860 g, 1.70 mmol) and hydrazine monohydrate (0.681 g, 13.6 mmol) in ethanol (150 mL) was refluxed for 7 h under an Ar atmosphere and then cooled to room temperature. After removing the solvent, $CHCl_3$ (70 mL) was added. The organic layer was washed with 2M NaOH solution (50 mL x 2). The aqueous layer was extracted with $CHCl_3$ (50 mL). The combined organic layer was dried over Na_2SO_4 and then evaporated under reduced pressure to give **8** as a yellow gum (0.604 g, 94%). ^{1}H NMR (500 MHz, $CDCl_3$): δ = 8.55 (d, *J* = 5.7 Hz, 1H), 8.18 (d, *J* = 1.8 Hz, 1H), 7.81 (d, *J* = 8.8 Hz, 1H), 7.72 (d, *J* = 8.1 Hz, 2H), 7.67 (dd, *J* = 1.8, 8.8 Hz, 1H), 7.43 (d, *J* = 8.1 Hz, 2H), 6.40 (d, *J* = 5.7 Hz, 1H), 5.74 (br s, 1H), 3.94 (s, 2H), 3.34 (q, *J* = 6.5 Hz, 2H), 2.58 (q, *J* = 7.0 Hz, 4H), 2.51 (t, *J* = 7.1 Hz, 2H), 1.85 (quin, *J* = 7.1 Hz, 2H), 1.68 (quin, *J* = 7.1 Hz, 2H), 1.05 (t, *J* = 7.0 Hz, 6H) ppm. ^{13}C NMR (125 MHz, $CDCl_3$): 151.48 (CH), 149.97 (C), 148.80 (C), 142.83 (C), 141.18 (C), 138.82 (C), 127.64 (CH), 127.42 (CH), 127.18 (CH), 123.53 (CH), 120.58 (CH), 117.95 (CH), 98.65 (CH), 52.30 (CH_2), 46.77 (CH_2), 46.20 (CH_2), 43.32 (CH_2), 26.93 (CH_2), 25.22 (CH_2), 11.34 (CH_3) ppm. HRMS (EI) (*m/z*) Calcd for $C_{24}H_{32}N_4$ [M^+]: 376.2627. Found: 376.2612.

Compound 1: A mixture of **8** (0.523 g, 1.39 mmol), Et_3N (0.545 g, 5.38 mmol), and 2,6-pyridinedicarbonyl dichloride (0.143 g, 0.700 mmol) was stirred in dehydrated DMF (8 mL) at room temperature for 24 h under an Ar atmosphere. After removing the solvent under reduced pressure, the crude material was purified by column chromatography using Fuji Silysia Chromatorex silica gel NH ($CHCl_3$, $CHCl_3$/MeOH = 100/1 to 50/1) to give **1** (free form) (0.355 g, 57%) as a slightly cream-colored film-like amorphous solid. IR (KBr): ν = 3319, 2969, 2933, 1666, 1584, 1535, 1337, 803 cm^{-1}. ^{1}H NMR (500 MHz, $CDCl_3$): δ = 9.26 (br s, 2H), 8.49 (d, *J* = 5.4 Hz, 2H), 8.43 (d, *J* = 7.7 Hz, 2H), 8.07 (t, *J* = 7.7 Hz, 1H), 7.89 (br s, 2H), 7.80 (d, *J* = 8.9 Hz, 2H), 7.41 (br d, *J* = 8.7 Hz, 2H), 7.33 (d, *J* = 7.8 Hz, 4H), 7.08 (d, *J* = 7.8 Hz, 4H), 6.37 (d, *J* = 5.4 Hz, 2H), 6.04 (br s, 2H), 4.41 (d, *J* = 6.0 Hz, 4H), 3.32 (q, *J* = 6.6 Hz, 4H), 2.56 (q, *J* = 7.2 Hz, 8H), 2.49 (t, *J* = 7.2 Hz, 4H), 1.83 (quin, *J* = 7.0 Hz, 4H), 1.68-1.61 (m, 4H), 1.03 (t, *J* = 7.2 Hz, 12H) ppm. ^{13}C NMR (125 MHz, $CDCl_3$): δ = 164.02 (C), 151.03 (CH), 150.32 (C), 149.15 (C), 148.41 (C), 140.80 (C), 138.74 (CH), 138.09 (C), 137.87 (C), 127.66 (CH), 126.70 (CH),

126.17 (CH), 124.84 (CH), 123.48 (CH), 120.73 (CH), 117.75 (C), 98.49 (CH), 52.35 (CH_2), 46.75 (CH_2), 43.34 (CH_2), 42.48 (CH_2), 26.84 (CH_2), 25.11 (CH_2), 11.42 (CH_3) ppm. HRMS (ESI) (*m/z*) Calcd for $C_{55}H_{66}N_9O_2$ $[M+H]^+$: 884.5340. Found: 884.5302.

Compound 1·4HCl·5H₂O: Because free base of **1** is an amorphous solid, HCl salt of **1** was prepared. The mixture of **1** (free base) (35.3 mg, 39.9 μmol) in MeOH (2 mL) and 1M HCl (6 mL) was evaporated to remove MeOH and then lyophilized to give **1**· 4HCl· $5H_2O$ (41.4 mg, 93%) as a slightly cream-colored solid. M.P. 250 °C (dec.). ^{1}H NMR (500 MHz, CD_3OD): δ = 8.51 (d, *J* = 8.8 Hz, 2H), 8.40 (d, *J* = 7.1 Hz, 2H), 8.35 (d, *J* = 8.0 Hz, 2H), 8.22 (t, *J* = 7.9 Hz, 1H), 8.00 (d, *J* = 1.7 Hz, 2H), 7.98 (dd, *J* = 1.7, 8.8 Hz, 2H), 7.78 (d, *J* = 8.4 Hz, 4H), 7.57 (d, *J* = 8.4 Hz, 4H), 6.91 (d, *J* = 7.1 Hz, 2H), 4.75 (s, 4H), 3.72-3.65 (m, 4H), 3.27-3.23 (m, 12H), 1.93-1.88 (m, 8H), 1.35 (t, *J* = 7.2 Hz, 12H) ppm. ^{13}C NMR (125 MHz, CD_3OD): δ = 166.00 (C), 157.48 (C), 150.23 (C), 147.17 (C), 143.37 (CH), 141.56 (C), 140.75 (CH), 139.71 (C), 138.28 (C), 129.57 (CH), 128.61 (CH), 127.10 (CH), 126.08 (CH), 124.79 (CH), 117.87 (CH), 117.21 (C), 99.35 (CH), 52.76 (CH_2), 48.49 (CH_2), 44.05 (CH_2), 43.77 (CH_2), 26.30 (CH_2), 22.68 (CH_2), 9.20 (CH_3) ppm. HRMS (ESI) (*m/z*) Calcd for $C_{55}H_{66}N_9O_2$ $[M+H]^+$: 884.5340. Found: 884.5332. Anal. Calcd for $C_{55}H_{65}N_9O_2 \cdot 4HCl \cdot 5H_2O$: C, 58.98 H, 7.11; N, 11.25%. Found: C, 59.11; H, 7.08; N,11.36%.

Compound 2: A mixture of **8** (47.2 mg, 0.125 mmol), Et_3N (63.2 mg, 0.625 mmol), and benzoyl chloride (22.8 mg, 0.163 mmol) was stirred in anhydrous THF (1 mL) at room temperature for 14 h under an Ar atmosphere. After evaporating the solvent under reduced pressure, the residue was purified by column chromatography using Fuji Silysia Chromatorex silica gel NH ($CHCl_3$ to $CHCl_3$/MeOH = 100/1) and then recrystallized from hexanes/CH_2Cl_2 to give **2** as a slightly cream-colored solid (30.7 mg, 51%). M.P. 179-180 °C (hexanes/CH_2Cl_2). IR (KBr): ν = 3335, 2968, 2939, 1638, 1620, 1584, 1540, 1489, 1337, 1305, 798, 707 cm^{-1}. ^{1}H NMR (500 MHz, $CDCl_3$): δ = 8.56 (d, *J* = 5.3 Hz, 1H), 8.18 (d, *J* = 2.0 Hz, 1H), 7.84-7.80 (m, 3H), 7.74 (d, *J* = 8.1 Hz, 2H), 7.65 (dd, *J* = 2.1, 8.7 Hz, 1H), 7.53-7.43 (m, 5H), 6.44 (br s, 1H), 6.41 (d, *J* = 5.3 Hz, 1H), 5.80 (br s, 1H), 4.73 (d, *J* = 5.7 Hz, 2H), 3.34 (q, *J* = 6.9 Hz, 2H), 2.60 (q, *J* = 7.2 Hz, 4H), 2.53 (t, *J* = 6.9 Hz, 2H), 1.85 (quin, *J* = 6.9 Hz, 2H), 1.69 (quin, *J* = 6.9 Hz, 2H), 1.06 (t, *J* = 7.2 Hz, 6H) ppm. ^{13}C NMR (150 MHz, CD_3OD): 170.28 (C), 152.66 (C), 151.66 (CH), 149.23 (C), 143.09 (C), 140.29 (C), 140.15 (C), 135.65 (C), 132.75 (CH), 129.62 (CH), 129.22 (CH), 128.37 (CH), 126.06

(CH), 124.70 (CH), 122.96 (CH), 119.32 (C), 99.25 (CH), 53.52 (CH_2), 47.71 (CH_2), 44.23 (CH_2), 43.83 (CH_2), 27.58 (CH_2), 24.91 (CH_2), 11.14 (CH_3) ppm. HRMS (EI) (*m/z*) Calcd for $C_{31}H_{36}N_4O$ [M^+]: 480.2889. Found: 480.2895. Anal. Calcd for $C_{31}H_{36}N_4O$: C, 77.47; H, 7.55; N, 11.66%. Found: C, 77.42; H, 7.73; N, 11.57%.

Compound 3·4H₂O: A mixture of **8** (91.7 mg, 0.244 mmol), Et_3N (0.102 g, 1.00 mmol), and terephthaloyl chloride (24.7 mg, 0.122 mmol) was stirred in anhydrous DMF (1 mL) at room temperature for 17 h under an Ar atmosphere. After evaporating the solvent under reduced pressure, the residue was purified by column chromatography using Fuji Silysia Chromatorex silica gel NH ($CHCl_3$/MeOH = 60/1, 50/1, to 25/1) and then recrystallized from CH_2Cl_2/MeOH to give **3** as a colorless solid (36.9 mg, 32%). M.P. 259-261 °C (CH_2Cl_2/MeOH). IR (KBr): ν = 3339, 2968, 2934, 1637, 1585, 1541, 1338, 1375, 800 cm^{-1}. ^{1}H NMR (500 MHz, DMSO-d_6): δ = 9.20 (br t, J = 6.0 Hz, 2H), 8.34 (d, J = 5.3 Hz, 2H), 8.25 (d, J = 9.0 Hz, 2H), 7.98 (s, 4H), 7.96 (d, J = 1.8 Hz, 2H), 7.75 (d, J = 8.1 Hz, 4H), 7.68 (dd, J = 1.8, 8.8 Hz, 2H), 7.43 (d, J = 8.1 Hz, 4H), 7.18 (br t, J = 5.4 Hz, 2H), 6.40 (d, J = 5.3 Hz, 2H), 4.53 (d, J = 5.8 Hz, 4H), 3.27-3.23 (m, 4H), 2.40 (q, J = 7.1 Hz, 8H), 2.36 (t, J = 7.2 Hz, 4H), 1.64 (quin, J = 7.5 Hz, 4H), 1.47 (quin, J = 7.5 Hz, 4H), 0.90 (t, J = 7.1 Hz, 12H) ppm. ^{13}C NMR data of **3** could not be obtained due to its low solubility. HRMS (ESI) (*m/z*) Calcd for $C_{56}H_{67}N_8O_2$ [M+H]$^+$: 883.5387. Found: 883.5423. Anal. Calcd for $C_{56}H_{66}N_8O_2 \cdot 4H_2O$: C, 70.41; H, 7.81; N, 11.73%. Found: C, 70.43; H, 7.83; N, 11.79%.

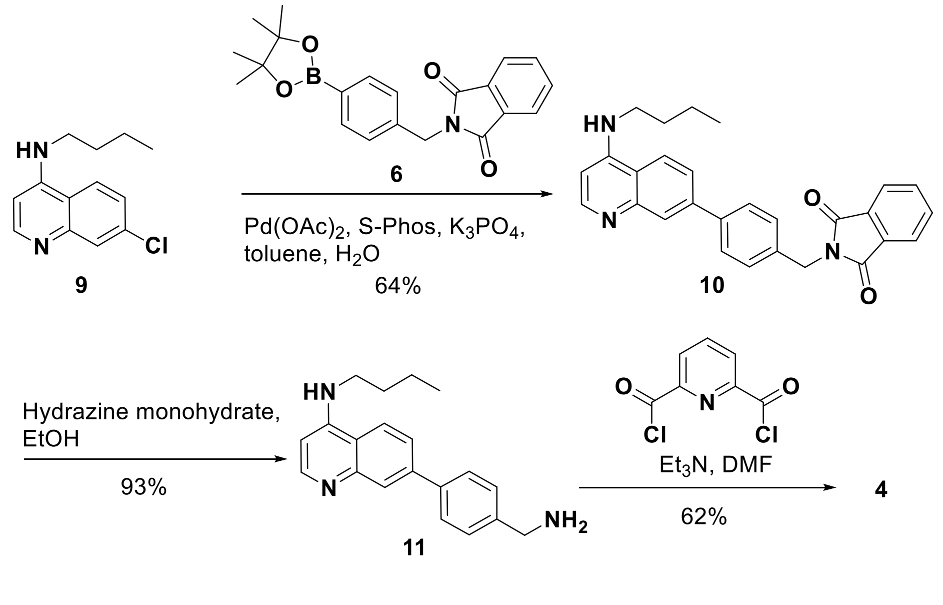

Scheme S1. Synthesis of **4**

Compound 10·0.75H$_2$O: A mixture of Pd(OAc)$_2$ (28.7 mg, 0.128 mmol), S-Phos (0.105 g, 0.256 mmol), **9**[S1] (0.300 g, 1.28 mmol), 2-(4-(4,4,5,5-tetramethyl-1,3,2-dioxaborolan-2-yl)benzyl)isoindoline-1,3-dione (0.558 g, 1.54 mmol), K_3PO_4 (0.706 g, 3.33 mmol) in degassed toluene (80 mL) and H_2O (8 mL) was heated at reflux for 13 h under an Ar atmosphere and then the cooled to room temperature. After the addition of H_2O (10 mL), the organic layer was separated. The resulting aqueous layer was extracted with $CHCl_3$ (40 mL x 2). The combined organic layers were washed with brine (50 mL x 2). The resulting organic layer was dried over Na_2SO_4, and then evaporated under reduced pressure. The residue was purified by silica gel column chromatography ($CHCl_3$/MeOH = 100/1, 50/1, to 25/1) and then recrystallized from hexanes/CH_2Cl_2 to give **10** as a pale yellow solid (0.365 g, 64%).

M.P. 217-218 °C (hexanes/CH_2Cl_2). IR (KBr): ν = 3431, 2958, 2927, 2868, 1768, 1715, 1587, 1569, 1395, 1339, 716 cm^{-1}. ^{1}H NMR (500 MHz, $CDCl_3$): δ = 8.56 (d, J = 5.3 Hz, 1H), 8.16 (d, J = 1.7 Hz, 1H), 7.88-7.84 (m, 2H), 7.77 (d, J = 8.8 Hz, 1H), 7.73-7.68 (m, 4H), 7.63 (dd, J = 1.7, 8.5 Hz, 1H), 7.55 (d, J = 8.3 Hz, 2H), 6.42 (d, J = 5.3 Hz, 1H), 5.00 (br s, 1H), 4.91 (s, 2H), 3.33 (q, J = 7.3 Hz, 2H), 1.76 (quin, J = 7.3 Hz, 2H), 1.51 (sext, J = 7.3 Hz, 2H), 1.01 (t, J = 7.3 Hz, 3H) ppm. ^{13}C NMR (125 MHz, $CDCl_3$): 168.21 (C), 151.69 (CH), 149.73 (C), 148.86 (C), 141.17 (C), 140.01 (C), 136.01 (C), 134.17 (CH), 132.27 (C), 129.36 (CH), 127.76 (CH), 123.98 (CH), 123.54 (CH), 119.97 (CH), 117.93 (C), 98.97 (CH), 43.12 (CH$_2$), 41.44 (CH$_2$), 31.17 (CH$_2$), 20.46 (CH$_2$), 14.00 (CH$_3$) ppm. ESI-MS: 436.2 [M+H]$^+$ Anal. Calcd for $C_{28}H_{25}N_3O_2$·0.75H$_2$O: C, 74.90; H, 5.95; N, 9.36%. Found: C, 74.88; H, 5.81; N, 9.30%.

Compound 11: A mixture of **10** (0.200 g, 0.459 mmol) and hydrazine monohydrate (0.206 g, 4.12 mmol) in ethanol (30 mL) was refluxed for 18 h under an Ar atmosphere and then the cooled to room temperature. After removing the solvent, $CHCl_3$ (30 mL) was added. The organic layer was washed with 5M NaOH solution (20 mL). The aqueous layer was extracted with $CHCl_3$ (30 mL x 2). The combined organic layers were dried over Na_2SO_4 and then evaporated under reduced pressure to give **11** as a yellow gum (0.130 g, 93%). ^{1}H NMR (500 MHz, $CDCl_3$): δ = 8.55 (d, J = 5.3 Hz, 1H), 8.20 (d, J = 1.7 Hz, 1H), 7.83 (d, J = 8.8 Hz, 1H), 7.65 (d, J = 8.1 Hz, 2H), 7.60 (dd, J = 1.7, 8.6 Hz, 1H), 7.37 (d, J = 8.1 Hz, 2H), 6.37 (d, J = 5.3 Hz, 1H), 5.42 (br t, J = 4.9 Hz, 1H), 3.88 (s, 2H), 3.25 (q, J = 7.4 Hz, 2H), 1.68 (quin, J = 7.4 Hz, 2H), 1.44 (sext, J = 7.4 Hz, 2H), 0.95 (t, J = 7.4 Hz, 3H) ppm. ^{13}C NMR (125 MHz, $CDCl_3$):

151.41 (CH), 149.77 (C), 148.77 (C), 142.82 (C), 141.15 (C), 138.67 (C), 127.59 (CH), 127.32 (CH), 127.16 (CH), 123.65 (CH), 120.23 (CH), 117.80 (C), 98.64 (CH), 46.12 (CH_2), 42.87 (CH_2), 30.87 (CH_2), 20.28 (CH_2), 13.82 (CH_3) ppm. HRMS (ESI) (*m/z*) Calcd for $C_{20}H_{24}N_3$ $[M+H]^+$: 306.1970. Found: 306.1951.

Compound 4·0.5H_2O: A mixture of **11** (61.9 mg, 0.203 mmol), Et_3N (0.145 g, 1.43 mmol), and 2,6-pyridinedicarbonyl dichloride (20.1 mg, 98.5 μmol) was stirred in anhydrous DMF (1 mL) at room temperature for 17 h under an Ar atmosphere. After removing the solvent under reduced pressure, the crude was purified by column chromatography using Fuji Silysia Chromatorex silica gel NH ($CHCl_3$ to $CHCl_3$/MeOH = 100/1), and then recrystallized from hexanes/CH_2Cl_2 to give **1** (46.1 mg, 62%) as a colorless solid. M.P. 282-284 °C (hexanes/CH_2Cl_2) IR (KBr): ν = 3339, 2959, 2931, 2871, 1667, 1620, 1584, 1534, 1338, 804 cm^{-1}. ^{1}H NMR (500 MHz, $CDCl_3$): δ = 9.45 (br s, 2H), 8.51 (d, *J* = 5.4 Hz, 2H), 8.45 (d, *J* = 7.9 Hz, 2H), 8.08 (t, *J* = 7.9 Hz, 1H), 7.86 (d, *J* = 1.7 Hz, 2H), 7.76 (d, *J* = 8.8 Hz, 2H), 7.38 (dd, *J* = 1.7, 8.8 Hz, 2H), 7.29 (d, *J* = 8.1 Hz, 4H), 7.02 (d, *J* = 8.1 Hz, 4H), 6.39 (d, *J* = 5.4 Hz, 2H), 5.41 (br s, 2H), 4.35 (d, *J* = 6.3 Hz, 4H), 3.33 (q, *J* = 7.3 Hz, 4H), 1.77 (quin, *J* = 7.3 Hz, 4H), 1.51 (sext, *J* = 7.3 Hz, 4H), 1.00 (t, *J* = 7.3 Hz, 6H) ppm. ^{13}C NMR (125 MHz, $CDCl_3$): δ = 163.97 (C), 151.21 (CH), 150.06 (C), 149.17 (C), 148.54 (C), 140.81 (C), 138.88 (CH), 138.25 (C), 137.87 (C), 127.75 (CH), 126.85 (CH), 126.51 (CH), 124.98 (CH), 123.74 (CH), 120.19 (CH), 117.65 (C), 98.63 (CH), 43.05 (CH_2), 42.56 (CH_2), 30.93 (CH_2), 20.38 (CH_2), 13.89 (CH_3) ppm. HRMS (ESI) (*m/z*) Calcd for $C_{47}H_{48}N_7O_2$ $[M+H]^+$: 742.3870. Found: 742.3901. Anal. Calcd for $C_{47}H_{47}N_7O_2 \cdot 0.5H_2O$: C, 75.17; H, 6.44; N, 13.06%. Found: C, 75.04; H, 6.42; N, 13.05%.

UV-Vis titrations:

Stock solutions (1.2 mM) of PPIX and ZnPPIX in DMSO were freshly prepared, respectively. Stock solution (1.2 mM) of Fe(III)PPIX in DMSO was freshly prepared by dissolving hemin chloride. Stock solution of FMN (5.0 mM) in H_2O was freshly prepared. Stock solutions of **1**·4HCl·5H_2O and **2-4** were prepared in DMSO. Titration experiments of sample solutions of PPIX (2.4 μM in DMSO/33 mM HEPES buffer = 2:3 (v/v)) in a 10 mm cuvette (3.0 mL) were performed by the addition of the stock solutions of 4-aminoquinoline compound (**1**·4HCl·5H_2O (0.24 mM), **2** (4.8 mM), **3** (1.2 mM), **4** (0.51 mM)) using a micro syringe, respectively. Titration experiments of the sample solution of Fe(III)PPIX (2.4 μM in DMSO/33 mM HEPES buffer (pH 7.4) = 2:3 (v/v)) in a 10 mm cuvette (3.0 mL) were performed by addition of the stock solutions of **1**·4HCl·5H_2O (0.24 mM) and **2** (0.24 mM) using a micro syringe, respectively. Titration experiments of the sample solution of ZnPPIX (2.4 μM in DMSO/33 mM HEPES buffer (pH 7.4) = 2:3 (v/v)) in a 10 mm cuvette (3.0 mL) were performed by the addition of the stock solution of **1**·4HCl·5H_2O (0.48 mM) using a micro syringe. The titration experiment of the sample solution of FMN (30 μM in DMSO/33 mM HEPES buffer (pH 7.4) = 2:3 (v/v)) in a 10 mm cuvette (3.0 mL) were performed by addition of the stock solution of **1**·4HCl·5H_2O (3.0 mM) using a micro syringe. Each UV-Vis spectrum in titration experiments was collected at 25 °C after stirring of sample solution for ca. 3 min. On the basis of the resulting data, the binding constants were calculated using a global curve fitting method using Bindfit program.[S2] The values of binding constants were reported as the mean±standard deviation of at least three independent experiments.

Fluorescence titrations:

Titration experiments of the sample solution of PPIX (1.0 μM in DMSO/33 mM HEPES buffer (pH 7.4) = 2:3 (v/v)) in a 10 mm cuvette (3.0 mL) were performed by the addition of stock solutions of **1**·4HCl·5H_2O (0.24 mM) using a micro syringe. Each fluorescence emission spectrum (excitation at 402 nm) in the titration experiments was collected at 25 °C after stirring of the sample solution for ca. 3 min. A.u. indicates arbitrary units. Measurement conditions of fluorescence emission spectra: excitation at 402 nm; band width (ex. 5 nm, em. 5 nm); response 0.1 sec; sensitivity: medium; wavelength scan speed: 500 nm/min. On the basis of the resulting data, the binding constants were calculated using

a global curve fitting method using the Bindfit program.[S2] Binding constants were reported as the mean±standard deviation of three independent experiments.

Calculation study:

Minimization of the PPIX·**1** complex was carried out by molecular mechanics calculation by Discovery studio 2017R2 (Biovia). The force filed used in the minimization was CHARMM force field with solvation mode (Algorithm: Adopted Basis Newton-Raphson (NR), max steps: 50000, RMS gradient: 0.01, solvation model: explicit periodic boundary, cell shape: truncated octahedron, add counter ion: sodium chloride).

Evaluation of ROS generation:

A 3.0 mL volume of a solution of DMSO/33 mM HEPES buffer (pH 7.4) = 2:3 (v/v) was bubbled with O_2 for 10 min, to which stock solutions of 1.2 mM of PPIX (1.5 μL) and 0.24 mM of **1**·4HCl·5H_2O (15 μL) were added. After measuring UV-Vis spectra of sample solutions as a base line, 15 mM of 1,3-diphenylisobenzofuran (DPBF) in DMSO (2.0 μL) was added. The resulting three sample solutions were DPBF alone (10 μM), DPBF (10 μM) + PPIX (0.60 μM), and DPBF (10 μM) + PPIX (0.60 μM) + **1** (1.2 μM) in DMSO/33 mM HEPES buffer (pH 7.4) = 2:3 (v/v). The sample solutions were photoirradiated at 530-590 nm (20 mW/cm^2 at 550 nm) using xenon light source (Max302, Asahi Spectra) at 25 °C. The remaining amount of DPBF was evaluated from the change in absorbance of DPBF at 415 nm.

UV-Vis and fluorescence titrations in DMSO/DMEM = 1:99 (v/v):

To the sample solution of **1** (0-5.0 μM) in DMSO/DMEM (high glucose, HEPES, no phenol red) (Gibco, 21063-029) = 0.8:99.2 (v/v) (2.994 mL), 6.0 μL of PPIX (0.50 mM in DMSO) was added. DMEM contains 1% penicillin/streptomycin (Gibco) and 0.1% kanamycin (Sigma). The prepared sample solutions were PPIX alone (1.0 μM) and PPIX (1.0 μM) in the presence of **1** (1.0-5.0 equiv.) in DMSO/DMEM = 1:99 (v/v) (3.0 mL). UV-vis spectra and fluorescence emission spectra of the sample solutions were measured at 25 °C. Measurement conditions of fluorescence emission spectra: excitation at 545 nm; band width (ex. 5 nm, em. 5 nm); response 0.1 sec; sensitivity: medium; wavelength scan

speed: 500 nm/min. A.u. indicates arbitrary units. Changes in the mean emission intensity at 635 nm of PPIX were expressed as the mean±standard deviation of three independent experiments.

UV-Vis and fluorescence emission spectra containing 1% Triton X-100:

The solution of PPIX (1.0 μM) in DMSO/DMEM = 1:99 (v/v) or DMSO/33 mM HEPES buffer (pH 7.4) = 2:3 (v/v) containing Triton X-100 ((final concentration: 1%) was incubated for 30 min at 37 °C. After incubating the sample solution, UV-Vis and fluorescence emission spectra were measured. Measurement conditions of fluorescence emission spectra: excitation at 545 nm; band width (ex. 5 nm, em. 5 nm); response 0.1 sec; sensitivity: medium; wavelength scan speed: 500 nm/min. The fluorescence emission spectra were corrected by background subtraction. A.u. indicates arbitrary units.

Cell culture:

HCT-116 cells were grown in DMEM (high glucose, phenol red, pyruvate, GlutaMAX™) (Gibco, 10569-010) supplemented with 10% FBS (fetal bovine serum), 1% penicillin/streptomycin (Gibco) and 0.1% kanamycin (Sigma) at 37 °C under a 5% CO_2 atmosphere.

Fluorescence imaging of HCT-116 cells:

DMEM used in this study contains 1% penicillin/streptomycin (Gibco) and 0.1% kanamycin (Sigma). HCT-116 cells (4.0×10^5 cells/mL, 300 μL) in DMEM (high glucose, phenol red, GlutaMAX™) (Gibco, 10566-016) supplemented with 10% FBS (fetal bovine serum) were seeded on each compartment of Greiner CELLview™ glass bottom petri dish (627870) (35 × 10 mm, four compartments) and incubated overnight at 37 °C under a 5% CO_2 atmosphere to allow the cells to adhere. After preparing solutions in the absence and presence of **1** (2.0 μM or 5.0 μM) in FBS free DMEM (high glucose, HEPES, no phenol red) (Gibco, 21063-029) (0.995 mL) containing 0.4% DMSO, to which 0.20 mM of PPIX in DMSO (5.0 μL) was added. The resulting sample solutions were PPIX alone (1.0 μM), PPIX (1.0 μM) + **1** (2.0 μM), and PPIX (1.0 μM) + **1** (5.0 μM) in DMSO/DMEM = 1:99 (v/v) (1.0 mL). The blank solution in DMSO/DMEM = 1:99 (v/v) (1.0 mL) was also prepared as a control.

The cells on the glass bottom petri dish were washed with fresh FBS free DMEM (high glucose, HEPES, no phenol red) (Gibco, 21063-029). After 300 μL of sample solutions of PPIX alone, PPIX+**1**, and blank were added to each compartment of the glass bottom petri dish, the cells were incubated for 1 h at 37 °C

under a 5% CO_2 atmosphere. After incubation, the medium was removed and the cells were washed twice with cold PBS (phosphate buffered saline) (Wako, 166-23555). Finally, 0.30 mL of cold PBS was added, and the cells were then observed on fluorescence microscopy (BZ-X710; Keyence, Osaka, Japan) using TRITC filter (Ex. 545±13 nm, Em. 605±35 nm).

Fluorescence emission spectra of cell suspension of HCT-116 cells:

The staining of HCT-116 cells with PPIX (1.0 μM) on a Greiner CELLview™ glass bottom petri dish (627870) (35 × 10 mm, four compartments) was carried out by using a procedure that is used for the fluorescence imaging of HCT-116 cells. After staining, the cells on the glass bottom petri dish were washed with fresh FBS free DMEM (high glucose, HEPES, no phenol red) (Gibco, 21063-029) three times. Finally, 0.60 mL of free DMEM was added to each compartment, and the cells in two compartments were then collected by vigorous pipetting to obtain a cell suspension (ca. 1.2 mL: 0.60 mL × two compartments). The fluorescence emission spectrum of the cell suspension stained with PPIX was measured at 25 ºC. The same cell suspension was treated with Triton X-100 (final concentration: 1%) and then incubated at 37 ºC for 30 min. After the incubation, the fluorescence emission spectrum of the cell lysate of HCT-116 cells containing 1% Triton X-100 was measured at 25 ºC under the same measurement conditions. Measurement conditions of fluorescence emission spectra: excitation at 545 nm; band width (ex. 10 nm, em. 10 nm; response 0.2 sec; sensitivity: medium; wavelength scan speed: 500 nm/min). The fluorescence emission spectra were corrected by background subtraction. A.u. indicates arbitrary units. Mean emission intensity was expressed as the mean±standard deviation of triplicate experiments.

Photoinduced cell death and PI staining.

HCT-116 cells (4.0×10^5 cells/mL, 100 μL) in 10% FBS DMEM (high glucose, GlutaMAX™) (Gibco 10566-016) were seeded in a 96 well plate and incubated overnight at 37 °C under a 5% CO_2 atmosphere to allow the cells to adhere. After the cells were washed with fresh FBS free DMEM (high glucose, HEPES, no phenol red) (Gibco, 21063-029) twice, the sample solutions of PPIX alone, PPIX+**1**, and blank in DMSO/DMEM = 1:99 (v/v) (100 μL) were added to each well. The sample solutions were prepared by using a procedure similar to that for the fluorescence imaging experiments. After incubation for 1 h

at 37 °C under a 5% CO_2 atmosphere, the cells were washed with fresh FBS free DMEM twice and total volume of DMEM in each well was adjusted to ca. 70 μL. The cells were photoirradiated at 530-590 nm (25 mW/cm^2 at 550 nm) using a xenon light source (Max302 or 303, Asahi Spectra) at 25 ºC, and 180 μL of DMEM (Gibco, 21063-029) supplemented with 10% FBS was then added to each well (total volume of DMEM is 250 μL) (final concentration of FBS is 7%), and then incubated for 24 h at 37 °C under a 5% CO_2 atmosphere. Subsequently, 20 μL of PI (0.18 mM) (final concentration: ca. 13 μM) was added, and the solutions were incubated for ca. 30 min at 25 ºC. Emission of PI stained cells were observed on fluorescence microscopy (Biorevo BZ-9000; Keyence, Osaka, Japan) using TRITC filter (Ex. 540±13 nm, Em. 605±28 nm). The experiments were carried out at least triplicate. For each well, 360 cells of HCT-116 cells selected from three different areas of bright field images at random, are defined as total number of selected cells. Dead cells (PI stained cells) (%) = (number of PI stained cells in the selected cells/total number of selected cells) x 100. The data represent as the mean ± standard deviation (S.D.) of at least triplicate experiments.

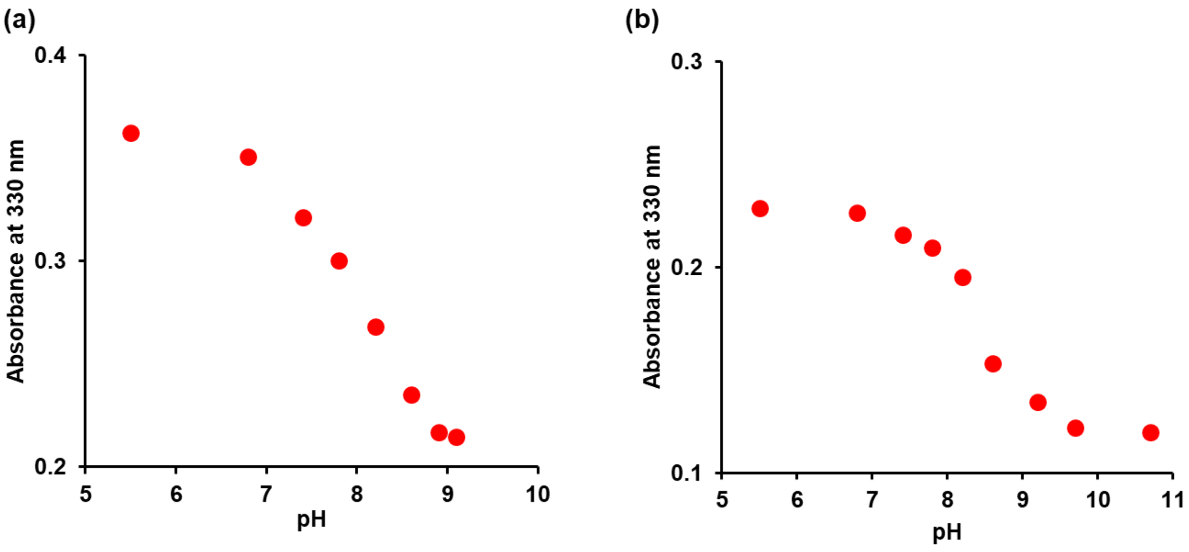

Figure S1. pH-Dependent absorbance (330 nm) change of (a) **1** and (b) **2** in DMSO/100 mM buffer = 1:4 (v/v) at 25 °C. [Compound] = 12 μM, MES buffer (pH 5.5 and 6.8), HEPES buffer (pH 7.4, 7.8 and 8.2), CHES buffer (pH 8.6, 8.9, 9.1 and 9.2), CAPS buffer (pH 9.7 and 10.7).

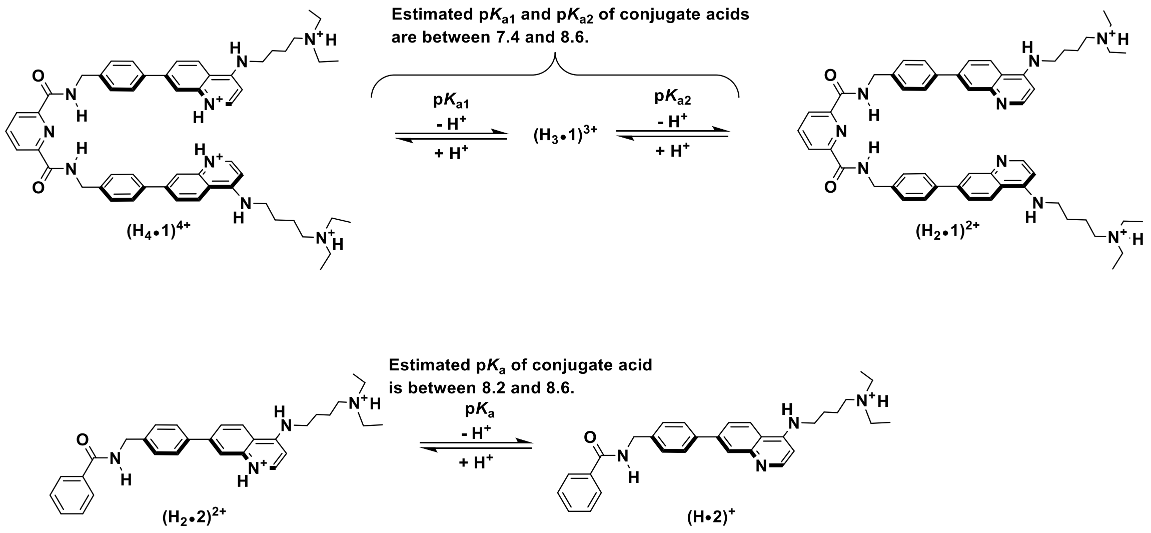

Figure S2. Estimated pK_a values of conjugate acids of nitrogen atoms on quinoline rings of **1** and **2**.

(a)

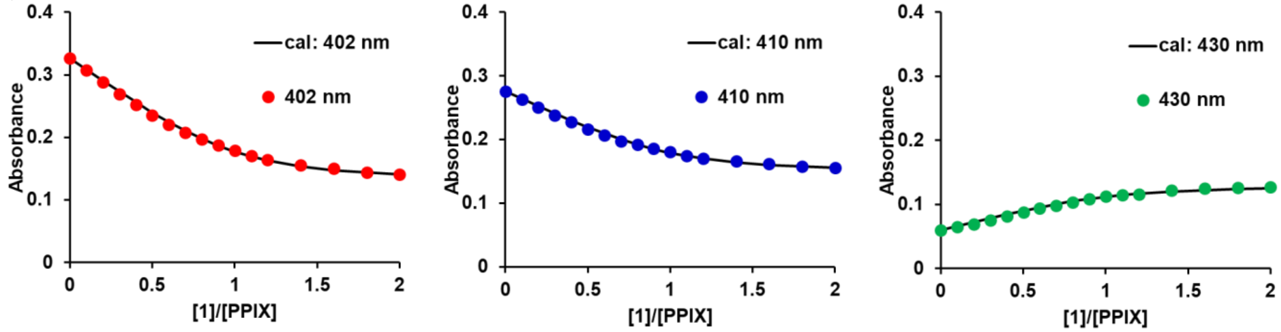

(b) Summary

Exp.	K_{11}/M$^{-1\ a}$	URL
Entry 1	4.6 × 10^6 (Error: ± 5.4%)	http://app.supramolecular.org/bindfit/view/4b210d36-a2dd-46f1-8249-ee12a1d4d453
Entry 2	4.6 × 10^6 (Error: ± 4.4%)	http://app.supramolecular.org/bindfit/view/1ec28a38-6f8a-4e25-8c85-84da03bfe641
Entry 3	2.8 × 10^6 (Error: ± 3.9%)	http://app.supramolecular.org/bindfit/view/848a9914-8669-43d8-83f7-26b29b65fca7
Mean	4.0 × 10^6	
Standard deviation	1.0 × 10^6	

[a] Calculated by 1:1 fitting model using the changes in absorbance at 402 nm, 410 nm, and 430 nm

Figure S3. UV-Vis titration of PPIX with **1** in DMSO/33 mM HEPES buffer (pH 7.4) = 2:3 (v/v) at 25 ºC. (a) Typical data of titration curves fitted to a 1:1 binding model (Nelder-Mead) using Bindfit (entry 1).[S2](b) Summary

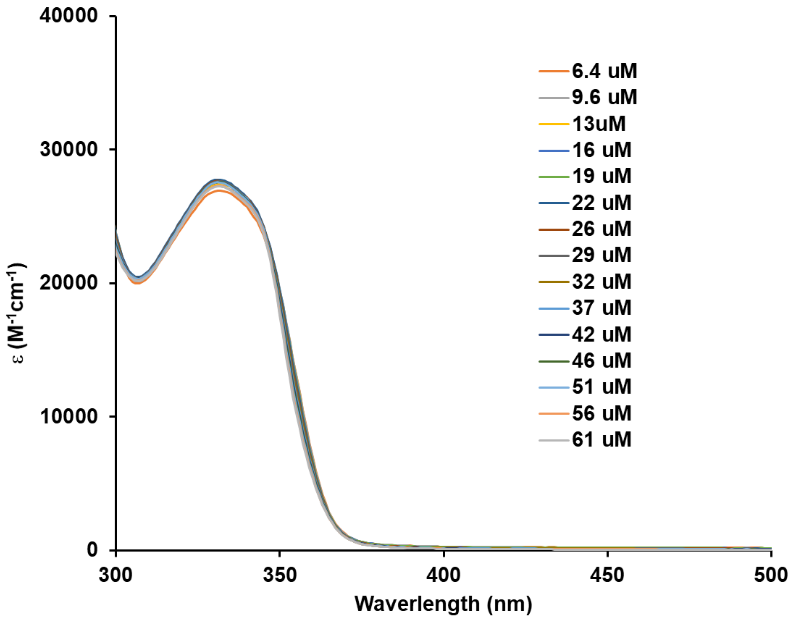

Figure S4. UV-Vis spectra of **1** at different concentrations (6.4 μM to 61 μM) in DMSO/33 mM HEPES buffer (pH 7.4) = 2:3 (v/v) at 25 ºC.

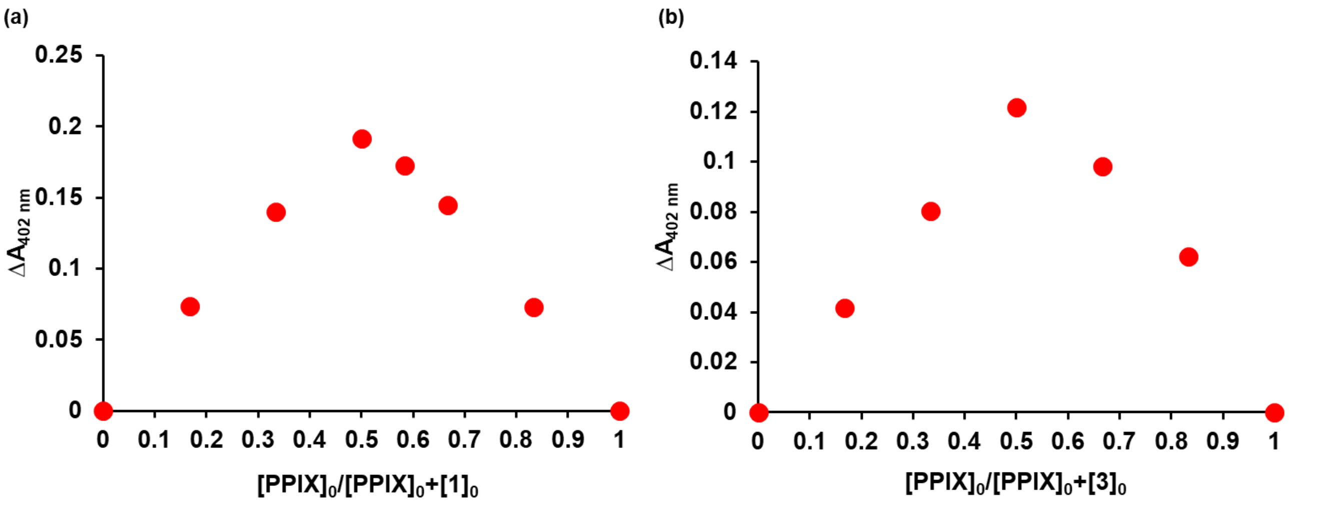

Figure S5. (a) The Job plot of PPIX with **1** in DMSO/HEPES buffer (pH 7.4) = 2:3 (v/v) at 25 ºC.[S3] Total concentration: $[PPIX]_0 + [\mathbf{1}]_0 = 6.0$ μM. (b) The Job plot of PPIX with **3** in DMSO/HEPES buffer (pH 7.4) = 2:3 (v/v) at 25 ºC. Total concentration: $[PPIX]_0 + [\mathbf{3}]_0 = 6.0$ μM. The changes in absorbance of PPIX at 402 nm were monitored.

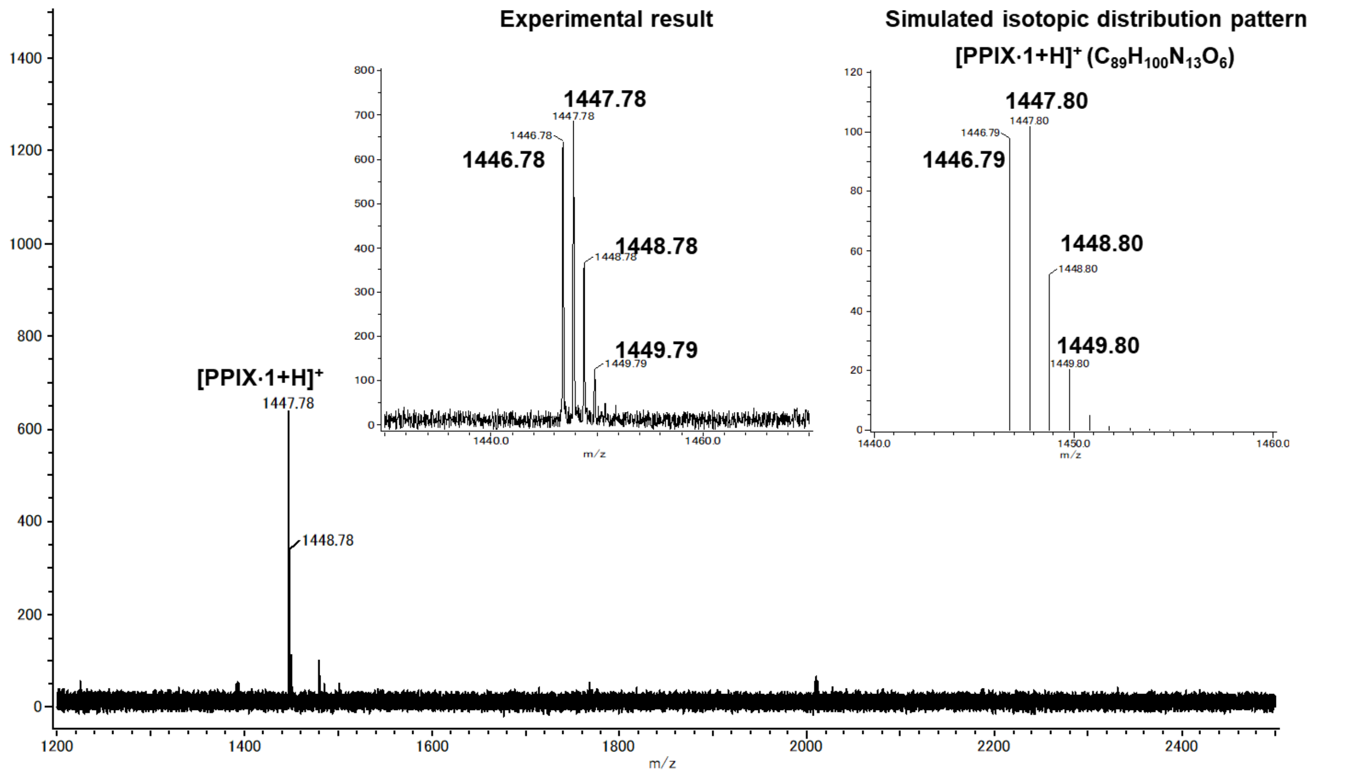

Figure S6. ESI mass spectra of PPIX·**1** complex. [**1**] = 50 μM and [PPIX] = 50 μM in DMSO/MeCN/H_2O = 0.3:2:1 (v/v/v).

(a)

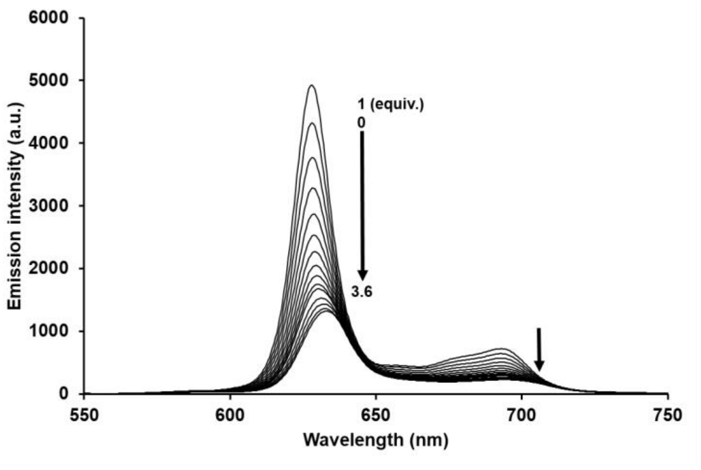

(b)

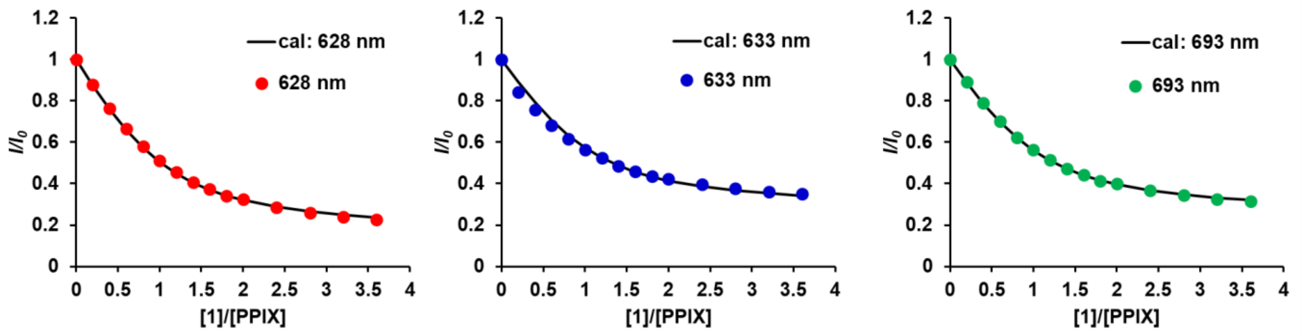

(c)

Exp.	K_{11}/M^{-1} [a]	URL
Entry 1	3.4×10^6 (Error: ±3.8%)	http://app.supramolecular.org/bindfit/view/e1e02096-49fc-439f-820d-5ebe5d35c826
Entry 2	3.9×10^6 (Error: ±7.0%)	http://app.supramolecular.org/bindfit/view/01eac2e6-6620-4f1e-b6df-87c841d28119
Entry 3	2.1×10^6 (Error: ±1.5%)	http://app.supramolecular.org/bindfit/view/c97991d6-f50c-41f1-a140-35ff20099130
Mean	3.1×10^6	
Standard deviation	0.96×10^6	

[a] Calculated by 1:1 fitting model using the changes in emission intensity at 628 nm, 633 nm, and 693 nm

Figure S7. (a) Fluorescence titration of PPIX with **1** (excitation at 402 nm) in DMSO/33 mM HEPES buffer pH 7.4 = 2:3 (v/v) at 25 °C. (b) Typical data of titration curves fitted to a 1:1 binding model (Nelder-Mead) using Bindfit (entry 1). (c) Summary

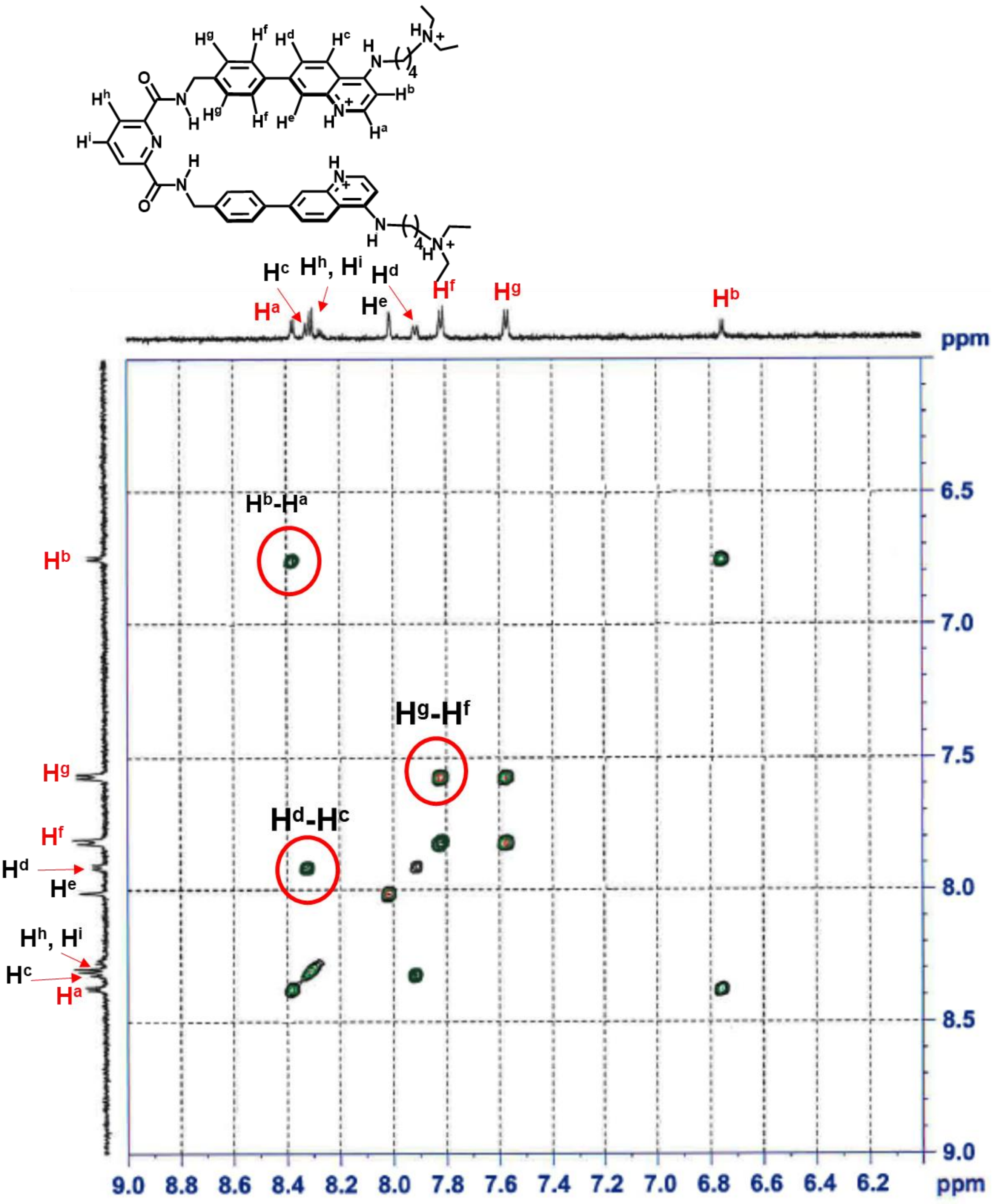

Figure S8. ^{1}H-^{1}H COSY spectrum of **1** (0.30 mM) in DMSO-d_6/33 mM HEPES buffer (pD 7.4) = 2:1 (v/v). Structure of **1** is described as tetra-protonated form.

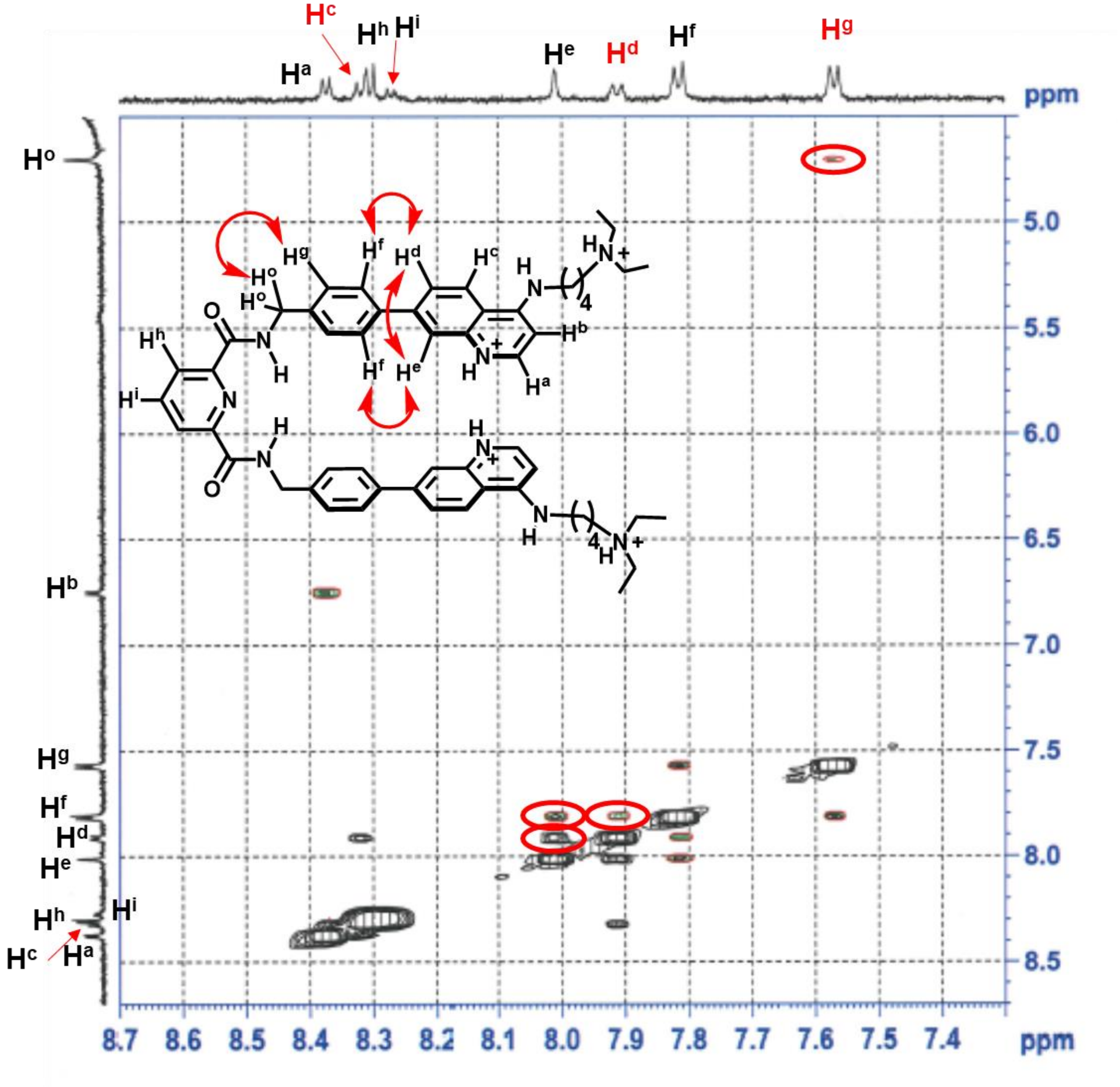

Figure S9. ROESY spectrum of **1** (0.30 mM) in DMSO-d_6/33 mM HEPES buffer (pD 7.4) = 2:1 (v/v). Structure of **1** was described as tetra-protonated form.

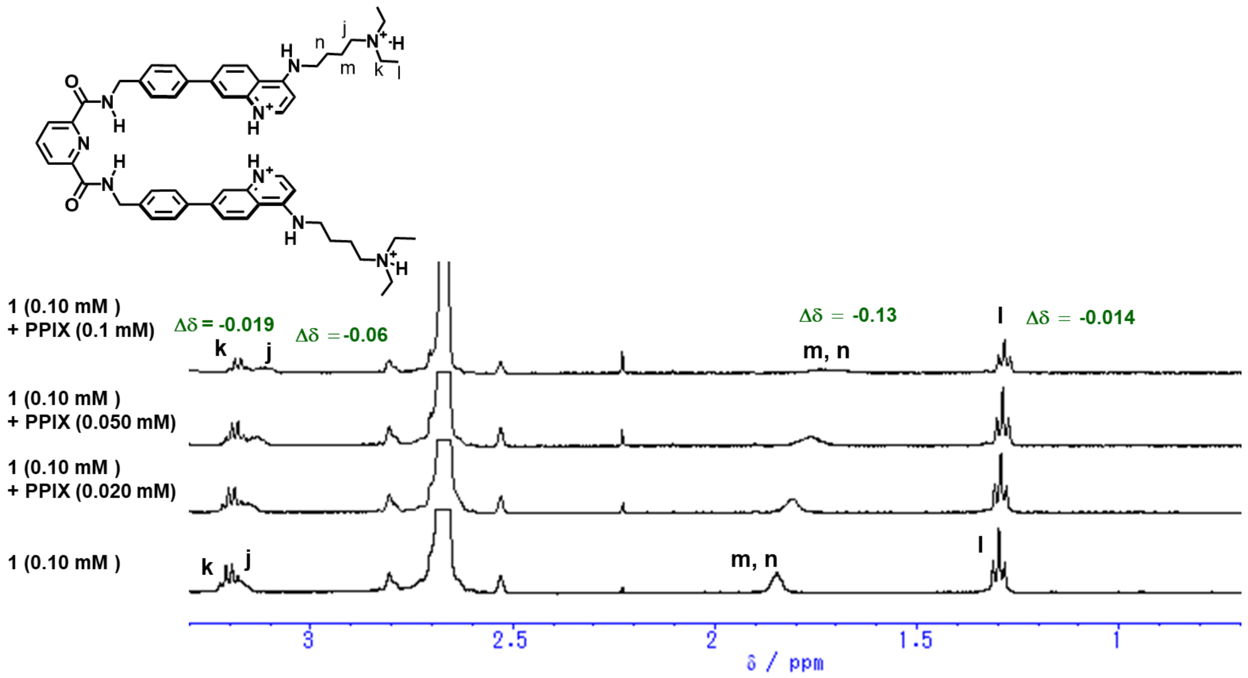

Figure S10. Partial ^{1}H NMR spectra (500 MHz) of **1** in the absence and presence of PPIX in DMSO-d_6/33 mM Tris buffer (pD 7.4) = 2:1 (v/v) at 23 °C. [**1**] = 0.10 mM, [PPIX] = 0-0.10 mM. 1% Tetramethylsilane in CCl_4 was used as external reference. Structure of **1** is described as tetra-protonated form.

Peak assignments of aliphatic protons of **1** were conducted according to the reference.[S4]

Small up-field shifts (H^j: Δδ = ca. 0.06 ppm, H^k: Δδ = ca. 0.02 ppm, H^l: Δδ = ca. 0.01 ppm) of aliphatic protons H^j, H^k and H^l located on neighbor ammonium ion upon the addition of PPIX (1.0 equiv.) were probably induced by electrostatic interactions and hydrogen bonding between ammonium ions and carboxylate ions.[S5] Moreover, the H^m and H^n proton signals exhibit upfield shifts (H^m and H^n: Δδ = ca. 0.13 ppm) to a greater extent than those for the H^{j-l} protons, suggesting that these protons were located near the π-plane of PPIX.

(a)

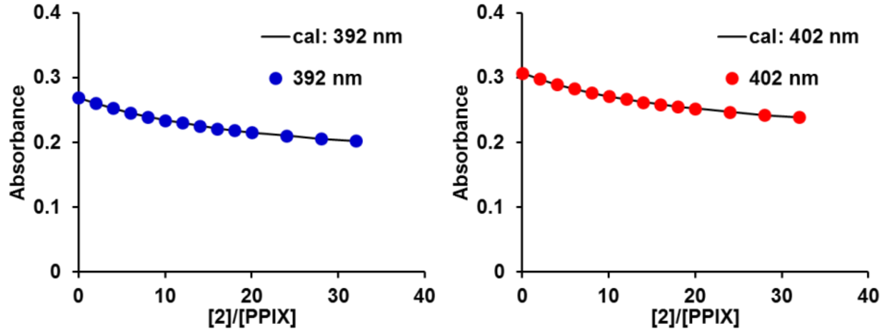

(b)

Exp.	K_{11}/M^{-1} [a]	URL
Entry 1	1.9 × 10^4 (Error: ±1.3%)	http://app.supramolecular.org/bindfit/view/46f4a2d4-8576-44e4-8f3c-661358d55beb
Entry 2	1.6 × 10^4 (Error: ±2.2%)	http://app.supramolecular.org/bindfit/view/333c6ffa-2c4f-45b7-a6c6-8f7aa9ab3ed9
Entry 3	2.3 × 10^4 (Error: ±1.4%)	http://app.supramolecular.org/bindfit/view/93d8367e-9ef7-4d26-92e6-35ed2d5b6fd1
Mean	1.9 × 10^4	
Standard deviation	0.35 × 10^4	

[a] Calculated by 1:1 fitting model using the changes in absorbance at 392 nm and 402 nm

Figure S11. UV-Vis titration of PPIX with **2** in DMSO/33 mM HEPES buffer (pH 7.4) = 2:3 (v/v) at 25 ºC. (a) Typical data of titration curves fitted to a 1:1 binding model (Nelder-Mead) using Bindfit (entry 1). (b) Summary

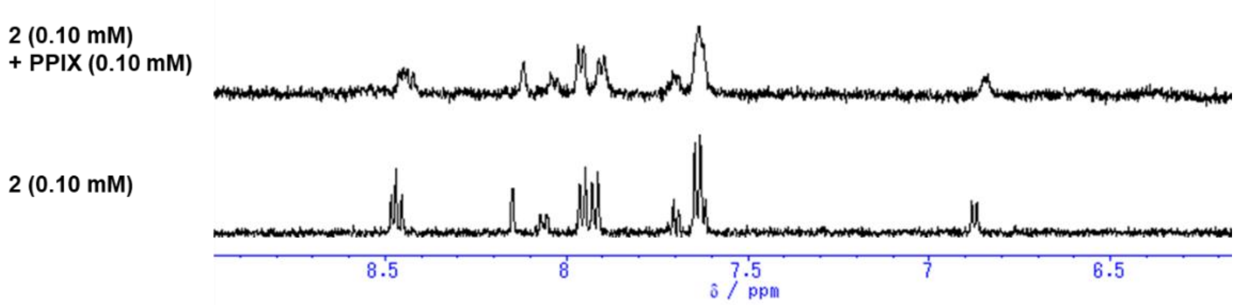

Figure S12. Partial ^{1}H NMR spectra (500 MHz) of **2** in the absence and presence of PPIX in DMSO-d_6/33 mM HEPES buffer (pD 7.4) = 2:1 (v/v) at 23 ºC. [**2**] = 0.10 mM, [PPIX] = 0 or 0.10 mM. 1% Tetramethylsilane in CCl_4 was used as external reference.

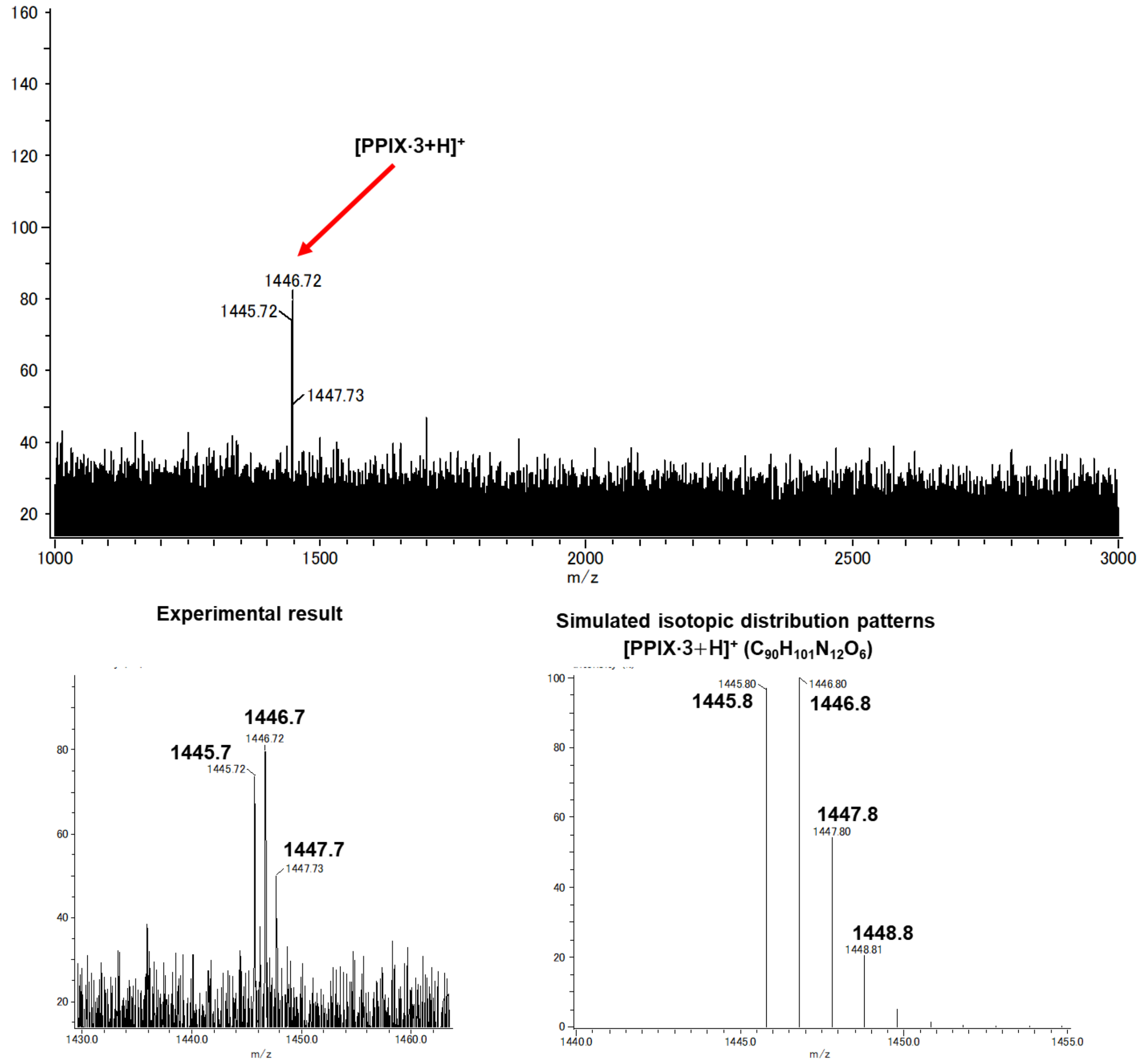

Figure S13. ESI mass spectra of PPIX·**3** complex. [**3**] = 50 μM and [PPIX] = 50 μM in DMSO/MeCN/H_2O = 0.3:2:1 (v/v/v).

(a)

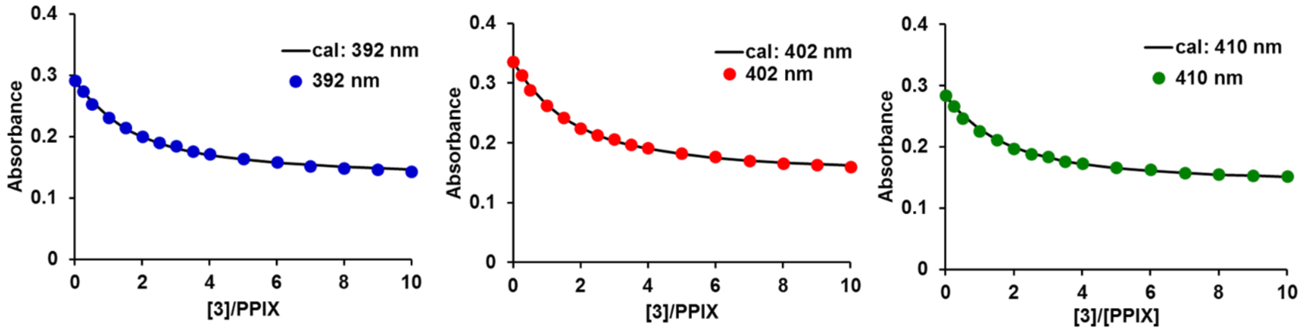

(b)

Exp.	K_{11}/M$^{-1\,a}$	URL
Entry 1	3.9 × 10^5 (Error: ±2.4%)	http://app.supramolecular.org/bindfit/view/e2e74c93-9c18-4a09-9460-2da2d8180228
Entry 2	4.0 × 10^5 (Error: ±1.6%)	http://app.supramolecular.org/bindfit/view/92b52b83-286f-4ebd-9325-8f5fccdc4212
Entry 3	4.2 × 10^5 (Error: ±2.0%)	http://app.supramolecular.org/bindfit/view/252bc284-a791-4e58-a14a-fe3325ac489c
Mean	4.0 × 10^5	
Standard deviation	0.19 × 10^5	

[a] Calculated by 1:1 fitting model using the changes in absorbance at 392 nm, 402 nm and 410 nm

Figure S14. UV-Vis titration of PPIX with **3** in DMSO/33 mM HEPES buffer (pH 7.4) = 2:3 (v/v) at 25 ºC. (a) Typical data of titration curves fitted to a 1:1 binding model (Nelder-Mead) using Bindfit (entry 1). (b) Summary

(a)

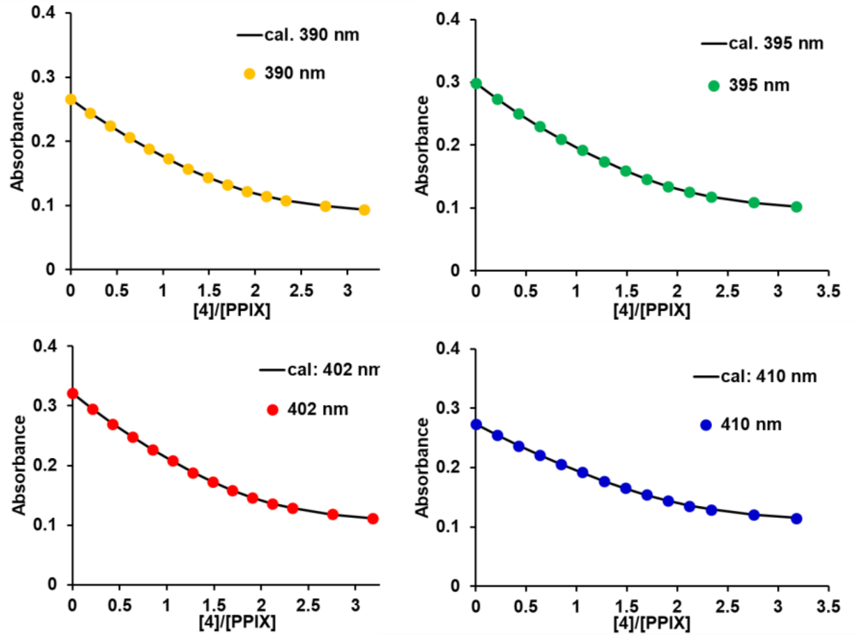

(b)

Exp.	K_{11}/M^{-1} [a]	K_{12}/M^{-1}	URL
Entry 1	1.7 × 10^6 (Error: ±2.1%)	1.7 × 10^6 (Error: ±3.2%)	http://app.supramolecular.org/bindfit/view/fd34b609-88fe-435e-86bf-64cd8fa80f78
Entry 2	0.99 × 10^6 (Error: ±4.1%)	2.8 × 10^6 (Error: ±5.1%)	http://app.supramolecular.org/bindfit/view/4ced18ed-8c43-42bf-9511-f7c2acb7ae87
Entry 3	1.7 × 10^6 (Error: ±8.1%)	2.2 × 10^6 (Error: ±6.4%)	http://app.supramolecular.org/bindfit/view/20b6a28c-740c-4a80-870d-d4ed88d0294d
Mean	1.5 × 10^6	2.3 × 10^6	
Standard deviation	0.41 × 10^6	0.56 × 10^6	

[a] Calculated by 1:2 fitting model using the changes in absorbance at 390 nm, 395 nm, 402 nm and 410 nm

Figure S15. UV-Vis titration of PPIX with **4** in DMSO/33 mM HEPES buffer (pH 7.4) = 2:3 (v/v) at 25 °C. (a) Typical data of titration curves fitted to a 1:2 binding model (Nelder-Mead) using Bindfit (entry 1). (b) Summary

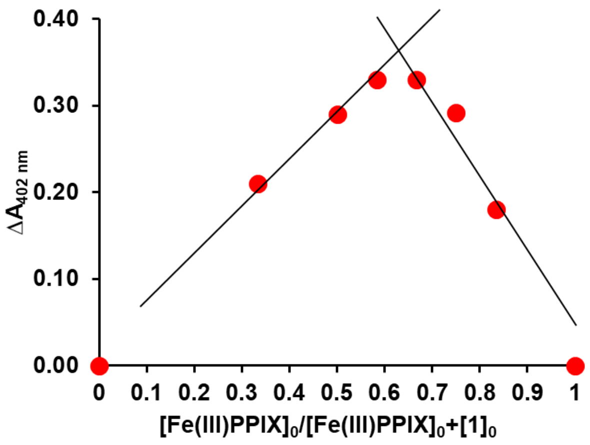

Figure S16. The Job plot of Fe(III)PPIX with **1** in DMSO/HEPES buffer (pH 7.4) = 2:3 (v/v) at 25 ºC.[S3] Total concentration: $[Fe(III)PPIX]_0$ + $[\mathbf{1}]_0$ = 6.0 μM. The changes in absorbance of Fe(III)PPIX at 402 nm were monitored.

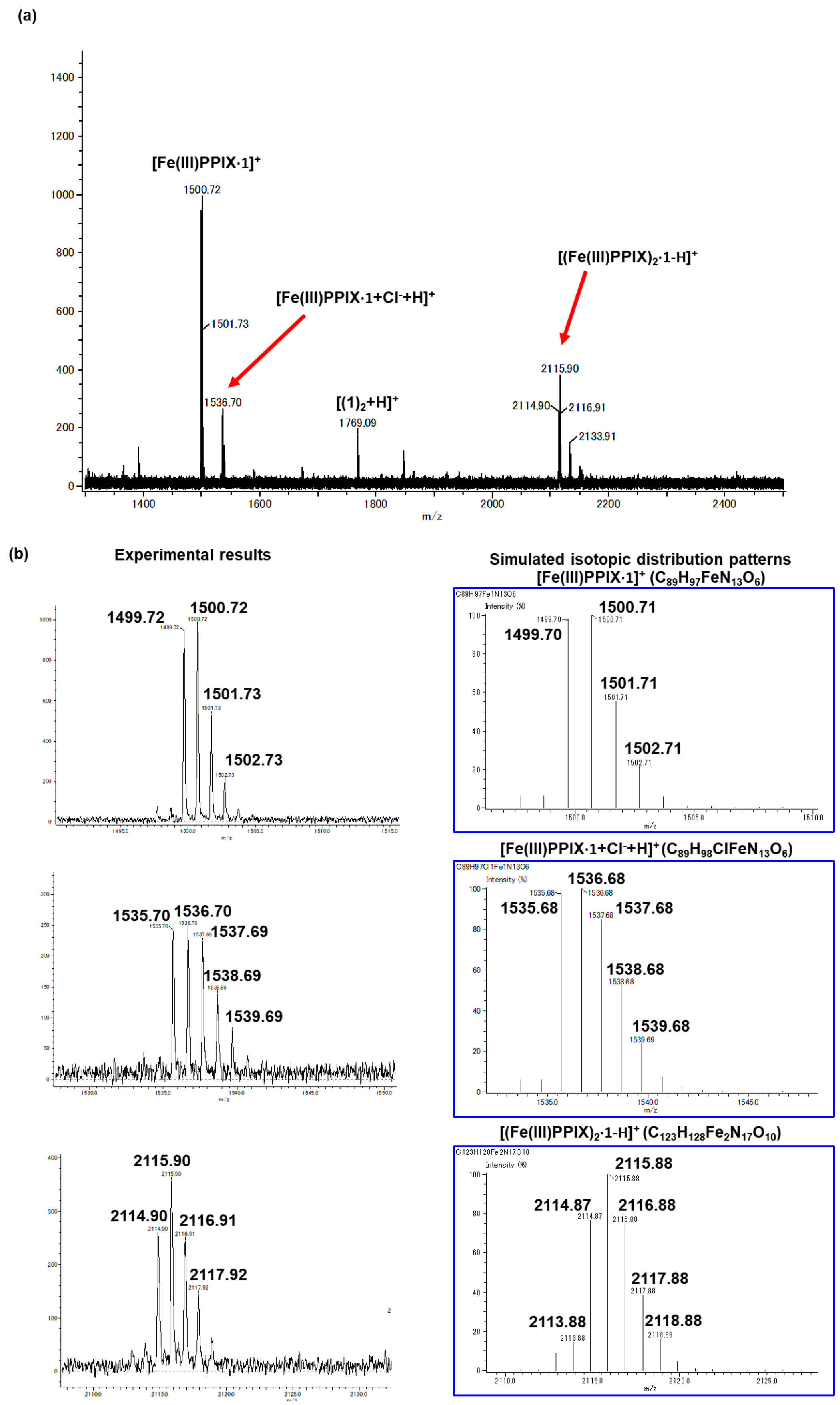

Figure S17. ESI mass spectra of Fe(III)PPIX in the presence of **1**. [**1**] = 50 μM and [Fe(III)PPIX] = 50 μM in DMSO/MeOH/H_2O = 0.2:2:1 (v/v/v)

(a)

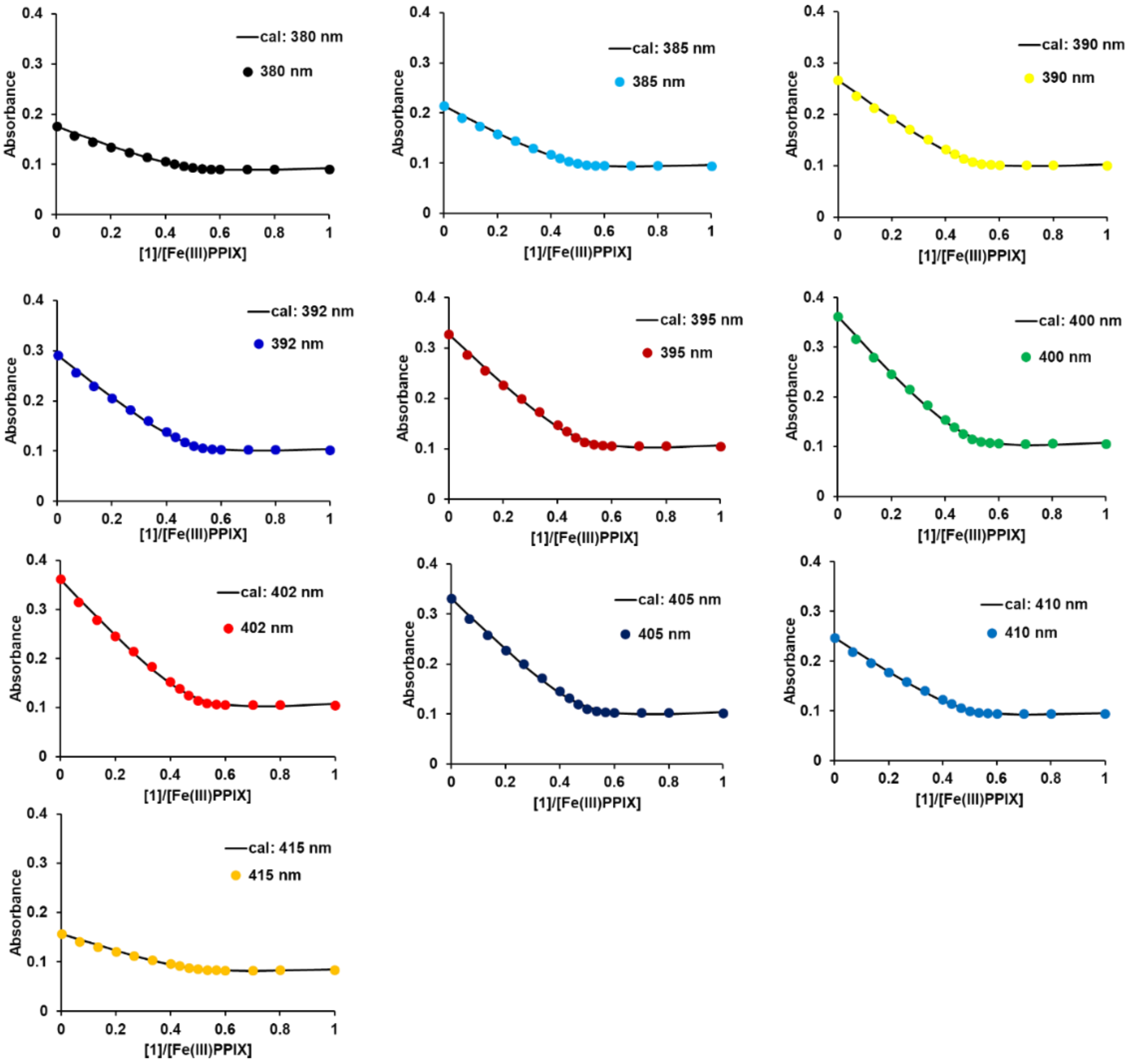

Absorbance
[1]/[Fe(III)PPIX]
cal: 380 nm
380 nm
cal: 385 nm
385 nm
cal: 390 nm
390 nm
cal: 392 nm
392 nm
cal: 395 nm
395 nm
cal: 400 nm
400 nm
cal: 402 nm
402 nm
cal: 405 nm
405 nm
cal: 410 nm
410 nm
cal: 415 nm
415 nm

(b)

Exp.	K_{11}/M$^{-1\ a}$	K_{21}/M$^{-1\ a}$	URL
Entry 1[b]	2.9 × 10^{7} (Error: ±32%)	5.8 × 10^{6} (Error: ±4.0%)	http://app.supramolecular.org/bindfit/view/ce6b2e8b-ad48-46c2-9b0b-aa6dc993335d
Entry 2[b]	2.1 × 10^{7} (Error: ±22%)	7.8 × 10^{6} (Error: ±3.1%)	http://app.supramolecular.org/bindfit/view/9406f2d7-0a2f-4fff-81ae-df4ab4667753
Entry 3[b]	4.4 × 10^{7} (Error: ±30%)	1.6 × 10^{6} (Error: ±1.9%)	http://app.supramolecular.org/bindfit/view/37d5a91d-47fd-4d5c-bd32-cd3afce4bdba
Entry 4[b]	6.9 × 10^{7} (Error: ±38%)	3.5 × 10^{6} (Error: ±2.8%)	http://app.supramolecular.org/bindfit/view/7bf9a95f-7258-4fff-812c-7055d9e0b16e
Entry 5[c]	3.2 × 10^{7} (Error: ±35%)	7.9 × 10^{6} (Error: ±5.1%)	http://app.supramolecular.org/bindfit/view/59a0fb02-ff52-4615-8e3b-d9e13118738a
Lower limit of the value	>2 × 10^{7}	>2 × 10^{6}	

[a] Calculated by 2:1 fitting model using the changes in absorbance at 380 nm, 385 nm, 390nm, 392 nm, 395 nm, 400 nm, 402 nm, 405 nm, 410 nm and 415 nm

[b][Fe(III)PPIX] = 2.4 μM

[c][Fe(III)PPIX] = 2.5 μM

Figure S18. UV-Vis titration of Fe(III)PPIX with **1** in DMSO/33 mM HEPES buffer (pH 7.4) = 2:3 (v/v) at 25 ºC. (a) Typical data of titration curves fitted to a 2:1 binding model (Nelder-Mead) using Bindfit (entry 1). (b) Summary

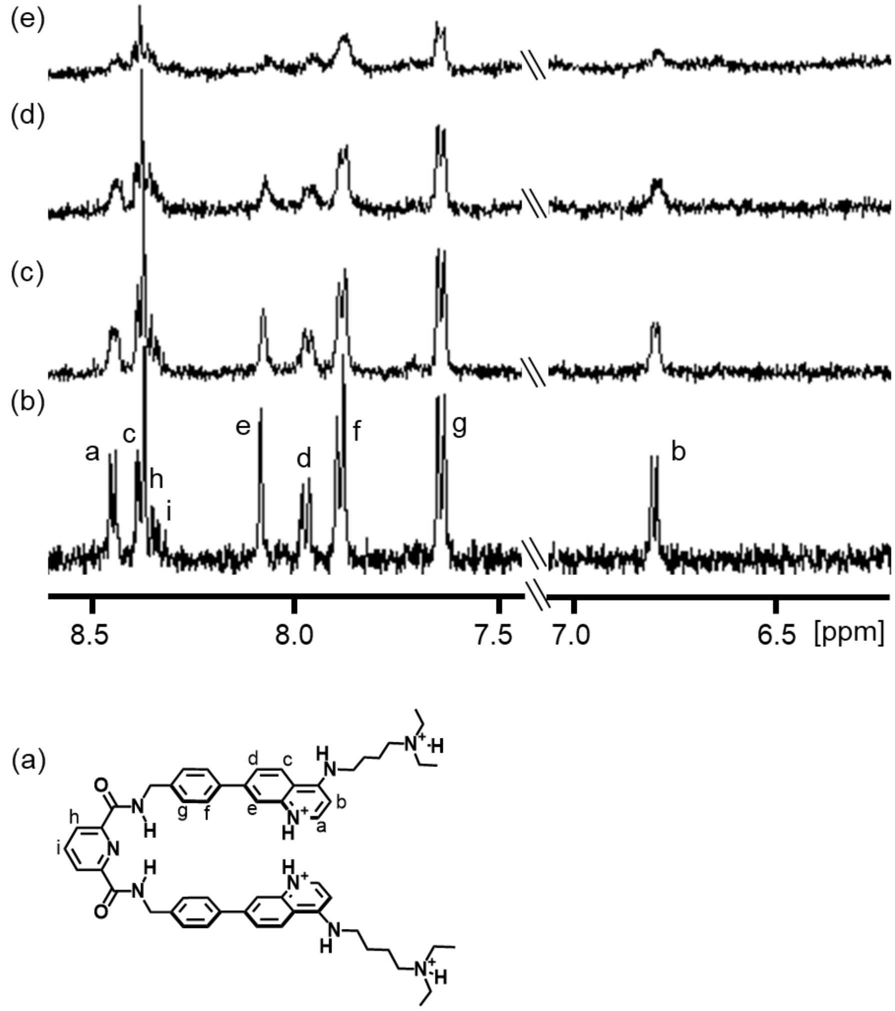

Figure S19. Partial ^{1}H NMR spectra (500 MHz) of **1** in the absence and the presence of Fe(III)PPIX in DMSO-d_6/33 mM HEPES buffer (pD 7.4) = 2:1 (v/v) at 23 ºC. (a) Structure of **1** described as tetra-protonated form. (b) **1** (0.10 mM), (c) **1** (0.10 mM) + Fe(III)PPIX (0.020 mM), (d) **1** (0.10 mM) + Fe(III)PPIX (0.050 mM) and (e) **1** (0.10 mM) + Fe(III)PPIX (0.10 mM). 1% Tetramethylsilane in CCl_4 was used as external reference.

(a) (b)

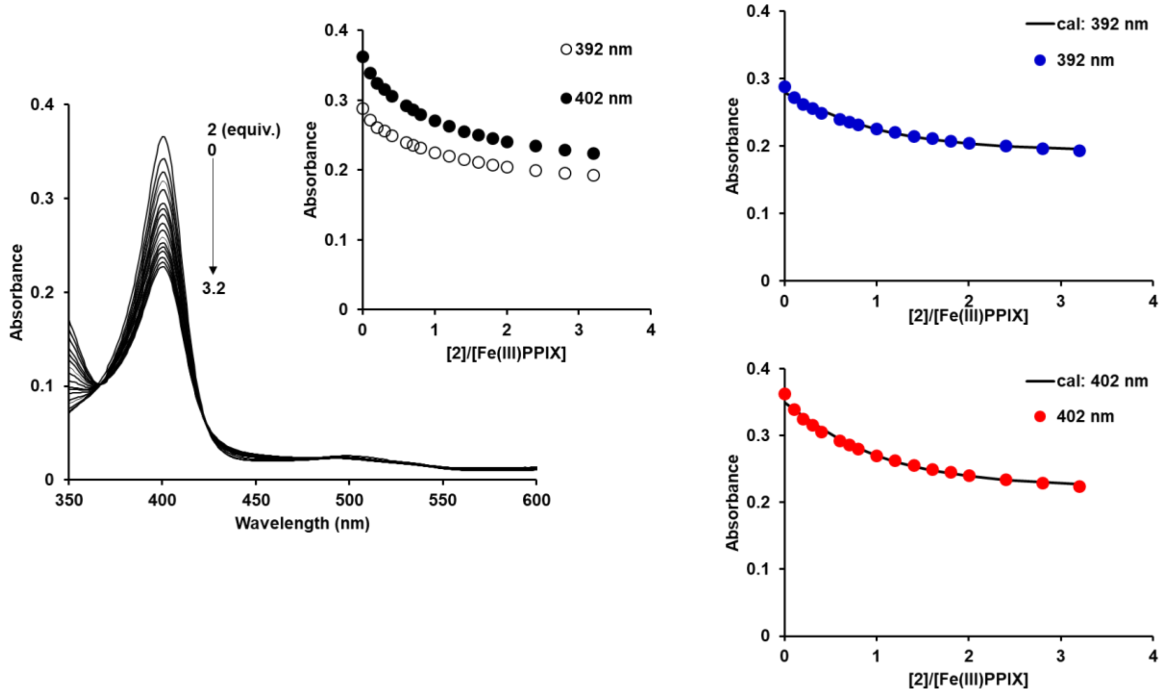

(c)

Exp.	$K_{11}/M^{-1\ a}$	URL
Entry 1	1.3×10^6 (Error: ±9.3%)	http://app.supramolecular.org/bindfit/view/0a521c98-d640-47fc-a92a-8e4533b9485f
Entry 2	1.2×10^6 (Error: ±20%)	http://app.supramolecular.org/bindfit/view/d6927b28-8696-43fe-8e5f-e81d1ac65919
Entry 3	1.6×10^6 (Error: ±11%)	http://app.supramolecular.org/bindfit/view/eae5b957-185a-4586-b3fe-17397bcac4f2
Mean	1.4×10^6	
Standard deviation	0.20×10^5	

[a] Calculated by 1:1 fitting model using the changes in absorbance at 392 nm and 402 nm

Figure S20. (a) UV-Vis titration of Fe(III)PPIX (2.4 μM) with **2** in DMSO/33 mM HEPES buffer pH 7.4 = 2:3 (v/v) at 25 ºC. (b) Typical data of titration curves fitted to a 1:1 binding model (Nelder-Mead) using Bindfit (entry 1). (c) Summary

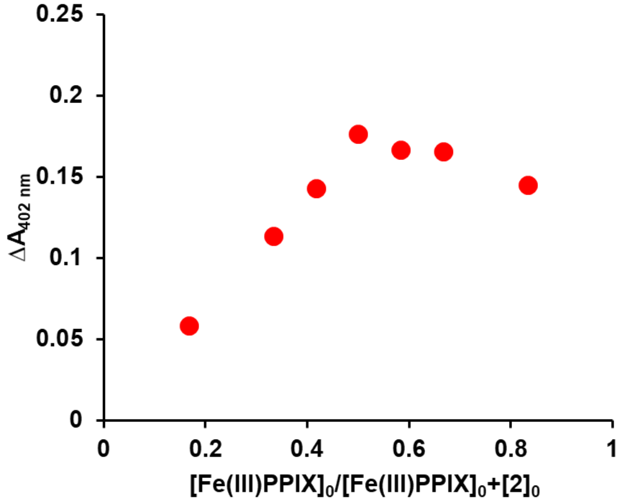

Figure S21. (a) The Job plot of Fe(III)PPIX with **2** in DMSO/33 mM HEPES buffer (pH 7.4) = 2:3 (v/v) at 25 °C.[S3] Total concentration: $[\text{Fe(III)PPIX}]_0 + [\mathbf{2}]_0 = 6.0\ \mu\text{M}$

(a)

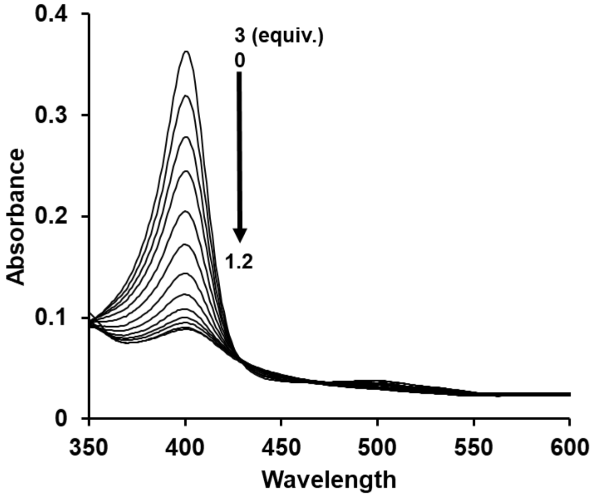

(b)

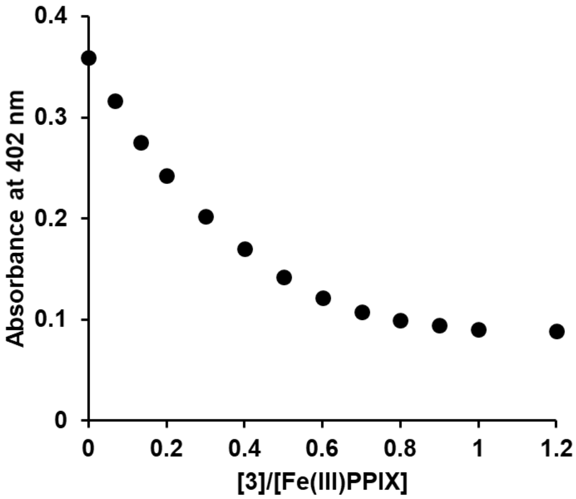

Figure S22. (a) UV-Vis titration of Fe(III)PPIX (2.4 μM) with **3** in DMSO/33 mM HEPES buffer pH 7.4 = 2:3 (v/v) at 25 °C. (b) Titration curve of Fe(III)PPIX (at 402 nm) with **3**.

(a)

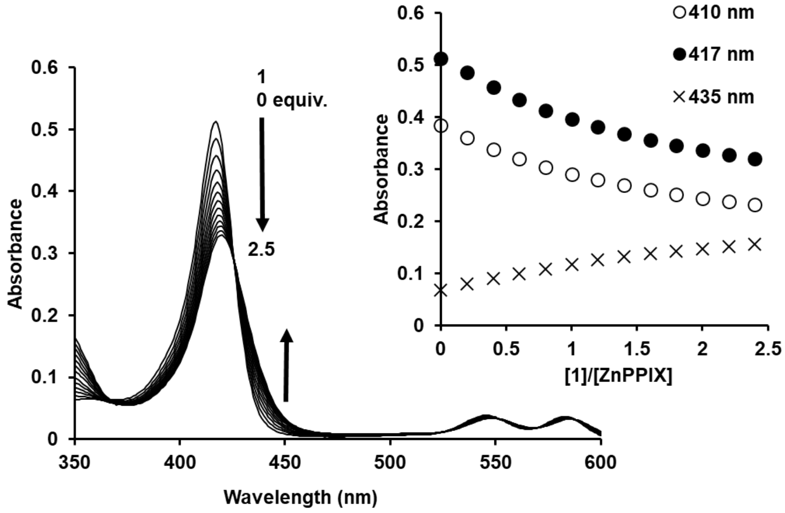

(b)

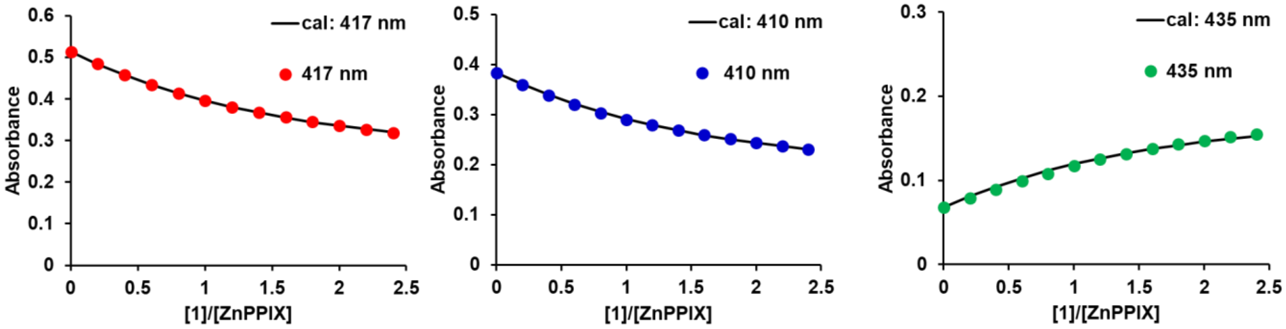

(c)

Exp.	K_{11}/M$^{-1\ a}$	URL
Entry 1	4.5×10^5 (Error: ±1.3%)	http://app.supramolecular.org/bindfit/view/29bbe08f-12c7-4a88-b2b3-fc3c3648cce4
Entry 2	5.2×10^5 (Error: ±1.4%)	http://app.supramolecular.org/bindfit/view/5af3afc0-d300-4089-9e2b-f09f860a5af8
Entry 3	5.6×10^5 (Error: ± 1.8%)	http://app.supramolecular.org/bindfit/view/c4c6d18d-1c52-4d57-bb09-904059764174
Mean	5.1×10^5	
Standard deviation	0.57×10^5	

[a] Calculated by 1:1 fitting model using the changes in absorbance at 410 nm, 417 nm and 435 nm

Figure S23. (a) UV-Vis titration of ZnPPIX (2.4 μM) with **1** in DMSO/33 mM HEPES buffer (pH 7.4) = 2:3 (v/v) at 25 °C. Inset: Changes in absorbance of ZnPPIX upon the addition of **1**. (b) Typical data of titration curves fitted to a 1:1 binding model (Nelder-Mead) using Bindfit (entry 1). (c) Summary

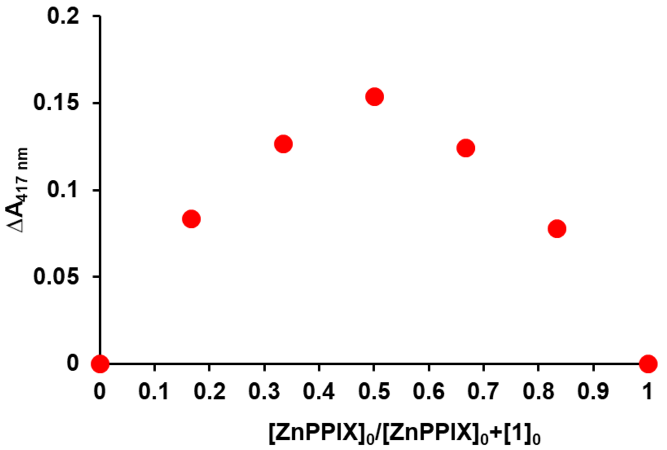

Figure S24. (a) The Job's plot of ZnPPIX with **1** in DMSO/33 mM HEPES buffer (pH 7.4) = 2:3 (v/v) at 25 ºC. $[\text{ZnPPIX}]_0 + [\mathbf{1}]_0 = 6.0\ \mu\text{M}$

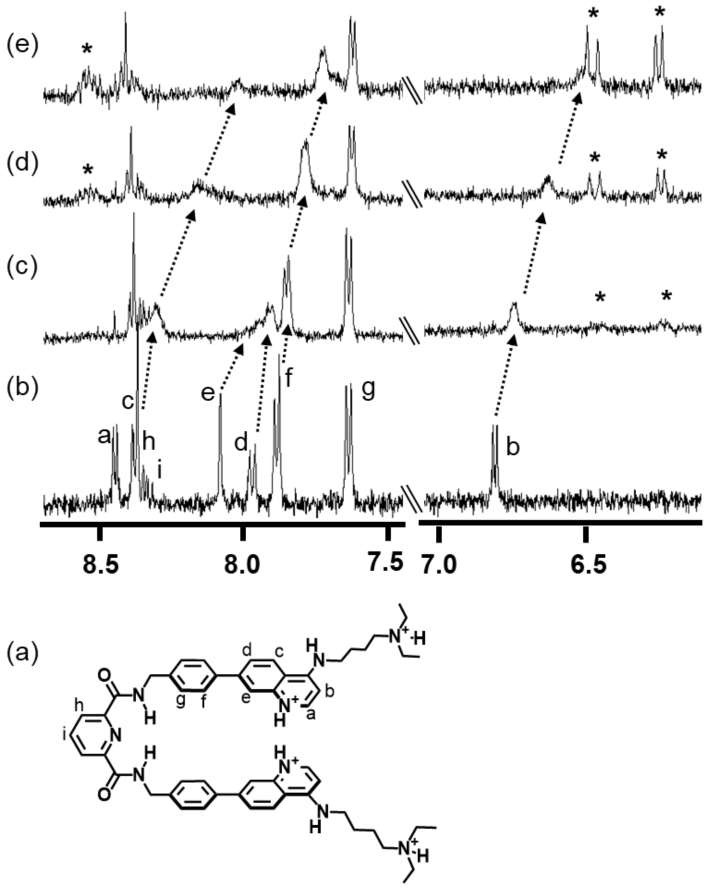

Figure S25. Partial ^{1}H NMR spectra (500 MHz) of **1** in the absence and the presence of ZnPPIX in D_2O/33 mM HEPES buffer (pD 7.4) = 2:1 (v/v) at 23 ºC. (a) Structure of **1** is described as tetra-protonated form. (b) **1** (0.10 mM), (c) **1** (0.10 mM) + ZnPPIX (0.020 mM), (d) **1** (0.10 mM) + ZnPPIX (0.050 mM), (e) **1** (0.10 mM) + ZnPPIX (0.10 mM). Asterisks are proton signals of ZnPPIX. 1% Tetramethylsilane in CCl_4 was used as external reference.

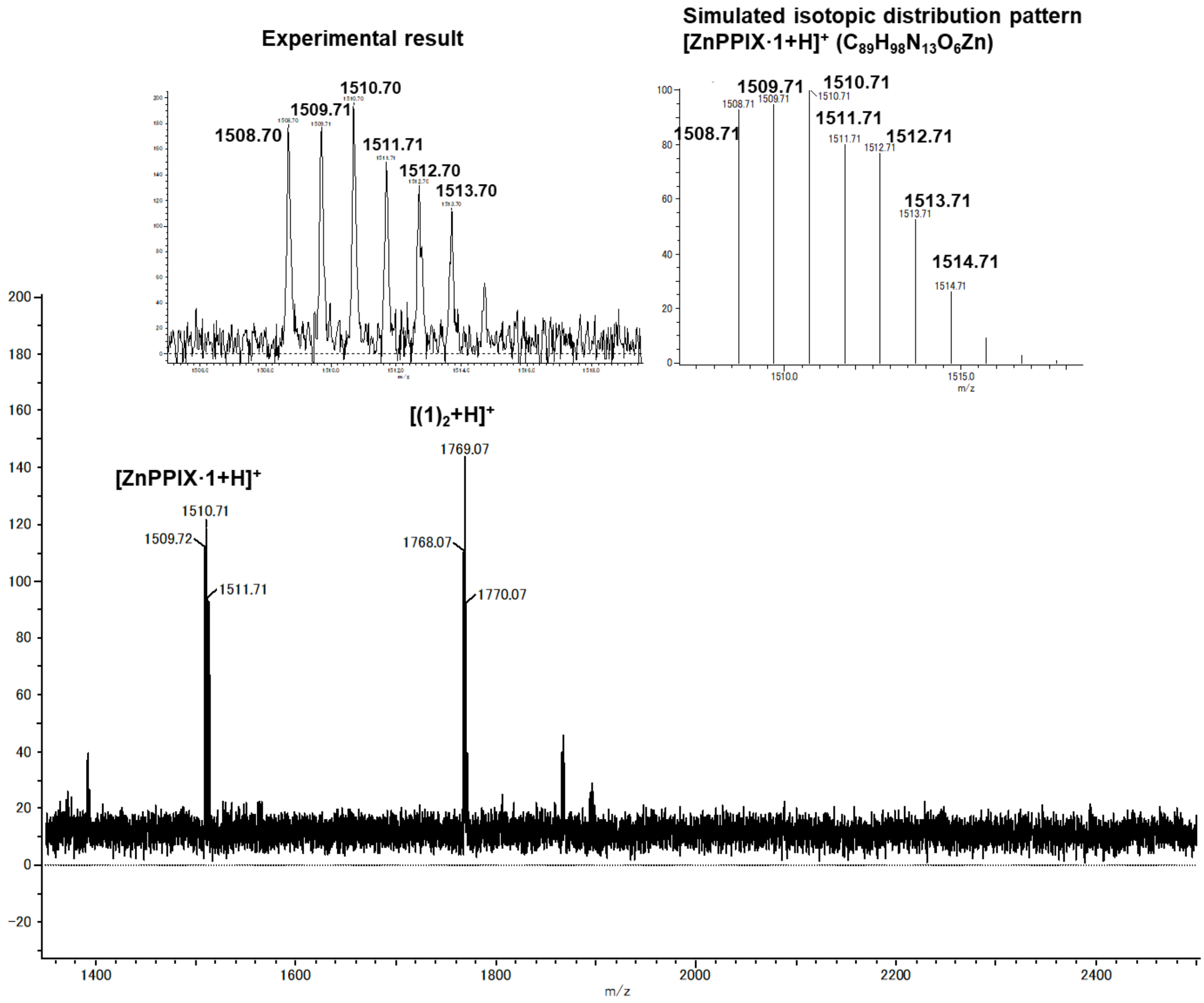

Figure S26. ESI mass spectra of ZnPPIX·**1** complex. [**1**] = 50 μM and [ZnPPIX] = 50 μM in DMSO/MeOH/H_2O = 0.1:2:1 (v/v/v)

(a)

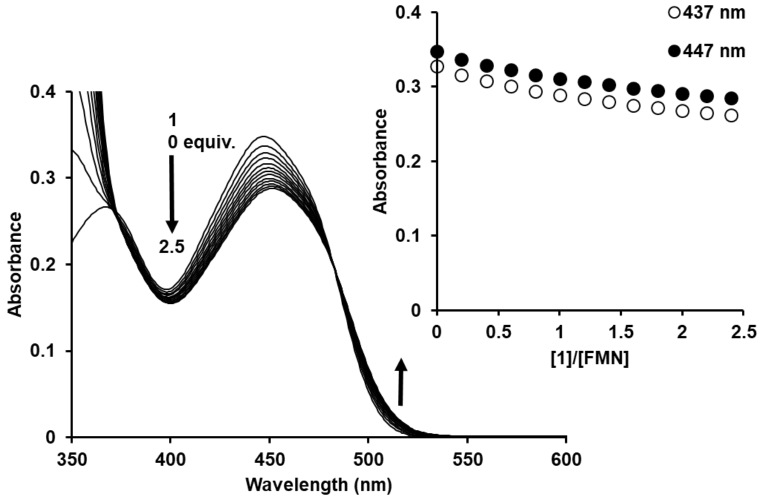

(b)

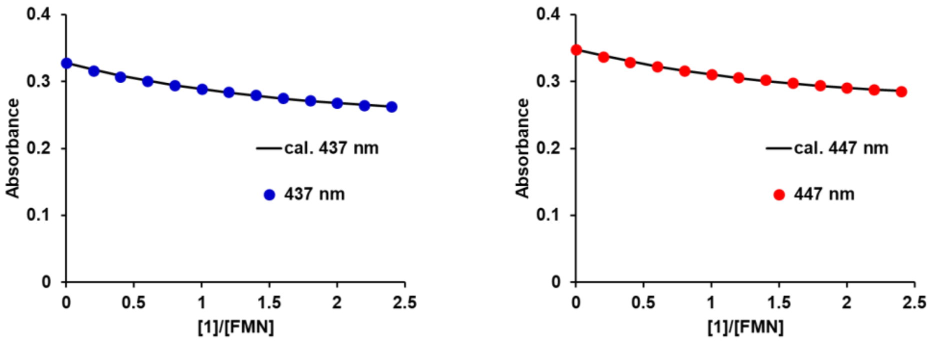

(c)

Exp.	K_{11}/M^{-1} [a]	URL
Entry 1	3.5 × 10^{4} (Error: ±2.4%)	http://app.supramolecular.org/bindfit/view/19a47fbd-3f6d-4dbb-91a6-3cb2ef83f535
Entry 2	3.7 × 10^{4} (Error: ±1.7%)	http://app.supramolecular.org/bindfit/view/162e8fbf-6746-4c87-9868-342b2f168c5a
Entry 3	3.9 × 10^{4} (Error: ±2.2%)	http://app.supramolecular.org/bindfit/view/a0f3bd09-6fea-49b5-a6bf-6a125acab807
Mean	3.7 × 10^{4}	
Standard deviation	0.21 × 10^{4}	

[a] Calculated by 1:1 fitting model using the changes in absorbance at 437 nm and 447 nm

Figure S27. (a) UV-Vis titration of FMN (30 μM) with **1** in DMSO/33 mM HEPES buffer (pH 7.4) = 2:3 (v/v) at 25 °C. Inset: Changes in absorbance of FMN upon the addition of **1**. (b) Typical data of titration curves fitted to a 1:1 binding model (Nelder-Mead) using Bindfit (entry 1). (b) Summary

(a) (b)

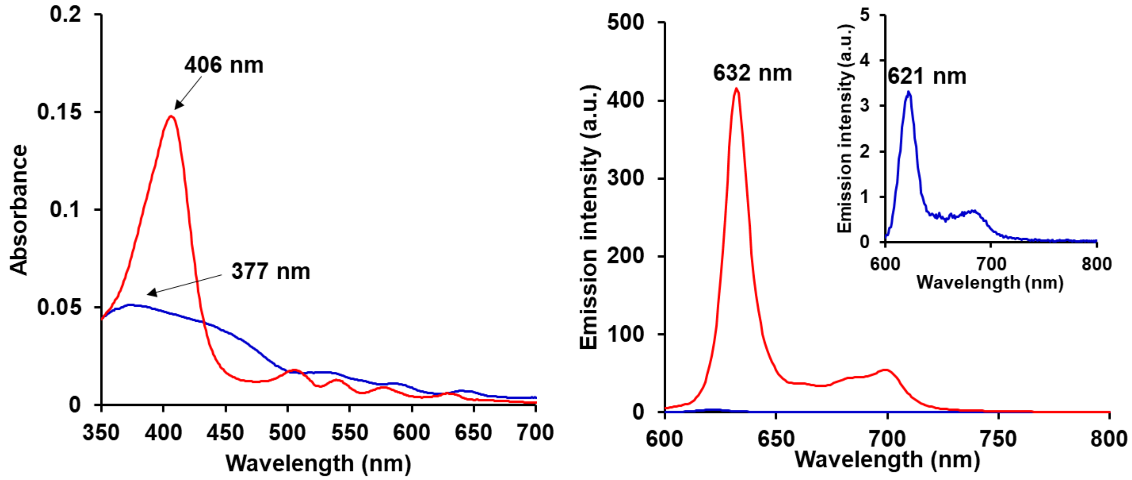

Figure S28. (a) UV-Vis spectra and (b) fluorescence emission spectra (excitation at 545 nm) of PPIX (1.0 μM) (blue line) and PPIX (1.0 μM) containing 1% Triton X-100 (red line) in DMSO/DMEM = 1:99 (v/v) at 25 °C. Inset: Expanded fluorescence emission spectrum of PPIX (1.0 μM) (blue line) in DMSO/DMEM = 1:99 (v/v).

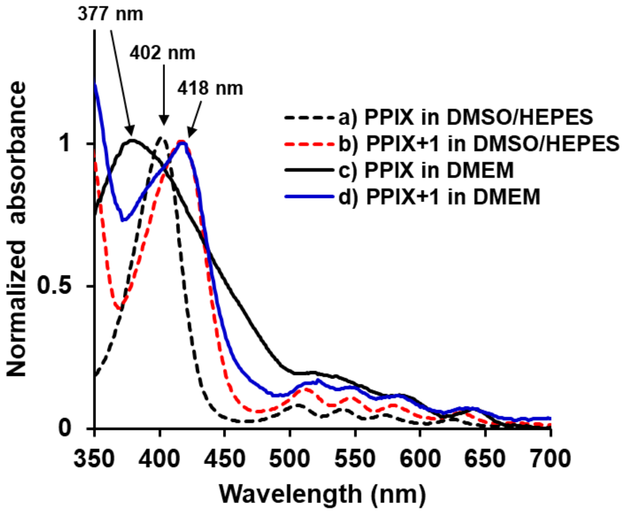

Figure S29. Normalized UV-Vis spectra of (a) PPIX (2.4 μM) alone and (b) PPIX (2.4 μM) + **1** (4.8 μM) in DMSO/33 mM HEPES buffer (pH 7.4) = 2:3 (v/v). Normalized UV-Vis spectra of (c) PPIX (1.0 μM) alone and (d) PPIX (1.0 μM) + **1** (2.0 μM) in DMSO/DMEM = 1:99 (v/v). Temp. 25 °C.

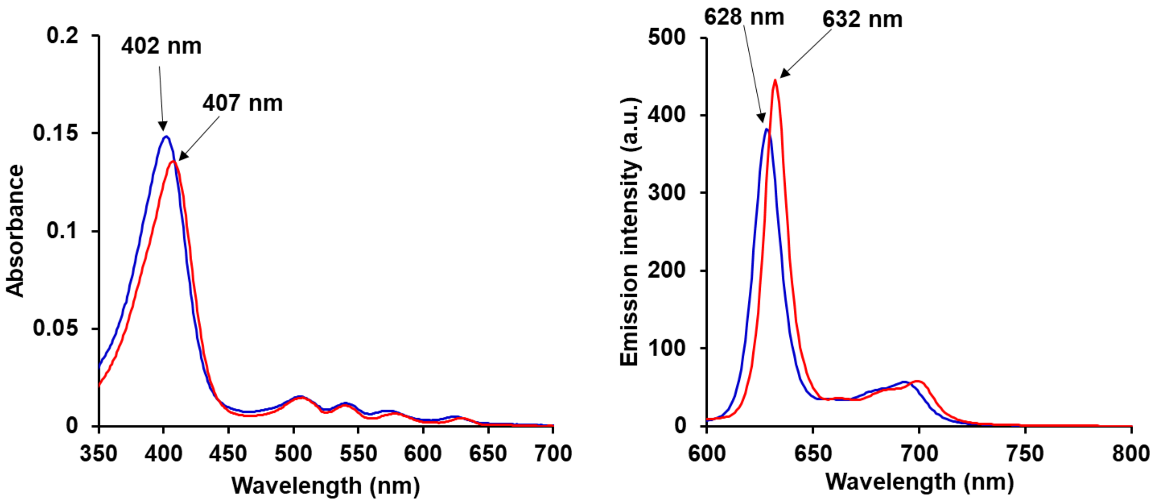

Figure S30. (a) UV-Vis spectra and (b) fluorescence emission spectra (excitation at 545 nm) of PPIX (1.0 μM) (blue line) and PPIX (1.0 μM) containing 1% Triton X-100 (red line) in DMSO/33 mM HEPES buffer pH 7.4 = 2:3 (v/v) at 25 ºC. The emission intensity of PPIX (λ_{em} = 632 nm) in the presence of 1% Triton X-100 is ca. 1.1-fold higher than that of PPIX alone (λ_{em} = 628 nm).

(a)

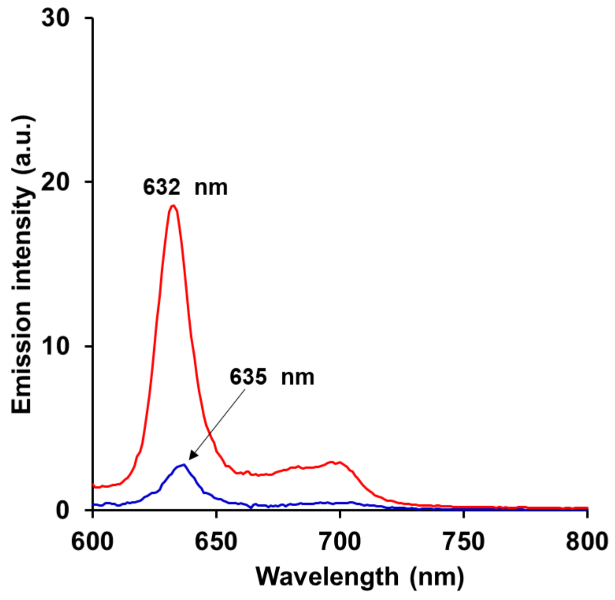

(b)

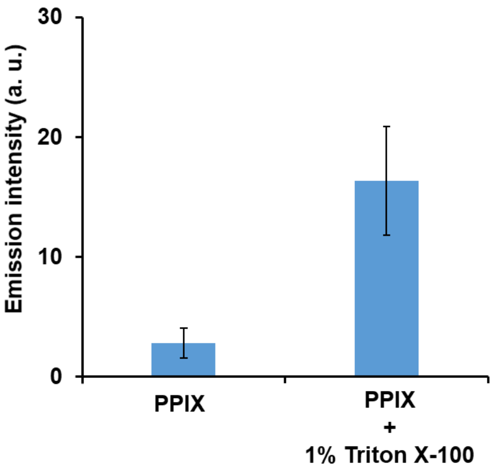

(c)

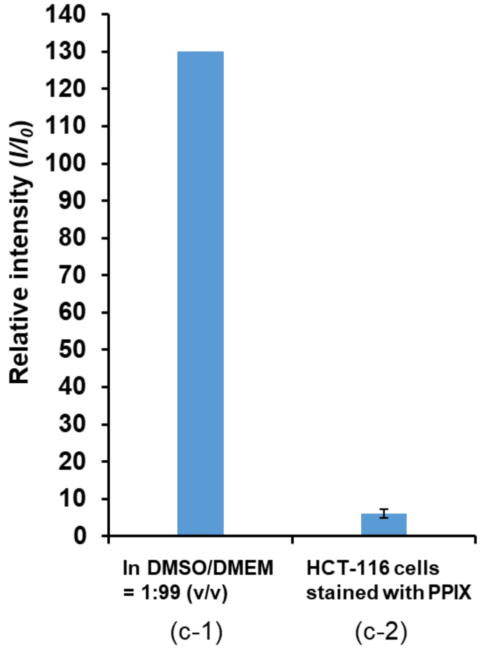

Figure S31. (a) Fluorescence emission spectra of HCT-116 cells stained with PPIX (1.0 μM) in the absence (blue line) and presence (red line) of 1% Triton X-100 in DMEM at 25 ºC (excitation at 545 nm). (b) Emission intensity of HCT-116 cells stained with PPIX (1.0 μM) in the absence (λ_{em} = 635 nm) and presence (λ_{em} = 632 nm) of 1% Triton X-100 in DMEM. (c-1) Relative emission intensity of PPIX in DMSO/DMEM = 1:99 (v/v) in the absence and presence of 1% Triton X-100 (Fig. S28b). I_0: emission intensity of PPIX at 621 nm, I: emission intensity of PPIX at 632 nm after treatment with 1% Triton X-100. (c-2) Relative emission intensity of HCT-116 cells stained with PPIX in the absence and presence of 1% Triton X-100. I_0: emission intensity of HCT-116 cells stained with PPIX at 635 nm, I: emission intensity of HCT-116 cells stained with PPIX at 632 nm after treatment with 1% Triton X-100. (b) and (c-2) The data for the emission intensity from HCT-116 cells stained with PPIX represent the mean±standard deviation of triplicate experiments.

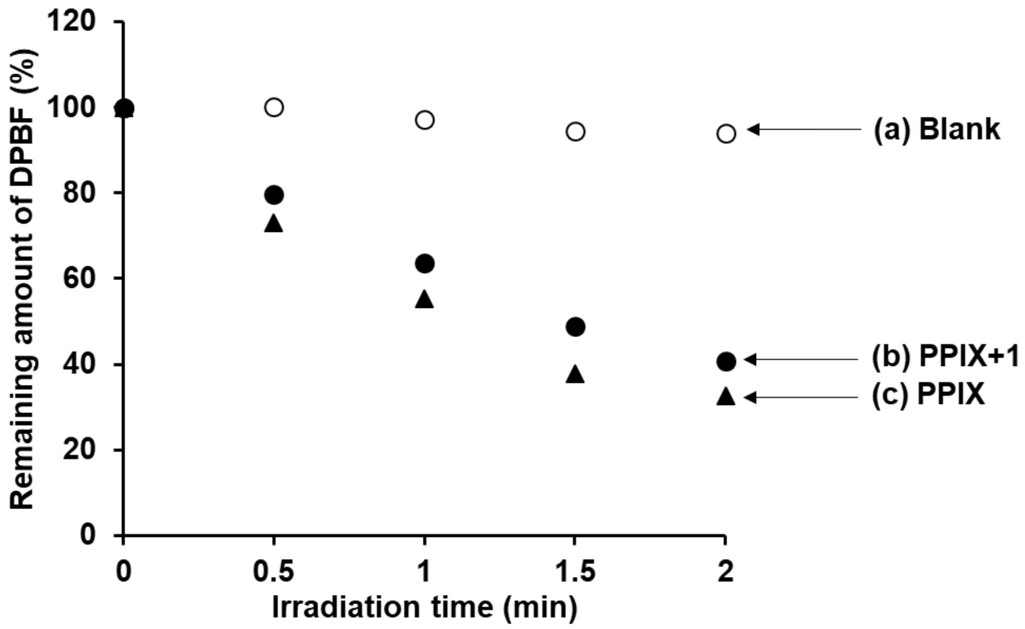

Figure S32. Degradation of DPBF (10 μM) in O_2 saturated DMSO/33 mM HEPES buffer pH 7.4 = 2:3 (v/v) at 25 ºC upon photoirradiation at 530-590 nm (20 mW/cm^2 at 550 nm) using xenon light source (Max302, Asahi Spectra). (a) Blank (open circles), (b) PPIX (0.60 μM) + **1** (1.2 μM) (closed circles), (c) PPIX (0.60 μM) alone (closed triangles). Remaining amount of DPBF (%) was evaluated from the decrease in absorbance of DPBF at 415 nm.

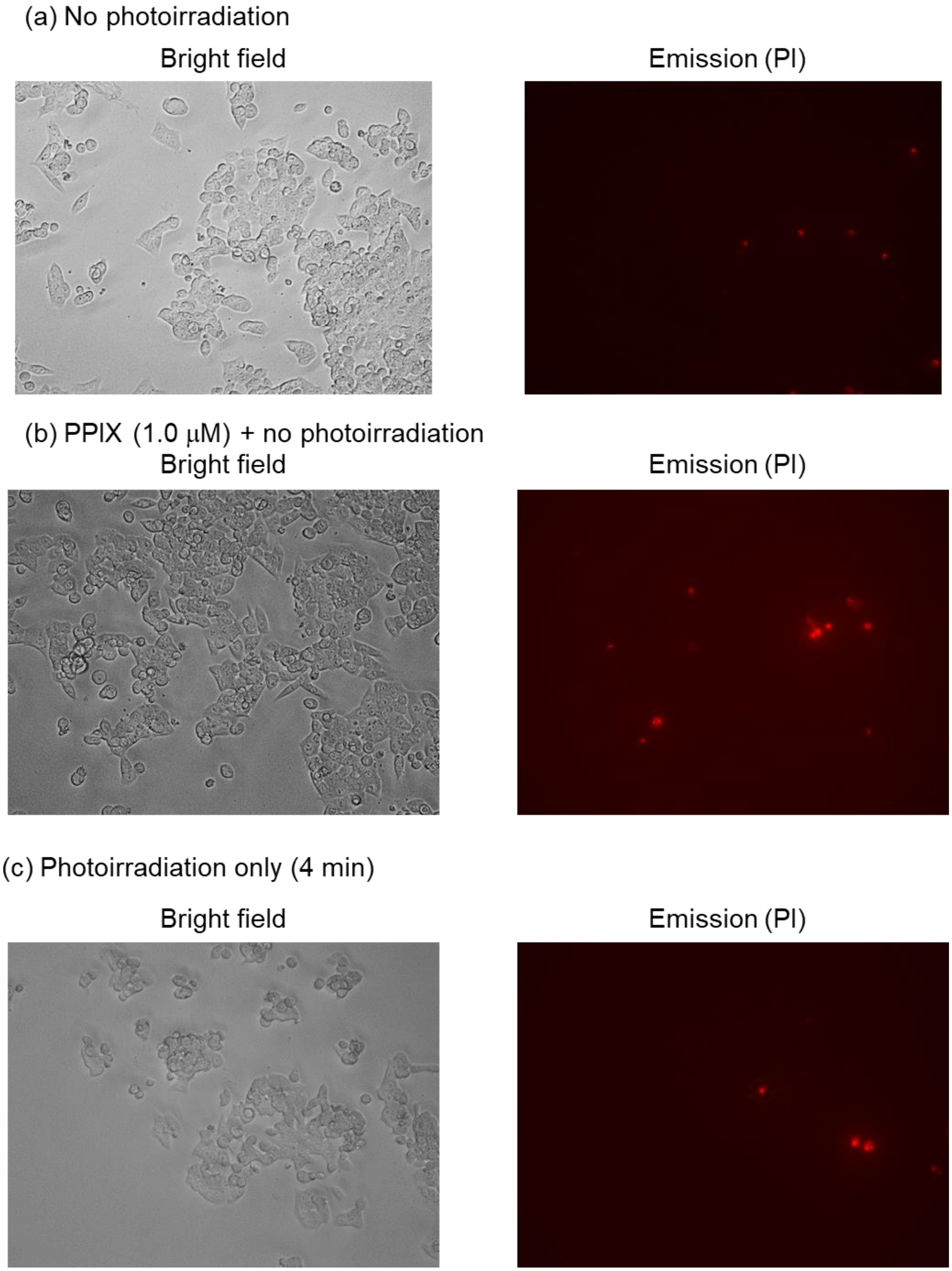
(a) No photoirradiation
Bright field
Emission (PI)
(b) PPIX (1.0 μM) + no photoirradiation
Bright field
Emission (PI)
(c) Photoirradiation only (4 min)
Bright field
Emission (PI)

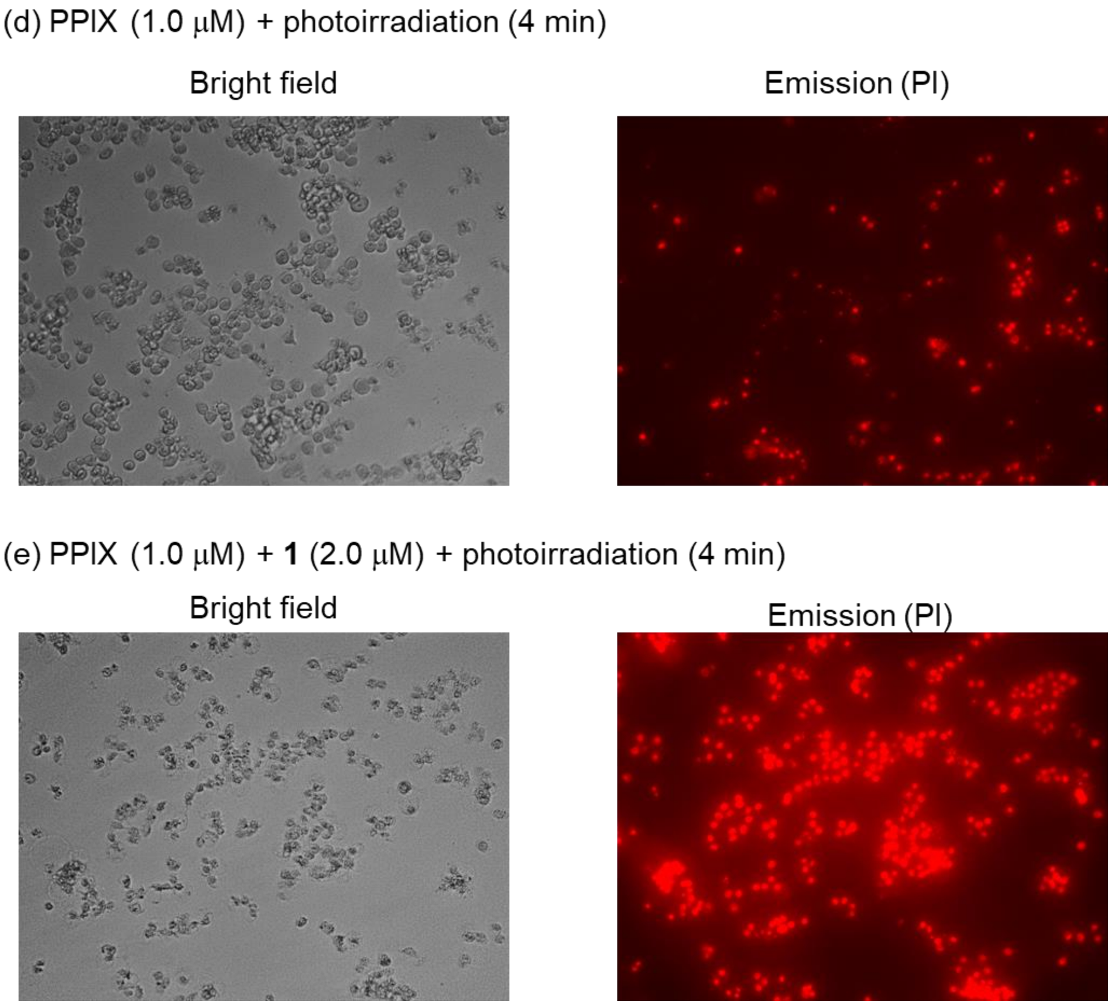

Figure S33. Typical fluorescence microscopy images (Biorevo BZ-9000 with TRITC filter (Ex.540±13 nm, Em.605±28 nm)) of HCT-116 cells (magnification: ×20) after irradiation at 530-590 nm. The dead cells were detected by emission of PI. (a) No photoirradiation, (b) PPIX (1.0 μM) + no photoirradiation, (c) photoirradiation only (4min), (d) PPIX (1.0 μM) + photoirradiation for 4 min, (e) PPIX (1.0 μM) + **1** (2.0 μM) + photoirradiation for 4 min.

References:

[S1] M. V. N. de Souza, K. C. Pais, C. R. Kaiser, M. A. Peralta, M. de L. Ferreira and M. C. S. Lourenço *Bioorg. Med. Chem.* 2009, **17**, 1474.

[S2] (a) Bindfit (http://supramolecular.org) (b) P. Thordarson, *Chem. Soc. Rev.* 2011, **40**, 1305.

[S3] K. Hirose, *J. Inclusion Phenom. Macrocyclic Chem*. 2001, **39**, 193.

[S4] A. Leed, K. DuBay, L. M. B. Ursos, D. Sears, A. C. de Dios and P. D. Roepe, *Biochemistry* 2002, **41**, 10245.

[S5] S. Carvalho, R. Delgado, N. Fonsecac and V. Félix, *New J. Chem*. 2006, **30**, 247.

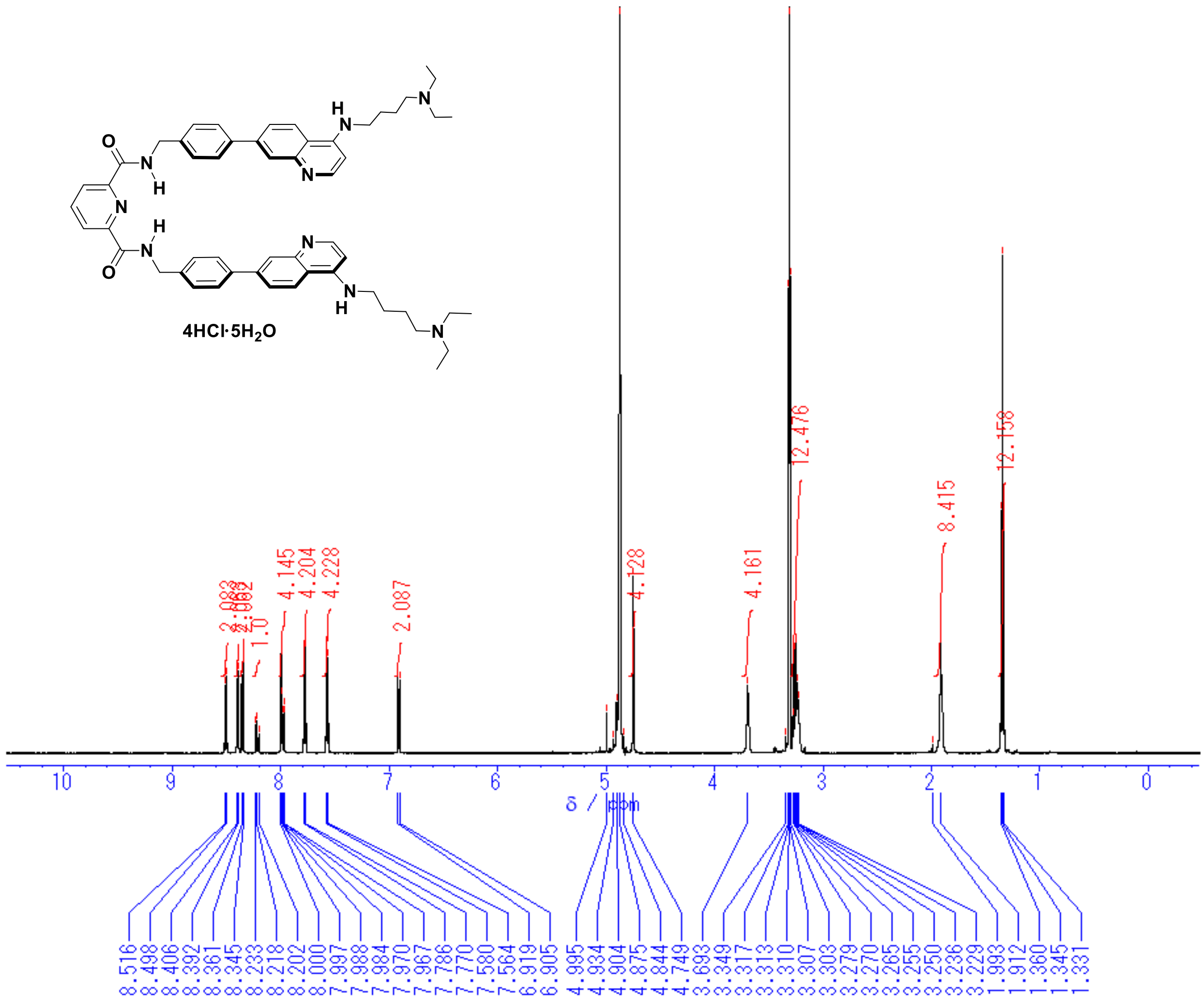

Figure S34. ^{1}H NMR spectrum (CD_3OD) of **1**·4HCl·5H_2O.

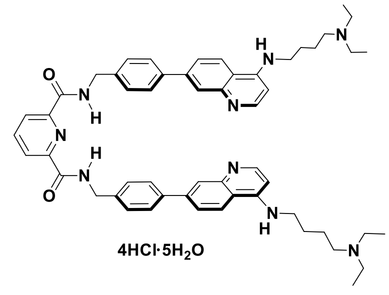

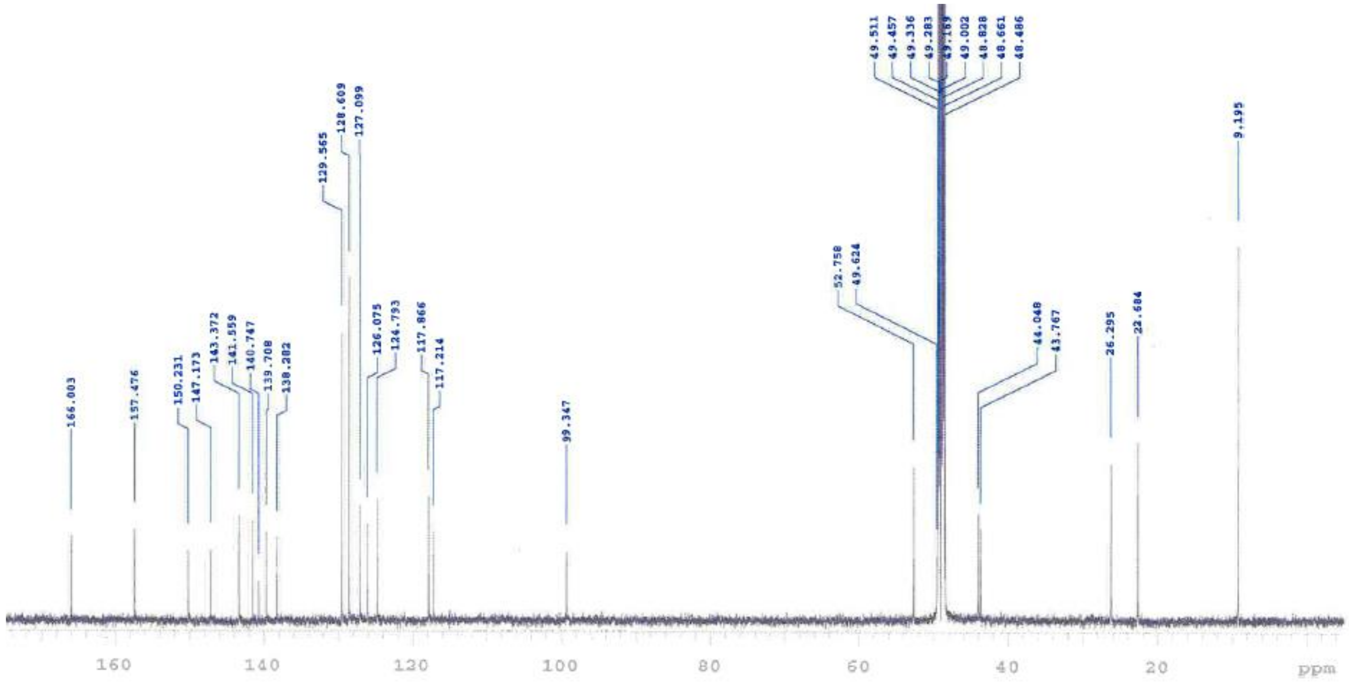

Figure S35. ^{13}C NMR spectrum (CD_3OD) of **1**·4HCl·$5H_2O$.

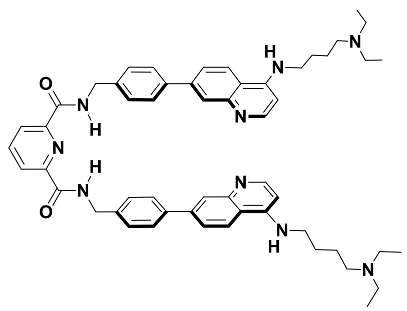

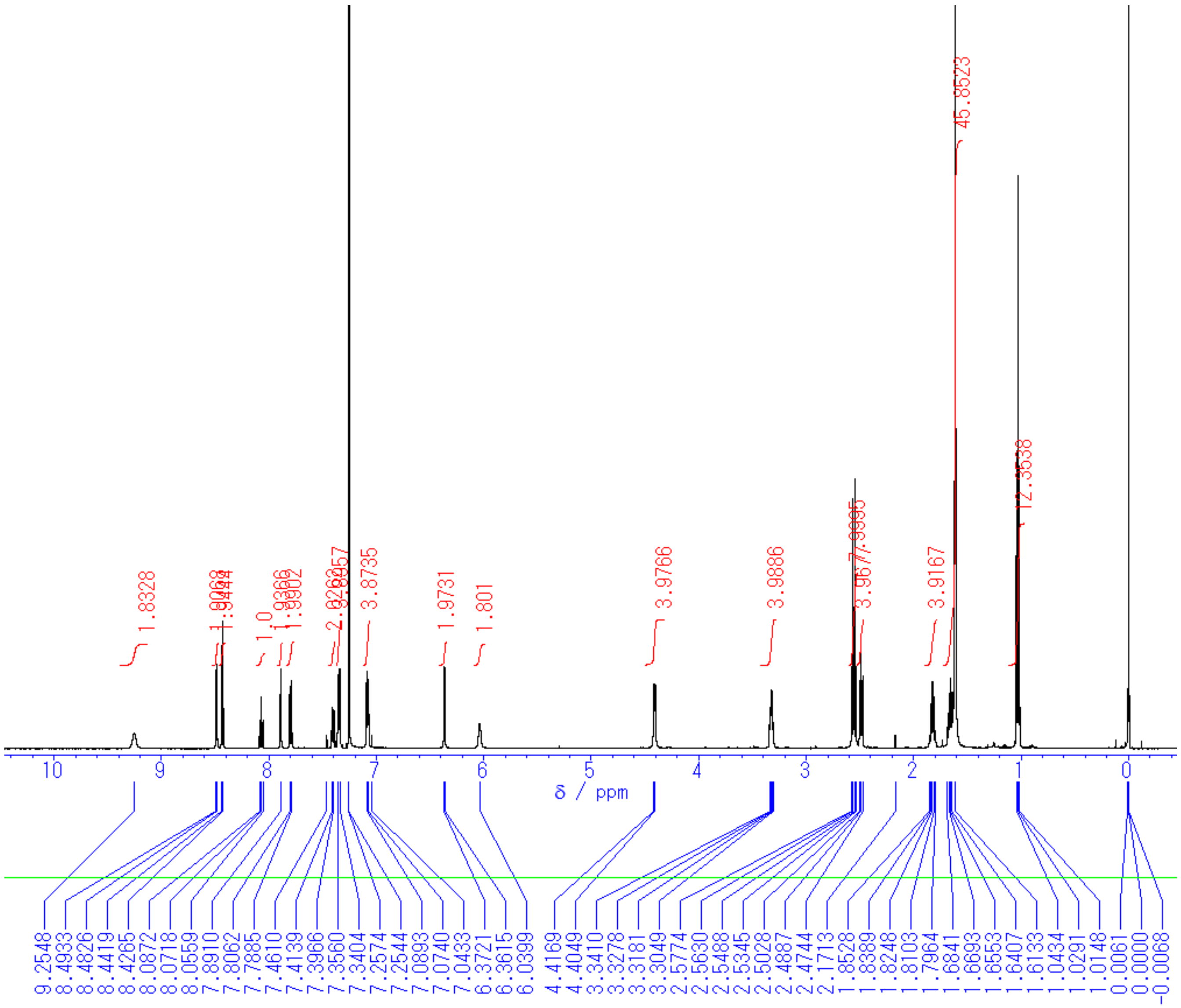

Figure S36. ¹H NMR spectrum ($CDCl_3$) of **1** (free base).

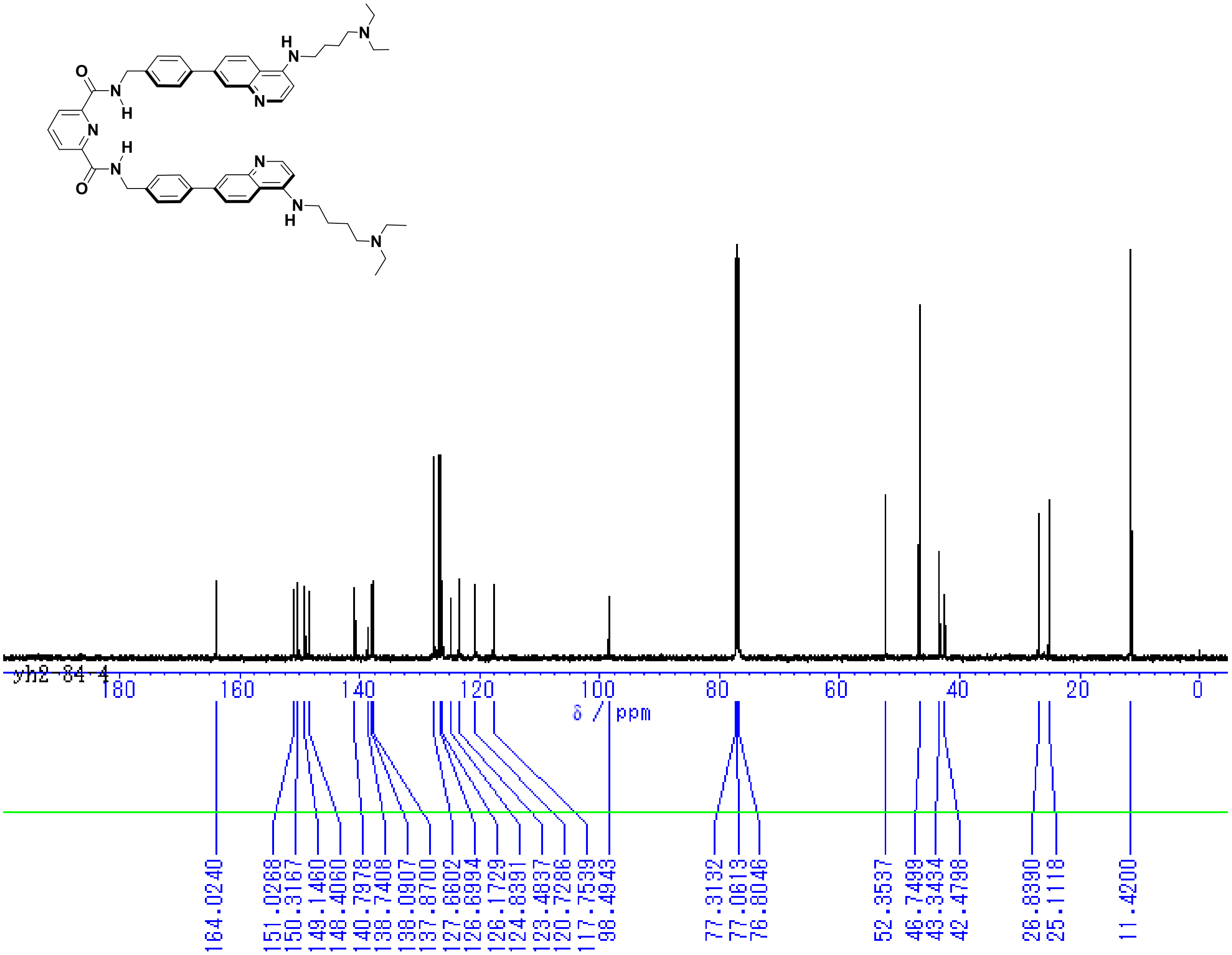

Figure S37. ^{13}C NMR spectrum ($CDCl_3$) of **1** (free base).

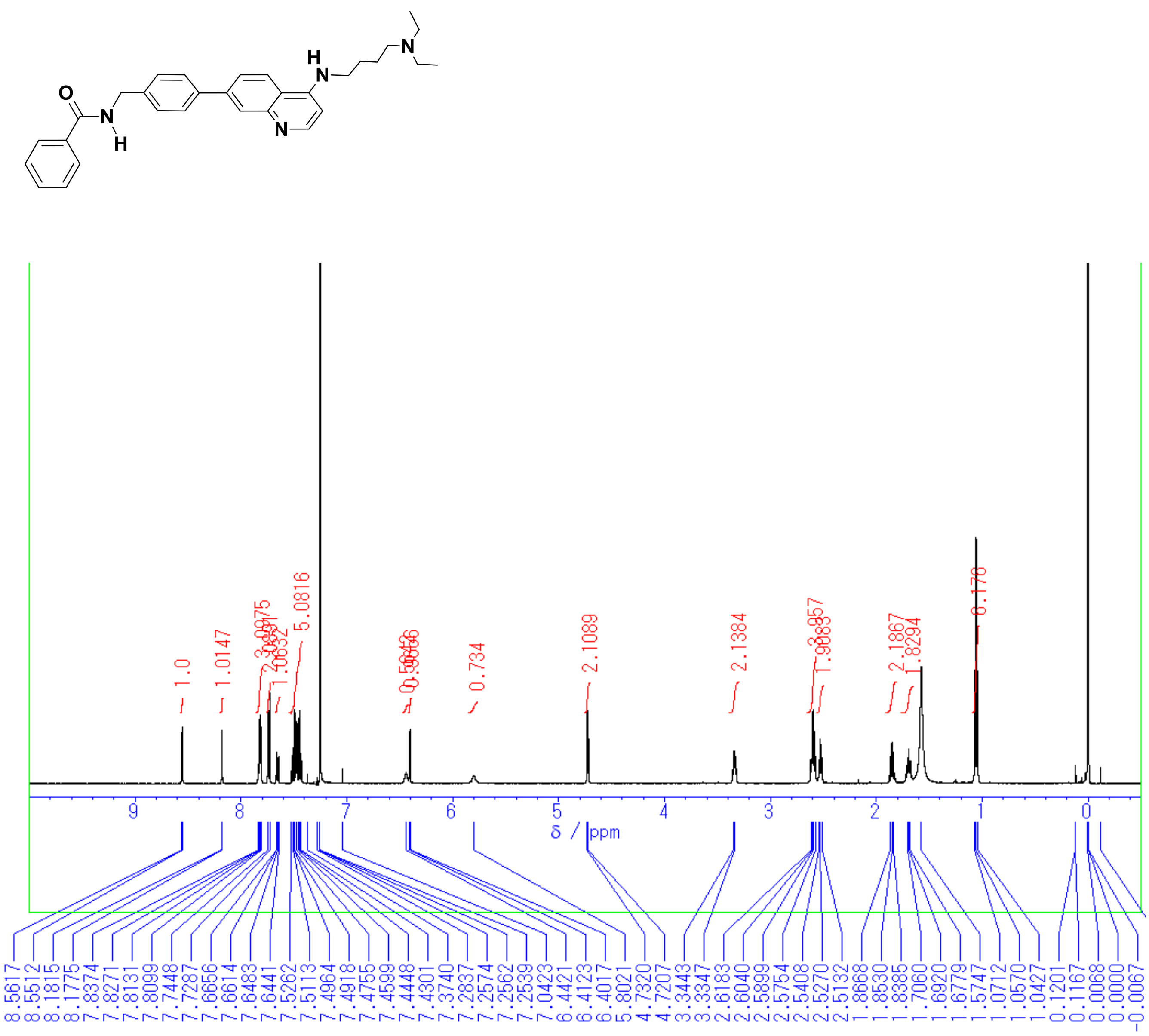

Figure S38. ^{1}H NMR spectrum ($CDCl_3$) of **2.**

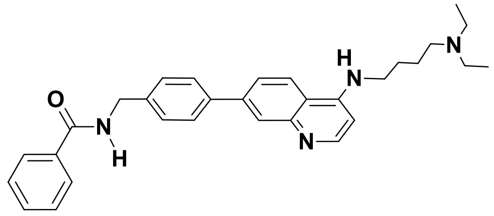

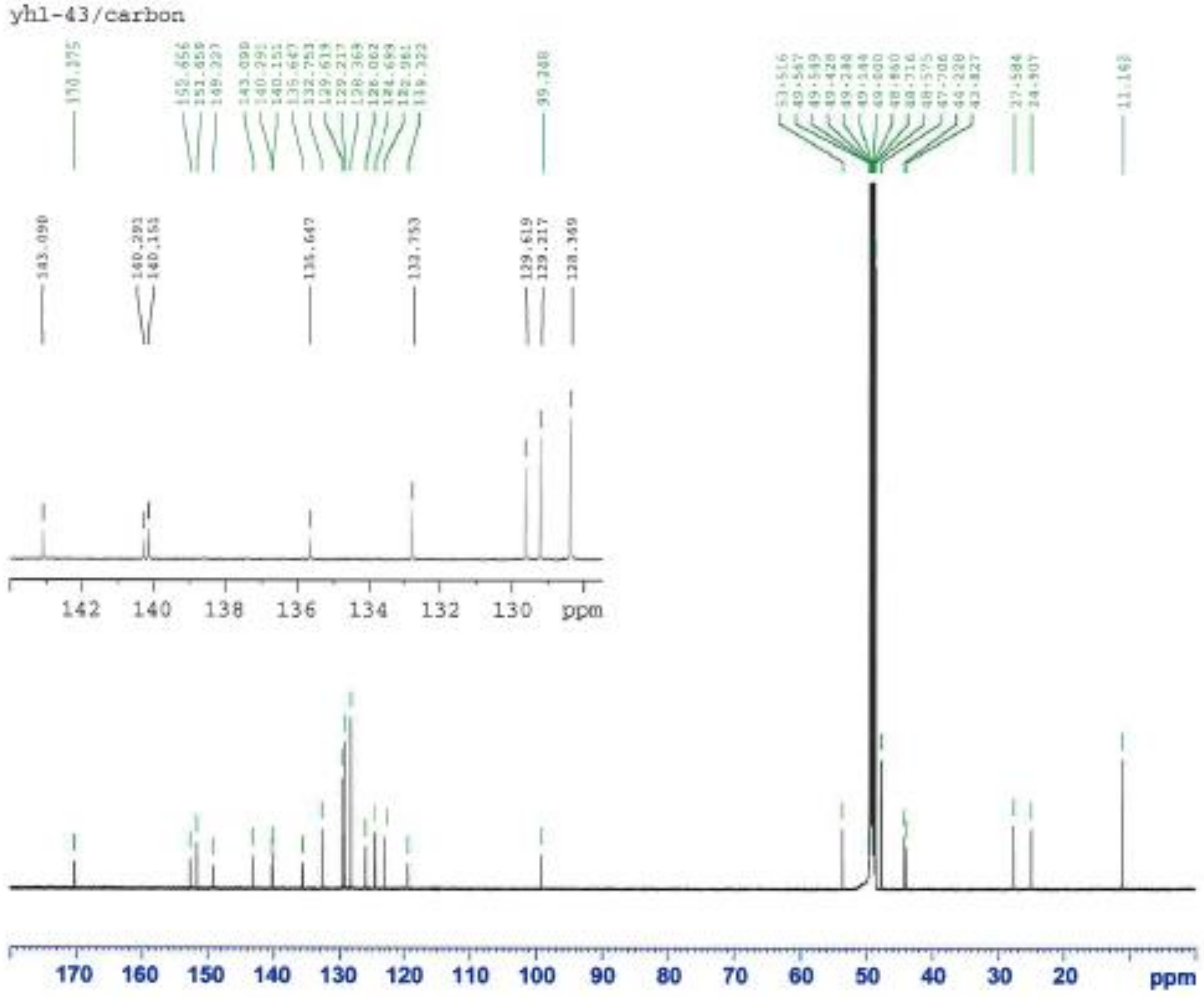

Figure S39. ^{13}C NMR spectrum ($CDCl_3$) of **2.**

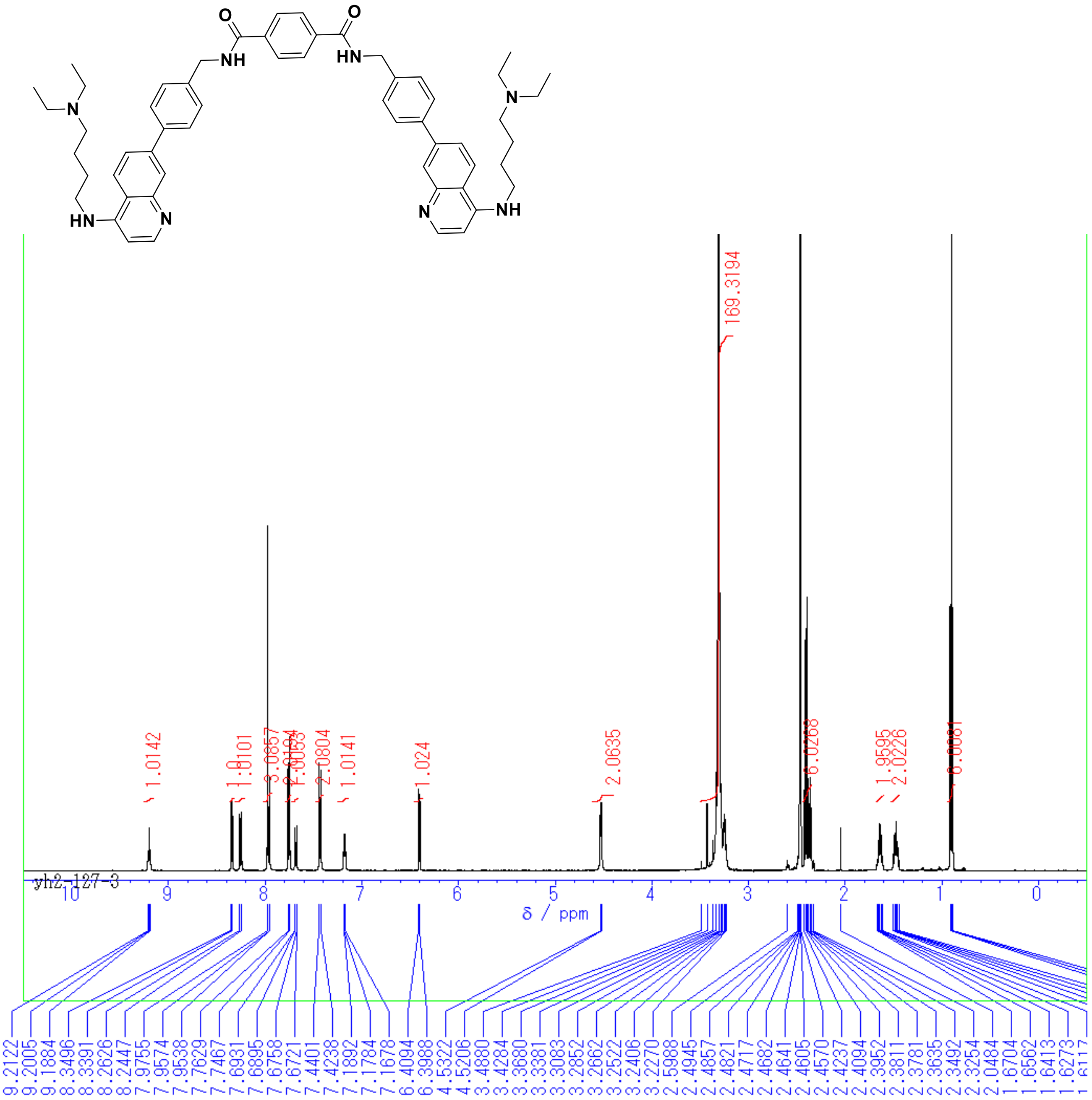

Figure S40. ^{1}H NMR spectrum (DMSO-d_6) of **3.**

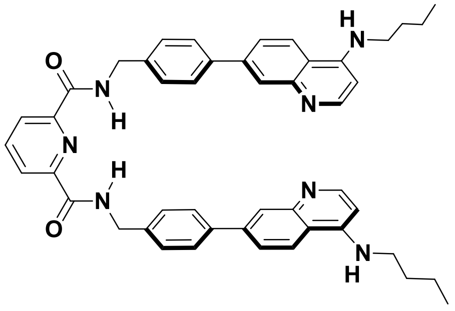

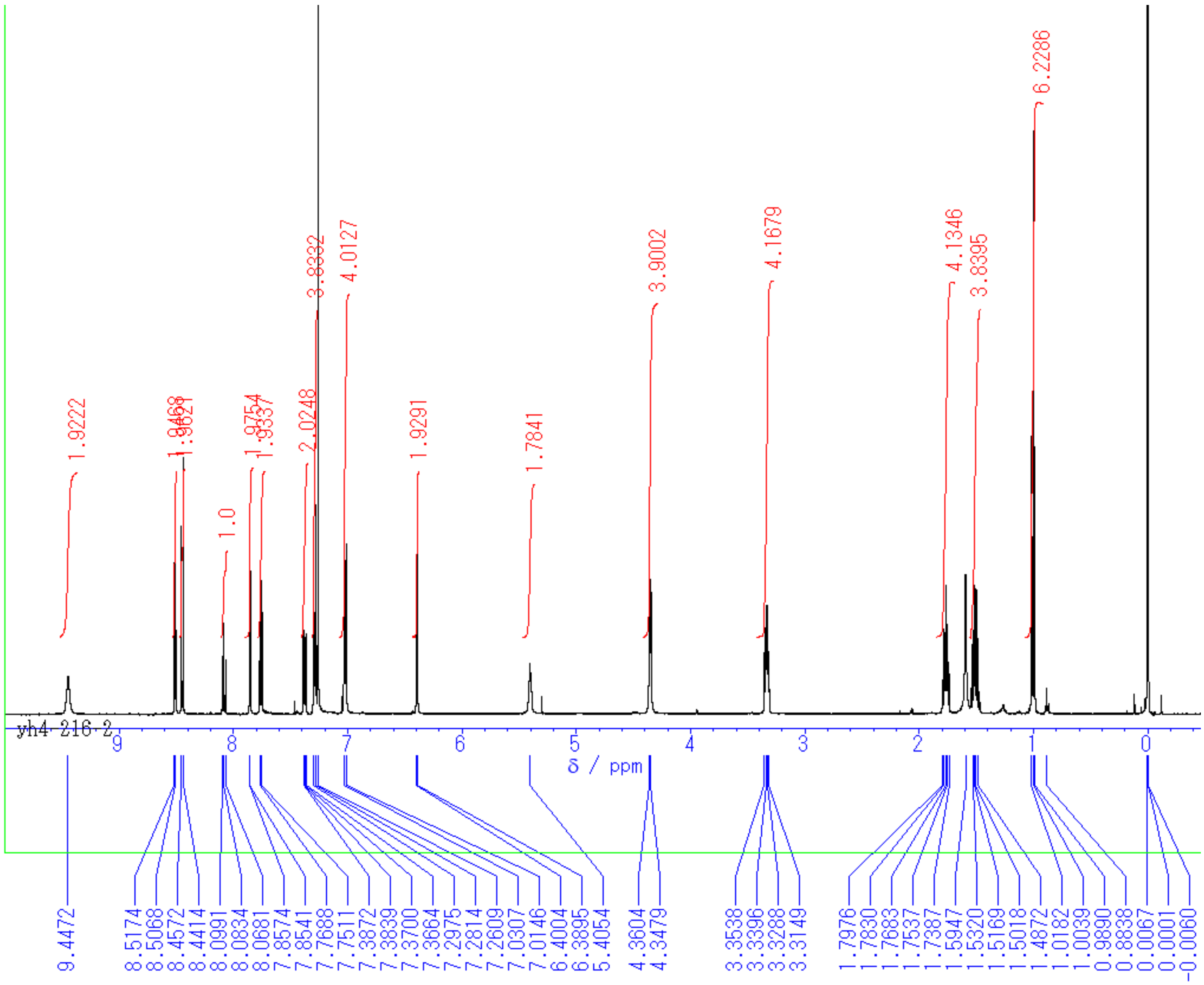

Figure S41. ^{1}H NMR spectrum ($CDCl_3$) of **4.**

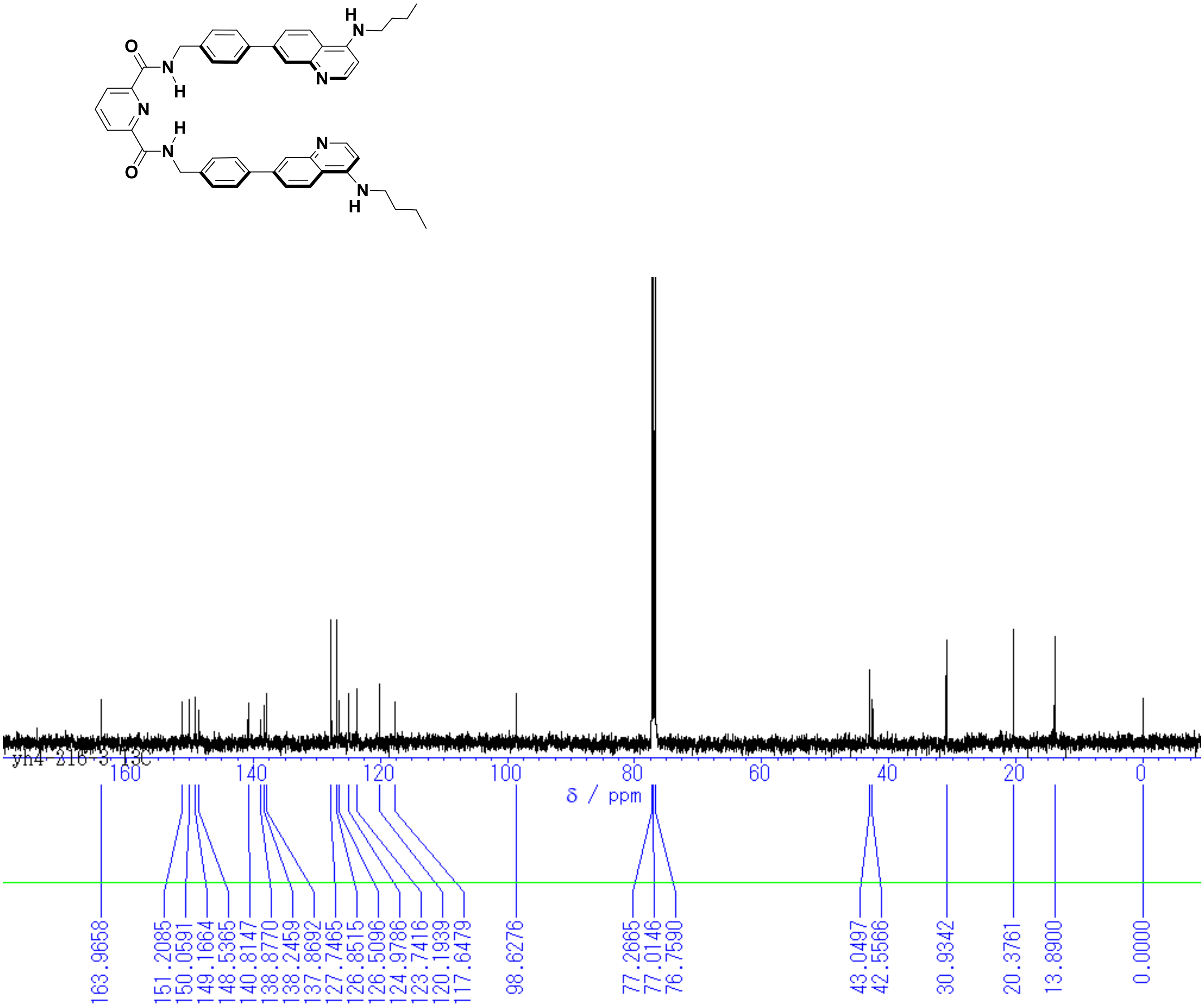

Figure S42. ^{13}C NMR spectrum ($CDCl_3$) of **4.**

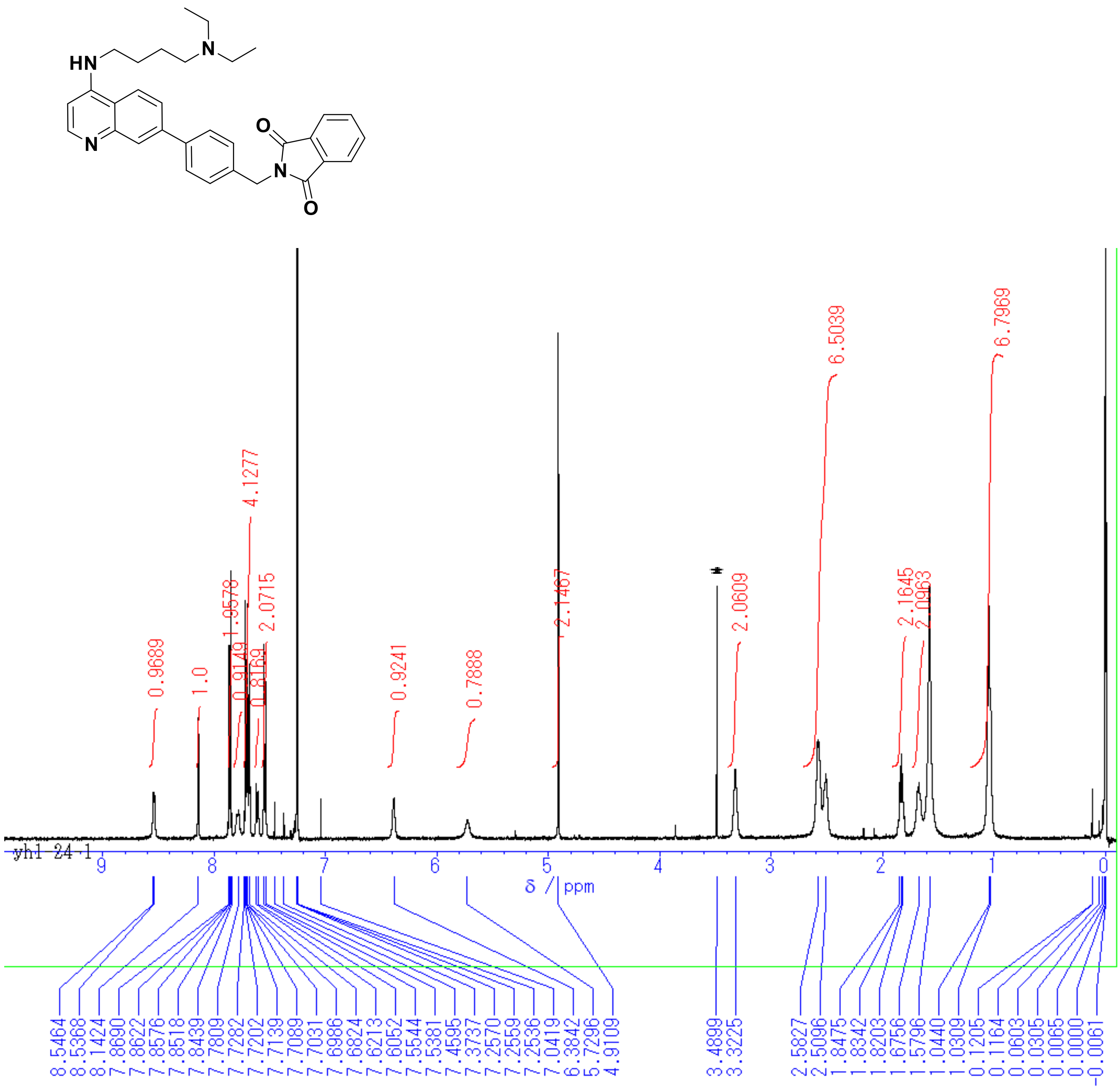

Figure S43. ^{1}H NMR spectrum ($CDCl_3$) of **7**. An asterisk indicates proton signal of MeOH.

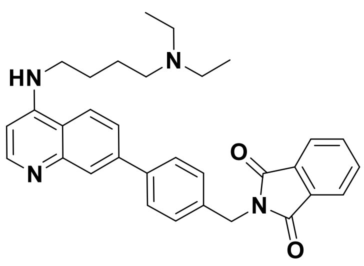

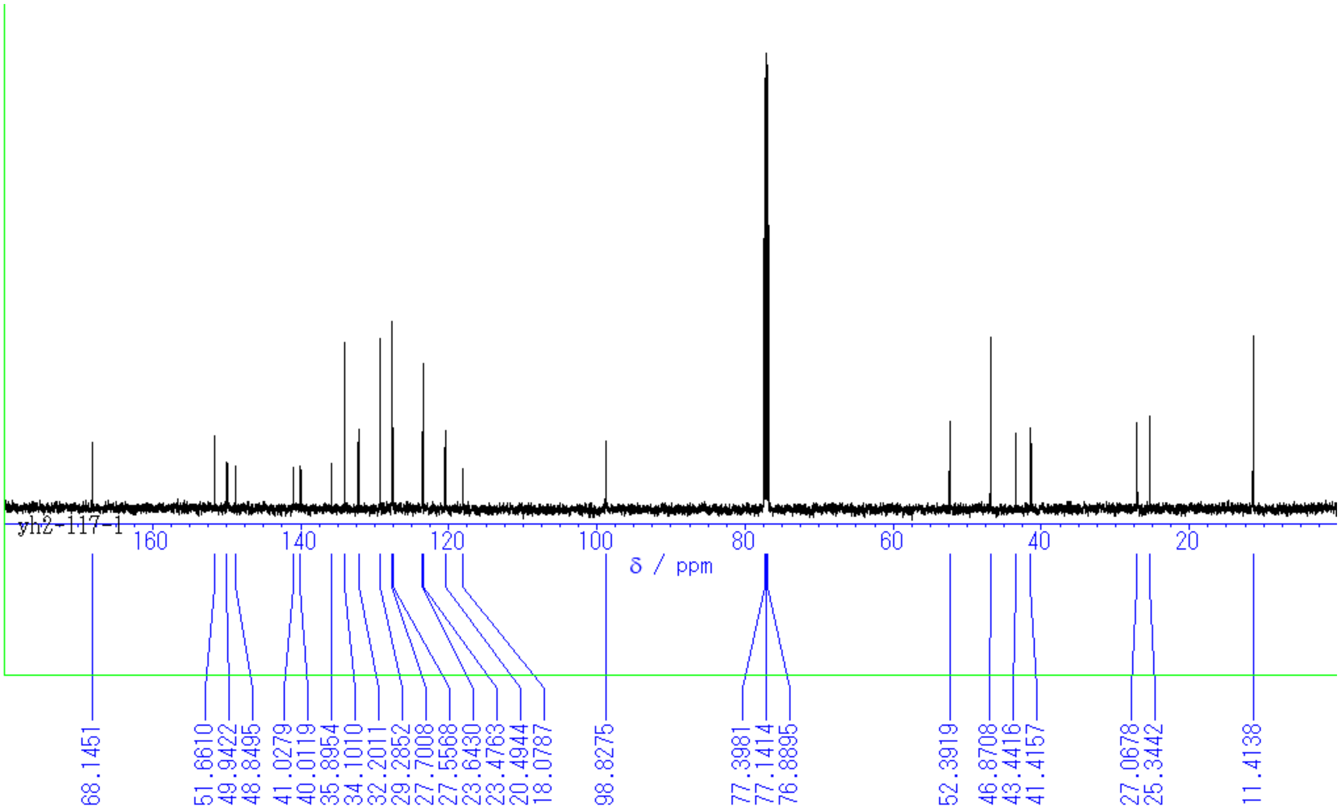

Figure S44. ^{13}C NMR spectrum ($CDCl_3$) of **7**.

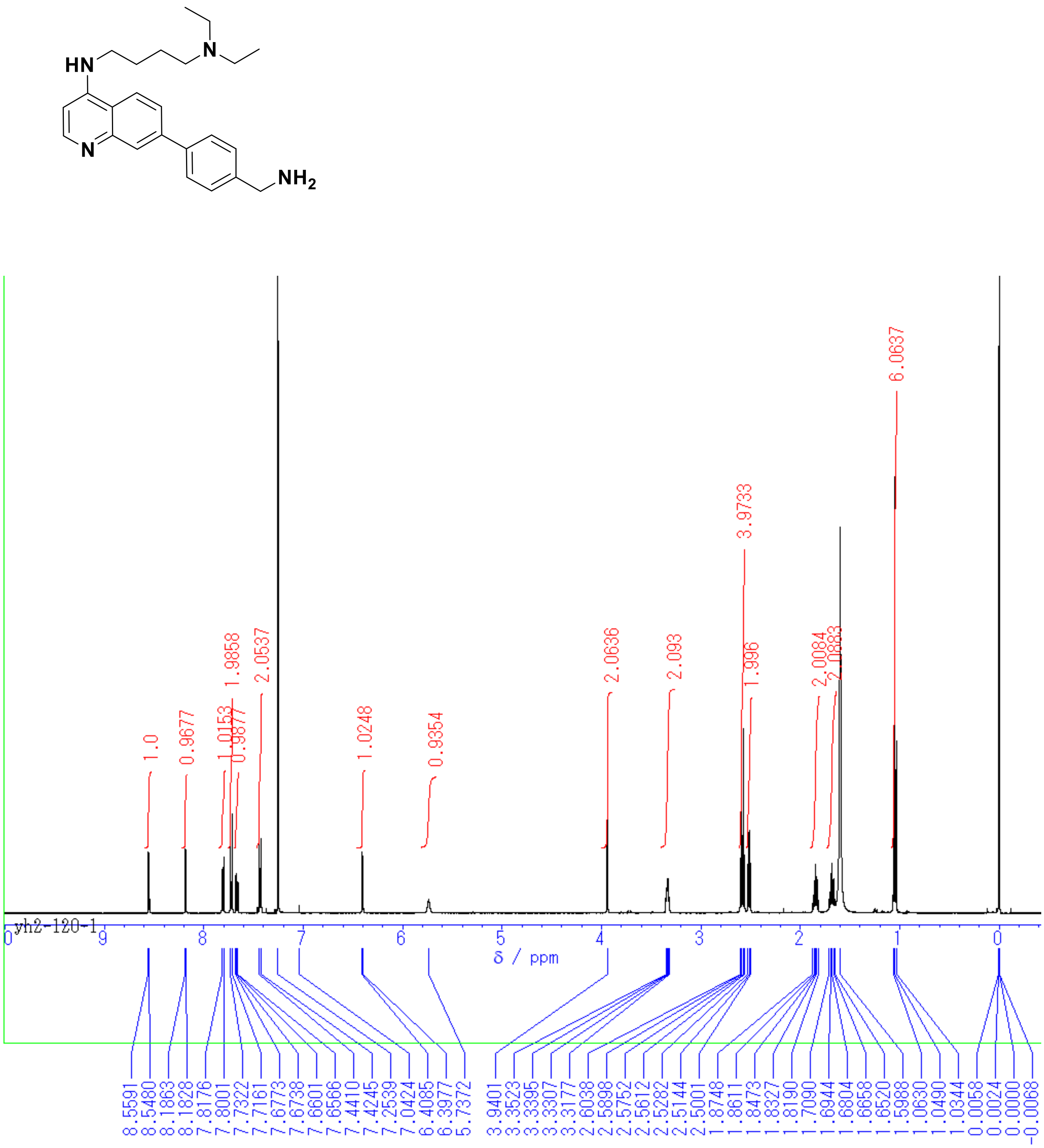

Figure S45. ^{1}H NMR spectrum ($CDCl_3$) of **8**.

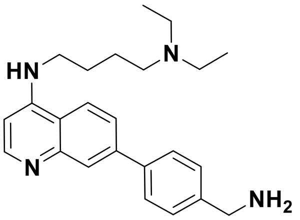

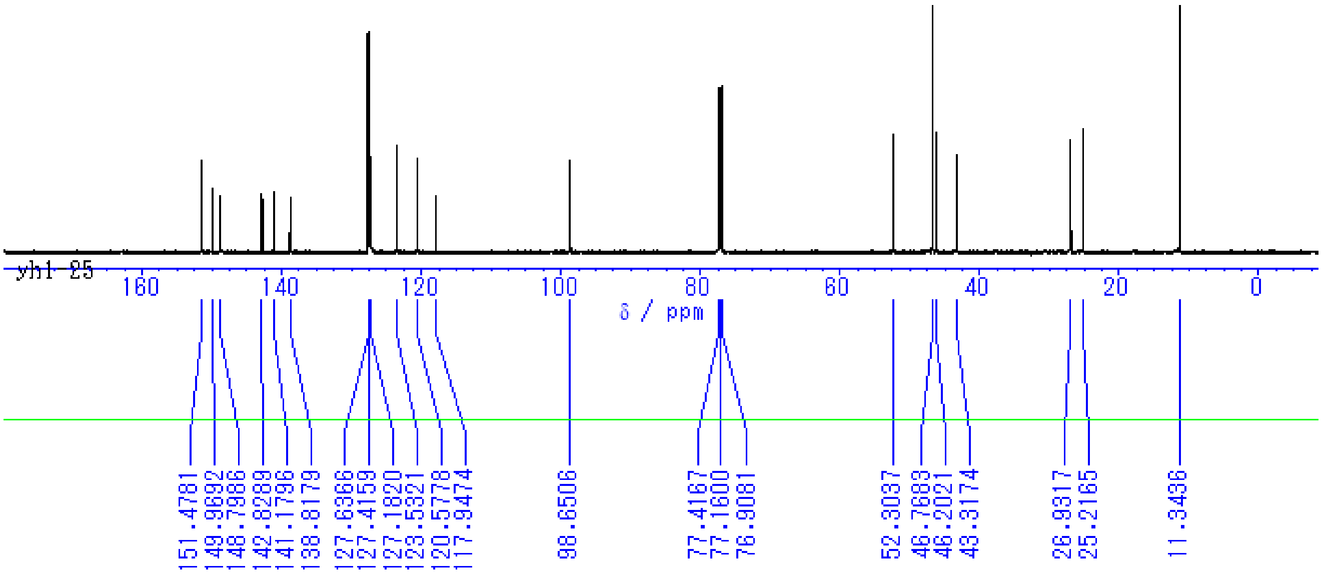

Figure S46. ^{13}C NMR spectrum ($CDCl_3$) of **8.**

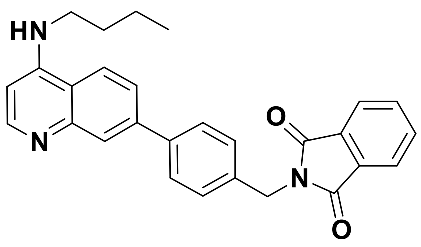

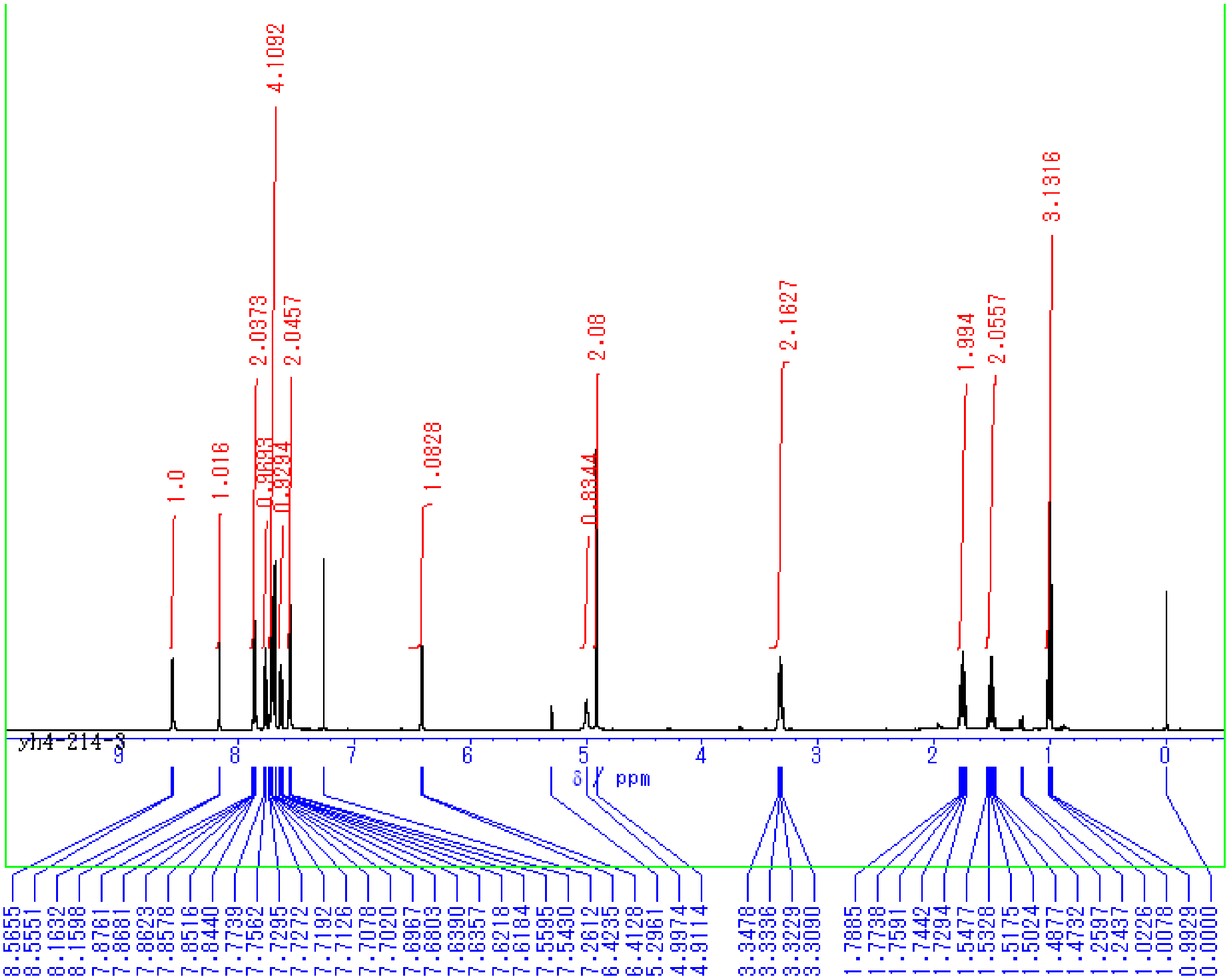

Figure S47. ^{1}H NMR spectrum ($CDCl_3$) of **10.**

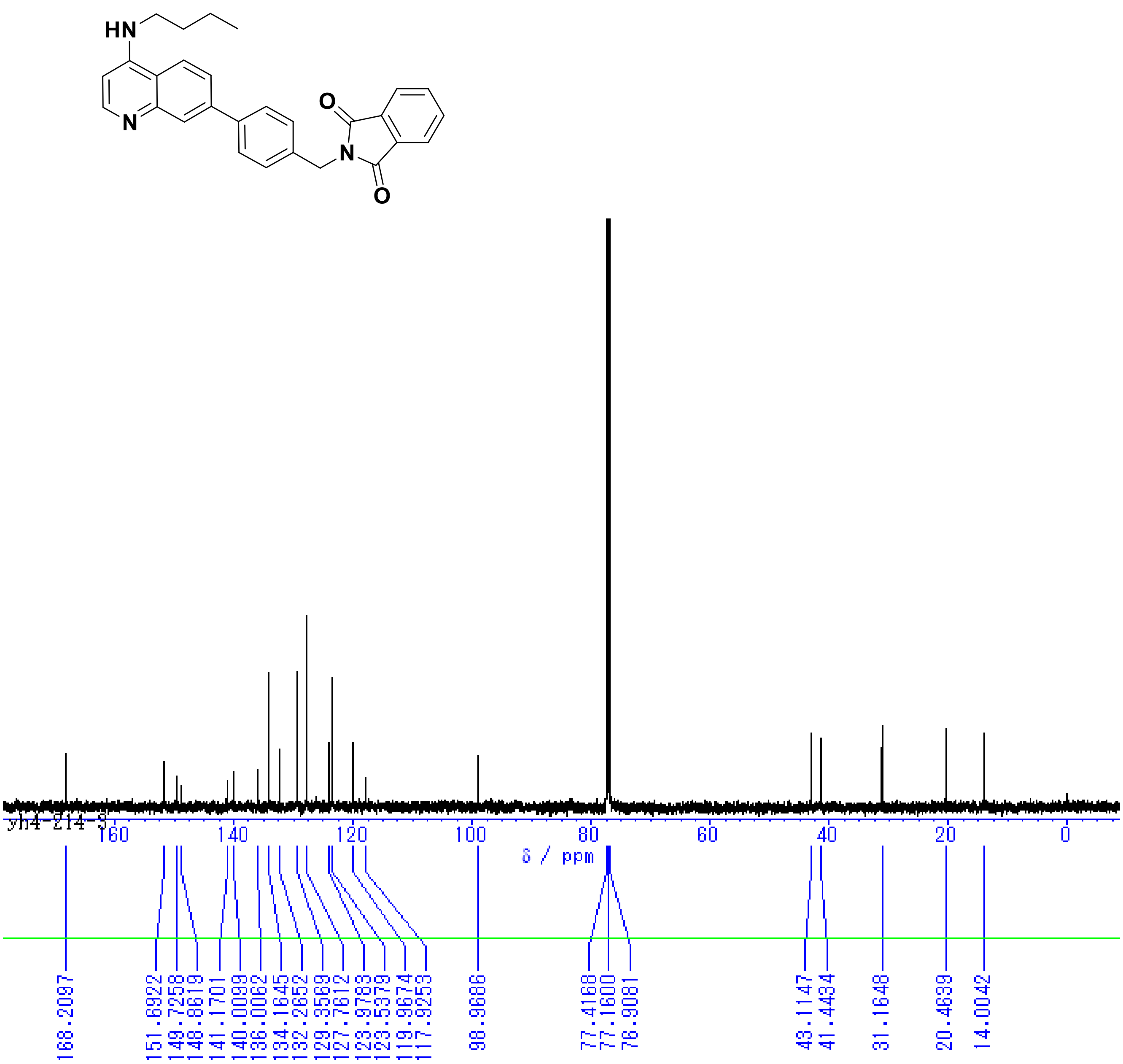

Figure S48. ^{13}C NMR spectrum ($CDCl_3$) of **10.**

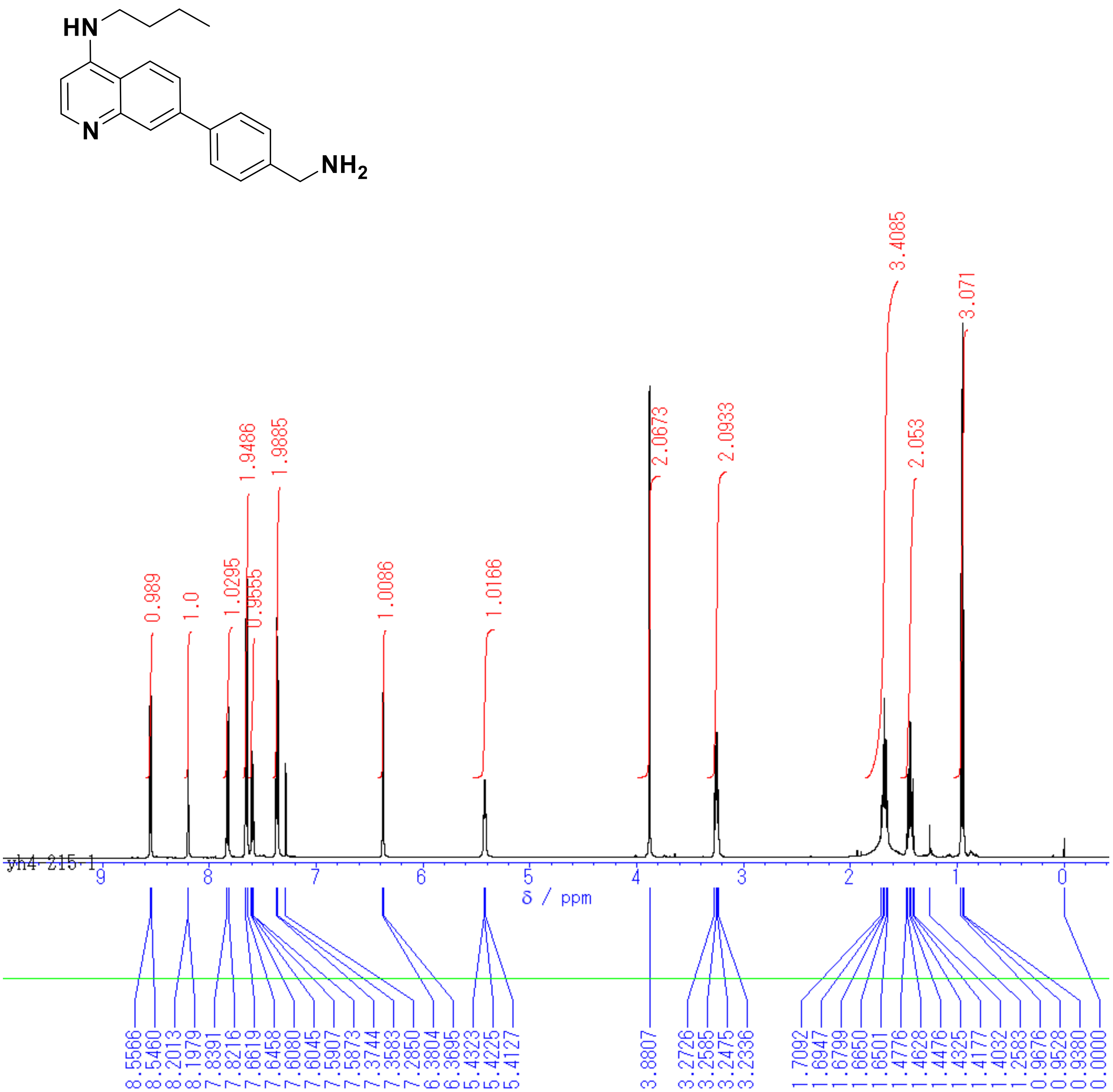

Figure S49. ^{1}H NMR spectrum ($CDCl_3$) of **11.**

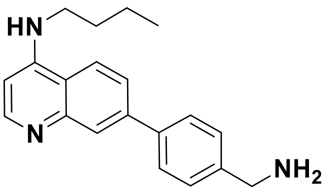

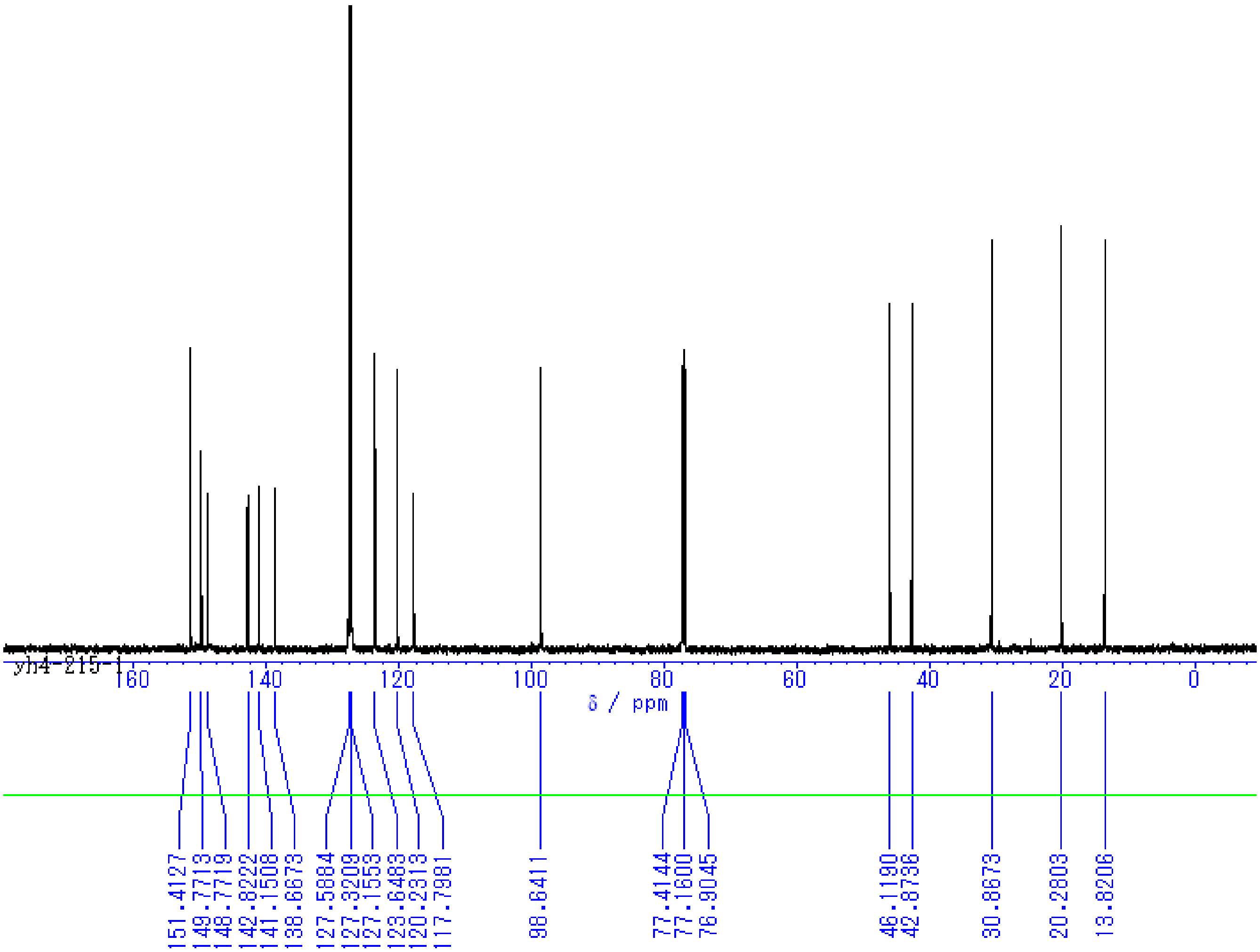

Figure S50. ^{13}C NMR spectrum ($CDCl_3$) of **11.**
